# Accounting for kin sampling reveals genetic connectivity in Tasmanian and New Zealand school sharks, *Galeorhinus galeus*

**DOI:** 10.1002/ece3.5012

**Published:** 2019-04-01

**Authors:** Floriaan Devloo‐Delva, Gregory E. Maes, Sebastián I. Hernández, Jaime D. Mcallister, Rasanthi M. Gunasekera, Peter M. Grewe, Robin B. Thomson, Pierre Feutry

**Affiliations:** ^1^ Oceans and Atmosphere CSIRO Hobart Tasmania Australia; ^2^ School of Natural Sciences – Quantitative Marine Science University of Tasmania Hobart Tasmania Australia; ^3^ Centre for Sustainable Tropical Fisheries and Aquaculture – Comparative Genomics Centre, College of Marine and Environmental Sciences James Cook University Townsville Queensland Australia; ^4^ Laboratory of Biodiversity and Evolutionary Genomics KU Leuven Leuven Belgium; ^5^ Center for Human Genetics, UZ Leuven – Genomics Core KU Leuven Leuven Belgium; ^6^ Biomolecular Lab, Center for International Program Universidad Veritas San José Costa Rica; ^7^ Sala de Colecciones, Facultad de Ciencias del Mar Universidad Católica del Norte Coquimbo Chile; ^8^ Fisheries and Aquaculture Centre, Institute for Marine and Antarctic Studies University of Tasmania Hobart Tasmania Australia

**Keywords:** close kin, genetic structure assessment, population genomics, sampling bias, shark fisheries, single nucleotide polymorphisms

## Abstract

Fishing represents a major problem for conservation of chondrichthyans, with a quarter of all species being overexploited. School sharks, *Galeorhinus galeus*, are targeted by commercial fisheries in Australia and New Zealand. The Australian stock has been depleted to below 20% of its virgin biomass, and the species is recorded as Conservation Dependent within Australia. Individuals are known to move between both countries, but it is disputed whether the stocks are reproductively linked. Accurate and unbiased determination of stock and population connectivity is crucial to inform effective management. In this study, we assess the genetic composition and population connectivity between Australian and New Zealand school sharks using genome‐wide SNPs, while accounting for non‐random kin sampling. Between 2009 and 2013, 88 neonate and juvenile individuals from Tasmanian and New Zealand nurseries were collected and genotyped. Neutral loci were analyzed to detect fine‐scale signals of reproductive connectivity. Seven full‐sibling groups were identified and removed for unbiased analysis. Based on 6,587 neutral SNPs, pairwise genetic differentiation from Tasmanian and New Zealand neonates was non‐significant (*F*_ST_ = 0.0003, CI_95_ = [−0.0002, 0.0009], *p* = 0.1163; *D*_est_ = 0.0006 ± 0.0002). This pattern was supported by clustering results. In conclusion, we show a significant effect of non‐random sampling of kin and identify fine‐scale reproductive connectivity between Australian and New Zealand school sharks.

**OPEN RESEARCH BADGES:**

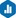

This article has earned an Open Data Badge for making publicly available the digitally‐shareable data necessary to reproduce the reported results. The data is available at https://doi.org/10.5061/dryad.pd8612j.

## 1 INTRODUCTION

Among marine organisms, sharks are of the highest conservation concern; 25% of all chondrichthyan species being currently at risk of extinction (Dulvy et al., 2014). These species are particularly vulnerable to targeted or by‐catch fisheries, partly because of late maturity and small litter size (Kyne, Bax, & Dulvy, 2015). School sharks (*Galeorhinus galeus*; Linnaeus, 1758) have been intensively fished throughout Australian waters since the 1920s for their oily livers and later on for their meat (Olsen, 1954). By the 1950s, there was concern that overfishing had depleted the stock of this species with low biological productivity (i.e., 15–43 pups every 2 years; AFMA, 2015; Olsen, 1984), causing a shift toward targeting the faster reproducing gummy shark (*Mustelus antarcticus*; Günther, 1870) (Walker, 1999). However, school shark catch continued and the stock is currently estimated to lie between 8% and 17% of the pristine level (Thomson, 2012; Thomson & Punt, 2009). Consequently, school shark has been listed as Conservation Dependent under the Environment Protection and Biodiversity Conservations Act (EPBC Act, 1999). Globally, the species is recorded as Vulnerable on the IUCN Red List (Walker et al., 2006) and has recently been designated as a priority for conservation (Dulvy et al., 2017).

Management of highly migratory species, such as school shark, presents difficulties given that international agreements may be needed to properly manage shared stocks (Fowler, 2014). Consequently, straddling stocks are sometimes managed on a less appropriate national scale. Such a problem may exist for school sharks, which are managed independently in Australia and in New Zealand (Francis, 2010), despite tagging and genetics studies that have questioned the assumption of separate stocks. Individuals are reported crossing the Tasman Sea and migrating up to 4,500 km (Coutin, Bruce, & Paul, 1992; Francis, 2010; Hurst, Baglet, McGregor, & Francis, 1999; McMillan, Huveneers, Semmens, & Gillanders, 2018). Nevertheless, such tagging studies do not provide any information about successful reproduction of migrants. Note, that the level of gene flow required to overcome genetic separation is much lower than that required to assume complete mixing and, hence, joint stock management (Begg & Waldman, 1999).

A lack of apparent genetic structure between these Australian and New Zealand sharks has been reported, using allozyme, mitochondrial DNA (mtDNA), and microsatellites (Hernández et al., 2015; Ward & Gardner, 1997), thus questioning the existence of impervious reproductive boundaries in this region. However, a more recent study, with the mitochondrial and similar nuclear microsatellite markers, found a clear separation in the microsatellite data between Tasmania and New Zealand (Bester‐van der Merwe et al., 2017). Single nucleotide polymorphisms (SNPs) have been shown to outperform microsatellites in population discrimination due to their random spread across the genome, lower ascertainment bias, higher accuracy and resolution, reproducibility, and comparability (Andrews, Good, Miller, Luikart, & Hohenlohe, 2016; Fischer et al., 2017; Muñoz et al., 2017; Seeb et al., 2011). Single nucleotide polymorphisms allow for a relatively cheap and easy way to obtain a full genome scan (Andrews et al., 2016). The large number of markers permits the inference of kinship with high certainty, investigation of population structure at higher resolution (Feutry et al., 2017), and accurate calculation of genetic diversity (as argued by Domingues, Hilsdorf, & Gadig, 2018).

In highly migratory species, sampling adults can introduce bias due to dispersal of individuals after birth and hence decreases the signal to noise ratio (Waples, 1998). This realized dispersal is much lower in neonate and juvenile school sharks (Olsen, 1954) and studying them should improve the power to detect fine‐scale structure. However, sampling juveniles result in a higher risk of generating a false signal of genetic structure through the “Allendorf–Phelps effect” (Allendorf & Phelps, 1981; Waples, 1998), due to biased sampling toward family members. Additionally, the presence of family members within a sample set has been reported to artificially increase the number of distinct genetic pools detected by clustering algorithms commonly used in population structure studies (Anderson & Dunham, 2008). Both biases have been previously reported in sharks (Feutry et al., 2017).

This study aims at testing the hypothesis of a single panmictic population of school shark between Tasmanian and New Zealand waters using novel genomic markers, while accounting for the “Allendorf–Phelps effect.” To investigate this, we genotyped neonates and juveniles from Tasmania and New Zealand. This work provides basic knowledge for the management of this commercially important species and contributes to the discussion around sampling design and data analysis when investigating the genetic structure of highly migratory species.

## 2 MATERIAL AND METHODS

### 2.1 Sample collection

**Figure 1:**
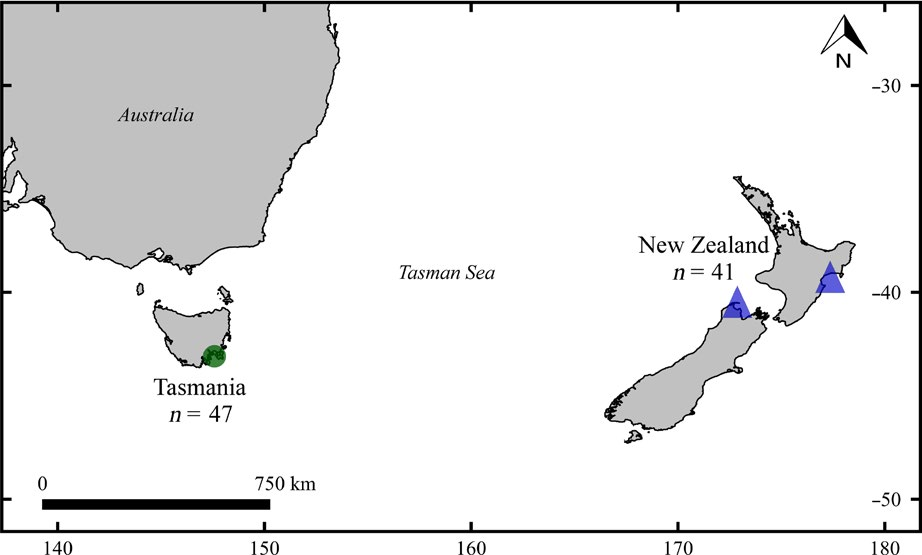
Sampling map for neonate school sharks from Tasmania and New Zealand. Green circle represents Pittwater and Norfolk Bay. Blue triangles represent Golden Bay (West, *n* = 33) and Napier (East, *n* = 8)

Eighty‐eight school sharks were collected between 2009 and 2013 using long lines and gillnets from Tasmania (TAS, *n* = 47) and New Zealand (NZ, *n* = 41) (Figure 1). Sampling sites in both countries were known nursery areas, and only neonates and juveniles (total length < 60 cm) were caught. Individuals smaller than 70 cm (i.e., 0–2 years old) are considered to have limited dispersal (Olsen, 1954). Muscle tissues or fin clips were collected and stored in ethanol. A modified version of the CTAB protocol (Doyle & Doyle, 1987; Grewe et al., 1993) was used to extract total genomic DNA.

### 2.2 SNP genotyping and filtering

Single nucleotide polymorphism genotyping was carried out by Diversity Array Technologies (DArT, Canberra, Australia) using the DArTseq^TM^ protocol, a method of sequencing complexity reduction representations. The DArTseq^TM^ protocol used in this study was identical to the one previously described by Grewe et al. (2015). The DArTseq^TM^ output consisted of 75 bp fragments containing one or more SNPs. Seventeen samples were genotyped twice to assess genotyping reproducibility.

Quality filtering was performed in R v3.5.1 (R Core Team, 2016), using the dartR v1.1.6 (Gruber, Unmack, Berry, & Georges, 2018) and the Adegenet v2.1.1 (Jombart & Ahmed, 2011) packages. Low call rate (proportion of scored loci for an individual) and high heterozygosity may indicate bad DNA quality or sample contamination, respectively. Therefore, individuals with call rate below 95% and/or heterozygosity above 20% were removed from the dataset prior to proceeding to the SNP filtering step of the data quality check process. Single nucleotide polymorphisms with a call rate (proportion of scored individuals for a locus) lower than 95%, a genotyping reproducibility below 98%, and a minor allele frequency lower than 5% were removed (Table 1). Further, loci with an average read depth lower than 15 and higher than 90 sequences per locus were filtered out. Monomorphic loci (fixed over all individuals) were deleted, since they contain no discriminating information. Outlier analysis was performed with OutFLANK v0.2 (Whitlock & Lotterhos, 2015) at a “*q* value” of 0.01, and significant outliers were removed in order to only retain neutral markers. All the cutoff values used in these filtering steps were defined after plotting the data to observe the loci/individuals’ distributions (see Supporting Information S1).

**Table 1:** Quality‐filtering steps for loci and sharks

	With full siblings		Without full siblings	
	Loci	Sharks	Loci	Sharks
Start	31,550	88	31,550	77
Multiple loci on the same sequence	24,504	88	24,504	77
Monomorphic loci	21,275	88	20,951	77
Locus call rate ≥ 0.95 & Shark call rate ≥ 0.95	13,931	88	13,579	77
Shark heterozygosity ≥ 0.20	13,931	87	13,579	76
Monomorphic loci	13,918	87	13,555	76
Average reproducibility ≤ 0.98	13,581	87	13,237	76
Coverage ≤ 15 reads	13,439	87	13,103	76
Coverage ≥ 90 reads	13,363	87	13,031	76
Minor allele frequency ≤ 0.05	6,768	87	6,603	76
Locus observed heterozygosity ≥ 0.6	6,763	87	6,594	76
Outlier loci	6,760	87	6,587	76

Moreover, two datasets (with and without siblings) were created to test the effect of non‐random sampling of siblings (Table 1). Sibship (full‐ and half‐sibling relationships) among all individuals was checked with Colony2 v2.0.6.1 (Jones & Wang, 2010) using the initially filtered dataset (see Supporting Information S2 for the analysis parameters). To build the second dataset, only one individual per sibling group was kept prior to re‐filtering all SNPs (following similar filtering steps).

### 2.3 Population diversity and structure analyses

Genetic diversity, fixation (*F*_st_), and allelic differentiation (Jost's *D* or *D*_est_) indices were calculated with diveRsity v1.9.90 (Keenan, McGinnity, Cross, Crozier, & Prodöhl, 2013), StaMPP v1.5.1 (Pembleton, Cogan, & Forster, 2013) and mmod v1.3.3 (Winter, 2012) packages, respectively, applying a bootstrap of 10,000. Population structuring was assessed with a Discriminant Analysis of Principal Components (DAPC, Adegenet v2.1.1; Jombart & Ahmed, 2011) and STRUCTURE v2.3.4 (Pritchard, Stephens, & Donnelly, 2000). With DAPC, the optimal number of clusters (*K*) was determined by the lowest Bayesian Information Criterion (BIC), and a successive *K*‐means algorithm was used to group the sharks according to this number of clusters. The optimal number of principal components retained for the DAPC analysis was selected through cross‐validation with a 10% hold‐out set and 10,000 replicates. The admixture model of STRUCTURE was applied with correlated allele frequencies for 100,000 burn‐in and 500,000 replicate runs. The program was set to assess structure between one to nine putative populations (*K*) with 20 iterations for each *K*. The optimal *K* was assessed based on the mean estimated natural logarithm of the probability (lnP). Except for the STRUCTURE analyses, all data filtering and analyses were performed and visualized using R v3.5.1 (R Core Team, 2016).

## 3 RESULTS

### 3.1 Data filtering

An average of 2,028,777 sequences per sample was obtained and the DArTsoft 2014 pipeline identified 31,550 SNPs. One individual from TAS with an excess of heterozygous loci compared to other sharks, probably due to for cross‐contamination, was removed from the data. For these 87 sharks, a total of 6,760 neutral SNPs passed all the filtering steps. Sibship analysis of this dataset revealed seven full‐sibling groups (but no half siblings) among the TAS neonates. One individual from each of the seven full‐sibling groups was retained (11 removed) to avoid biased clustering of family members. This resulted in a total of 76 neonate and juvenile sharks. After all filtering steps, 6,587 neutral SNPs were available for analysis.

### 3.2 With full sibs

**Figure 2:**
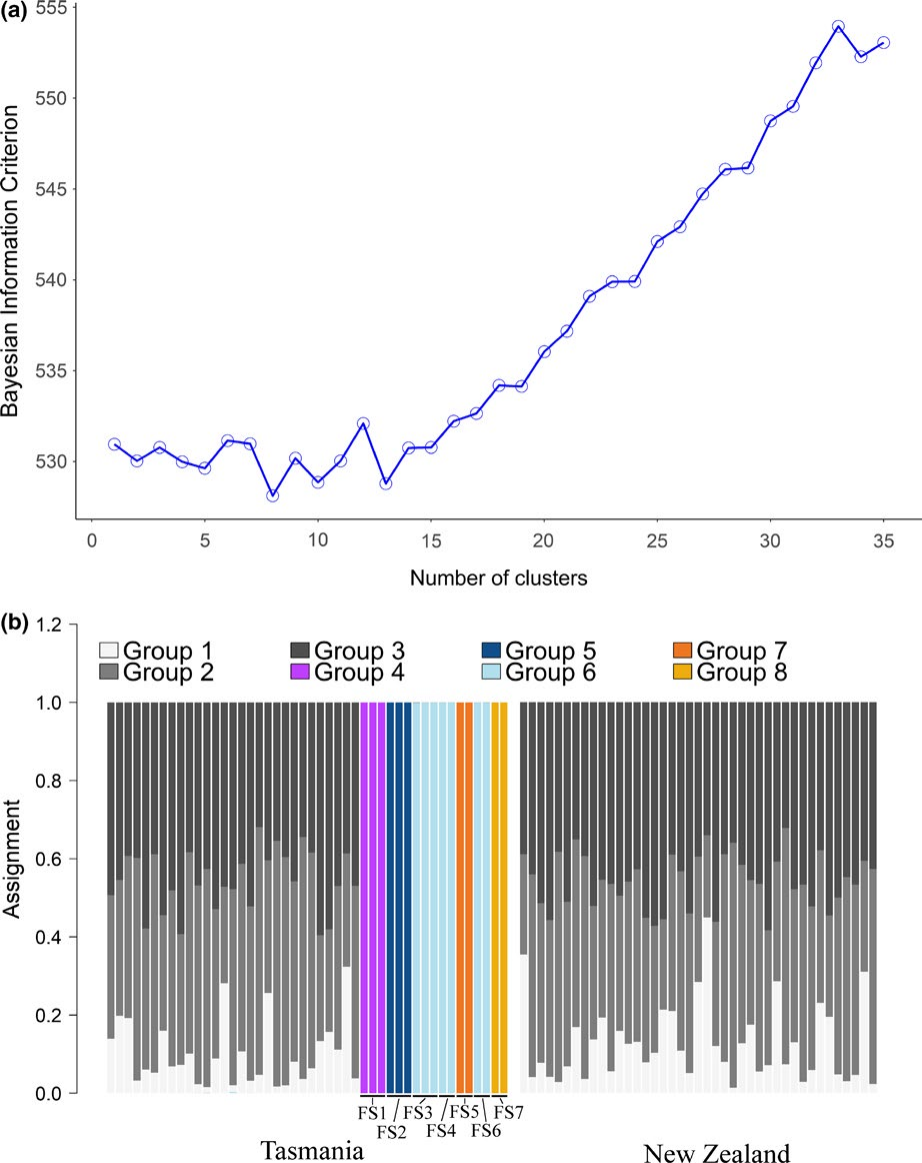
(a) Optimal number of cluster selection, based on Bayesian Information Criterion with 29 PCs. (b) DAPC assignment plot between Tasmania and New Zealand (full siblings included), based on seven PCs

Genetic diversity indices were similar for sharks from TAS and NZ. (Table 2). The fixation and differentiation indices for the neutral SNPs indicated a significant genetic difference between TAS and NZ (*F*_ST_ = 0.0023, CI_95_ = [0.0017, 0.0028], *p* = 0.0000; *D*_est_ = 0.0014 ± 0.0002). However, this signal was not visible from the DAPC plot, where the BIC indicated that eight groups seemed to be the optimal solution (Figure 2a). Five of those eight groups were comprised of full siblings, and no differentiation between TAS and NZ could be found (Figure 2b). The sibling‐driven clustering was not as obvious in the STRUCTURE as in the DAPC results; with a similar likelihood for *K* = 1, 2, 5, or 7 (Supporting Information S3).

**Table 2:** Genetic diversity of 87 (6,760 SNPs) and 76 (6,587 SNPs) sharks, respectively

	With full siblings			Without full siblings		
	Overall	TAS	NZ	Overall	TAS	NZ
*N*	87	46	41	76	35	41
*H*_o_	0.263	0.264	0.262	0.265	0.265	0.264
*H*_E_	0.285	0.285	0.284	0.285	0.284	0.285
*F*_IS_	0.068	0.070	0.069	0.066	0.065	0.067
*A*_R_	1.995	1.995	1.994	1.992	1.990	1.993

Note

*N*, sample size; *H*_O_, observed heterozygosity; *H*_E_, expected heterozygosity; *F*_IS_, inbreeding coefficient; *A*_R_, allelic richness.

### 3.3 Without full sibs

**Figure 3:**
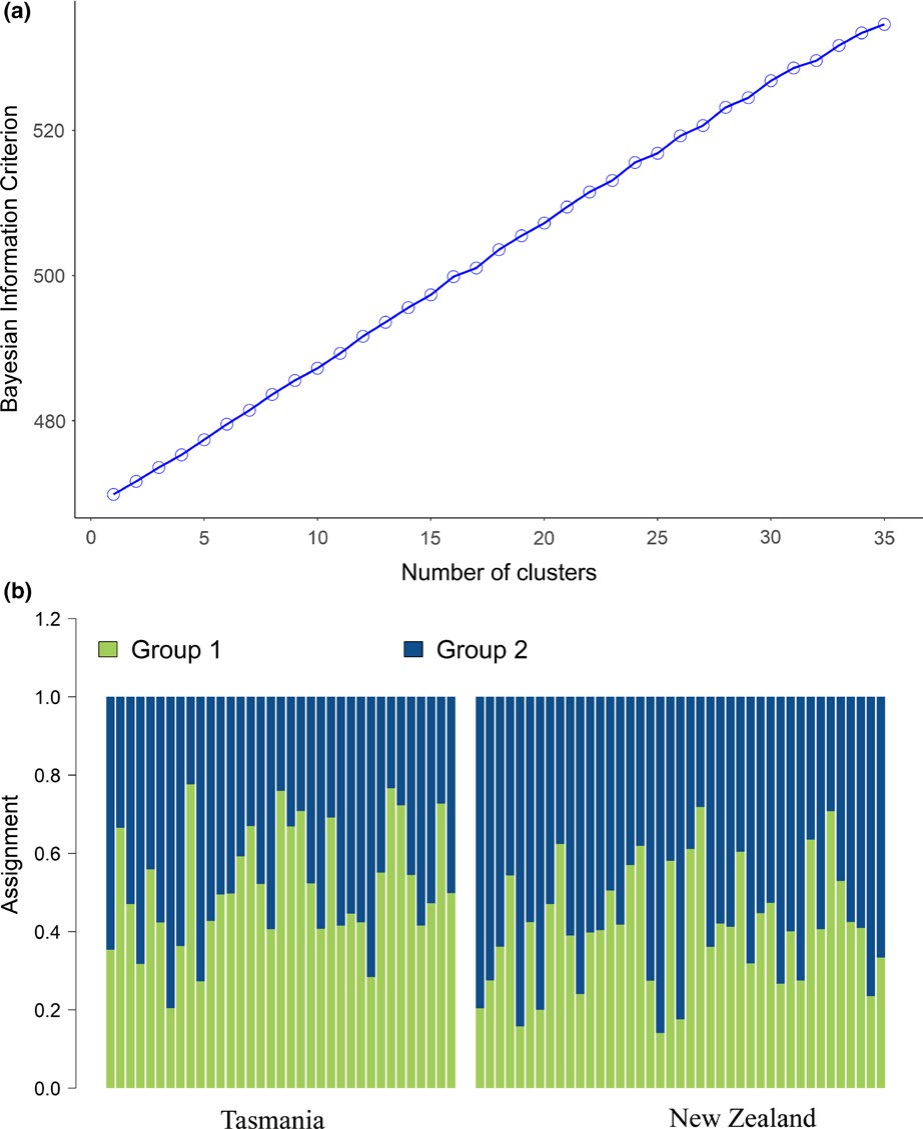
(a) Optimal number of cluster selection, based on Bayesian Information Criterion with 25 PC's. (b) DAPC assignment plot between Tasmania and New Zealand (full siblings excluded), based on 35 PCs

Neutral genetic diversity decreased slightly, but non‐significantly, compared to the dataset with full siblings and did not show any differences between TAS and NZ (Table 2). Pairwise *F*_ST_ became non‐significant (*F*_ST_ = 0.0003, CI_95_ = [−0.0002, 0.0009], *p* = 0.1163; *D*_est_ = 0.0006 ± 0.0002) and based on the BIC of the DAPC and the mean lnP of the STRUCTURE analysis, one population seemed to be the best clustering solution (Figure 3a, Supporting Information S4). This result is supported by the lack of visible structure in the DAPC (Figure 3b) and STRUCTURE plots (Supporting Information S4).

## 4 DISCUSSION

### 4.1 Population structure with or without siblings?

The conclusions drawn from this study greatly depend on which dataset is interpreted (with or without full siblings). By removing full‐sibling groups from the dataset, the *F*_ST_ value decreased by one order of magnitude and the optimal number of clusters decreased from eight to one (Figures 2a and 3a). If the sibling groups are left in the dataset, there is a risk of misinterpreting population structure for what is actually family structure. However, Waples and Anderson (2017) demonstrated that the trending common practice, consisting of purging groups of siblings prior to population genetic analyses, can introduce a bias if the presence of these groups is not a sampling artifact but rather the result of a small localized population. Removing the right amount of closely related individuals is theoretically feasible, but requires knowledge of (at least) the effective population size. Unfortunately, family structure also creates a bias when estimating this quantity (Waples & Anderson, 2017), which makes it a circular issue. In this study, all full siblings were sampled within the same year, with a maximum of four months between captures, which indicates that their presence is a sampling artifact. Another indicator of a family sampling bias is the absence of half siblings. If the presence of such a high proportion of full siblings in Tasmania was due to a small and localized population and given that males are not believed to be monogamous and that females are expected to reproduce more than once across the sampling period (Walker, 2005), one would have expected to detect half siblings too. More likely, the presence of full sibs in this dataset reflects a higher probability of sampling litter mates (individuals having the same mother and born at the same place and time). Due to interdependence between effective population size, population structure, and family structure, we suggest repetitive sampling over time can help interpret population structure in the presence of family members.

### 4.2 Population structure compared to previous studies

Interestingly, our findings contradict nuclear DNA results from a recent study of Bester‐van der Merwe et al. (2017). Potential sibling‐ or sex‐biased sampling could explain the observed nuclear signal of structure (Allendorf & Phelps, 1981; Benestan et al., 2017; Feutry et al., 2017; Waples, 1998). School sharks are known to school by size and sex (Francis, 2010; Olsen, 1984). The nine Tasmanian and 20 New Zealand individuals from Bester‐van der Merwe et al. (2017) were obtained to identify biased sampling. We were unable to test the sex‐biased sampling hypothesis, because of missing sex information, but we re‐analyzed the 19 microsatellites in COLONY2. Eight pairs of individuals had a probability over 75% of being either full or half siblings; settings and results are presented in Supporting Information S2 and S5. Due to the low sample size and missing alleles, a reliable estimate of allele frequencies could not be made and these results must be interpreted with caution. In addition, a recent publication from McMillan et al. (2018) described partial migratory behavior of Australian school sharks, where some females appeared to be resident. Consequently, the possibility of a small and localized population in Tasmania cannot be excluded.

This study builds on the many telemetry and genetic studies that have investigated movement and connectivity of school sharks within Oceania (Bester‐van der Merwe et al., 2017; Coutin et al., 1992; Hernández et al., 2015; Hurst et al., 1999; McAllister, Barnett, Lyle, & Semmens, 2015; McMillan et al., 2018; Olsen, 1954; Ward & Gardner, 1997). Based on current results, the null hypothesis of a single panmictic population cannot be rejected. Both *F*_ST_ and *D*_est_, as well as diversity and clustering analyses, did not detect differentiation between TAS and NZ neonates and juveniles. This is supported by the large dispersal abilities of school sharks (Coutin et al., 1992; Hurst et al., 1999; McAllister et al., 2015; McMillan et al., 2018; Olsen, 1954). Genetic diversity was similar between both sampling regions, but lower compared to previous studies (He = 0.5–0.75; Hernández et al., 2015; Bester‐van der Merwe et al., 2017; Domingues et al., 2018). This discrepancy with other studies can be explained by the choice of genetic markers. This study presents the first genomic study of school sharks and in theory allows a more accurate calculation of genetic diversity (Fischer et al., 2017). Overall, our diversity measures correspond to other genomic studies in sharks (Feutry et al., 2017; Maisano Delser et al., 2018; Pazmiño et al., 2018). Furthermore, Ward and Gardner (1997) found weak evidence of genetic differentiation; however, this was based on a single allozyme and mitochondrial DNA markers. Hernández et al. (2015) showed the presence of a single genetic population in Oceania, using mtDNA and microsatellites. With increased power of genome‐wide SNPs, we found similar results. The observed signal could also be attributed to other explanations that could not be identified with our current sampling design: (a) a high gene flow that dilutes existing, recent population differentiation (Bailleul et al., 2018; Waples & Gaggiotti, 2006), (b) sex‐biased dispersal where one sex obscures the philopatric signal (Fraser, Lippé, & Bernatchez, 2004) or (c) temporal structure caused by their biennial–triennial pupping behavior (Waples, 1998).

### 4.3 Future work

The use of neonate and juvenile samples in this study is ideal to detect population structure in highly migratory species, but our sampling design and choice of markers did not allow us to fully investigate potential temporal‐ or sex‐biased dispersal. Regional female philopatry has been suggested by Bester‐van der Merwe et al. (2017) in South Africa; however, this has not yet been observed in Oceania (Francis, 2010; Hernández et al., 2015). Hernández et al. (2015) did not detect any sign of philopatry using mitochondrial markers, but using whole mitogenome sequences instead of the control region might provide better insight (Feutry et al., 2014). Paternally (Y‐chromosome) inherited markers or the spatial distribution of siblings may also help detecting sex‐biased dispersal (Feutry et al., 2017; Petit, Balloux, & Excoffier, 2002). Moreover, Pittwater, Tasmania, is currently the only known school shark nursery area in Australia where pups can reliably be caught (others in Tasmania and Victoria currently yielding few or no pups). However, samples from other nurseries closer to the mainland of Australia and multi‐year sampling could possibly reveal population structure between other regions of Australia and New Zealand. In any case, given the highly migratory nature of adult school sharks, such fine‐scale structure, if it existed, would only impact management practices if nurseries areas were to be targeted by the fishing fleet, which is not the case.

## 5 CONCLUSION

In conclusion, this study has illustrated how kin bias can affect population structure inference if sampling is not randomly spread and proposed several measures how to identify such biased sampling toward kin. The unbiased estimates of population connectivity could not reject the existence of a panmictic population between Tasmania and New Zealand school sharks; yet possible caveats in the study have been pinpointed and the presence of small local populations may still be plausible. Overall, due to the migratory behavior of school sharks we argue that potential population structure would only form a conservation issue if nursery areas would be targeted by fisheries, which they currently are not.

## CONFLICT OF INTEREST

The authors declare no conflicts of interest.

## AUTHOR CONTRIBUTION

FD, GM, PG, RT, and PF designed the study. Samples were acquired by SH and JM. RG extracted DNA from the samples. FD analyzed the data with contribution from PF. The manuscript was drafted by FD. All authors reviewed the manuscript and gave final approval for publication. All authors agree to be accountable for all aspects of the work.

## Data Availability

Raw and filtered SNPs with associated metadata. Data for this study are available at: https://doi.org/10.5061/dryad.pd8612j
