# Supplementary material for: Accounting for kin sampling reveals genetic connectivity in Tasmanian and New Zealand school sharks, Galeorhinus galeus
Source: Ecol Evol. 2019 Apr 1;9(8):4465–72. doi: 10.1002/ece3.5012 (PMC6476751; doi:10.1002/ece3.5012)
Supplement: Supplementary file 1 [file ECE3-9-4465-s001.pdf]

Devloo-Delva et al. Supporting Information S1: Full analysis in Rmarkdown

Floriaan Devloo-Delva and Pierre Feutry

Jan 2019

- 1 Background
- 2 Sampling map
- 3 Load packages
- 4 First analysis: With full-sibs
  - 4.1 Load data
  - 4.2 Sample summary
  - 4.3 Filtering
    - 4.3.1 Filter for duplicate loci/CloneID
    - 4.3.2 Monomorphic loci
    - 4.3.3 Filter on Callrate
    - 4.3.4 Individual Heterozygosity
    - 4.3.5 Monomorphic loci
    - 4.3.6 Filter on average reproducability
    - 4.3.7 Counts
    - 4.3.8 Minor allele frequency
    - 4.3.9 Heterzygozity per SNP
  - 4.4 Convert to other data types
  - 4.5 Calculate outliers
    - 4.5.1 OutFLANK
    - 4.5.2 PCadapt
    - 4.5.3 Remove outliers
  - 4.6 Export data to other software formats
  - 4.7 Genetic diversity
  - 4.8 Fixation and differentiation indices
    - 4.8.1 Global
    - 4.8.2 Pairwise
  - 4.9 DAPC
    - 4.9.1 DAPC with location prior
    - 4.9.2 DAPC without location prior
  - 4.10 STRUCTURE output
- 5 Second analysis: Without full-sibs
  - 5.1 Load data
  - 5.2 Remove full siblings
  - 5.3 FSP summary
  - 5.4 Filtering
    - 5.4.1 Filter for duplicate loci/CloneID
    - 5.4.2 Monomorphic loci
    - 5.4.3 Filter on Callrate
    - 5.4.4 Individual Heterozygosity
    - 5.4.5 Monomorphic loci
    - 5.4.6 Filter on average reproducability
    - 5.4.7 Counts
    - 5.4.8 Minor allele frequency
    - 5.4.9 Heterzygozity per SNP
  - 5.5 Convert to other data types
  - 5.6 Calculate outliers
    - 5.6.1 OutFLANK
    - 5.6.2 PCadapt
    - 5.6.3 Remove outliers
  - 5.7 Export data to other software formats
  - 5.8 Genetic diversity
  - 5.9 Fixation and differentiation indices
    - 5.9.1 Global
    - 5.9.2 Pairwise
  - 5.10 DAPC

- 5.10.1 DAPC with location prior
- 5.10.2 DAPC without location prior
- 5.11 STRUCTURE output
- 5.12 Assignment test
- 5.13 Sex-biased gene flow
- 6 Citations for packages
- 7 Session info

1 Background

This Rmarkdown document described the data-filtering and analysis steps for assessing the population structure of school sharks and kinship bias in Devloo-Delva et al. (2019).

2 Sampling map

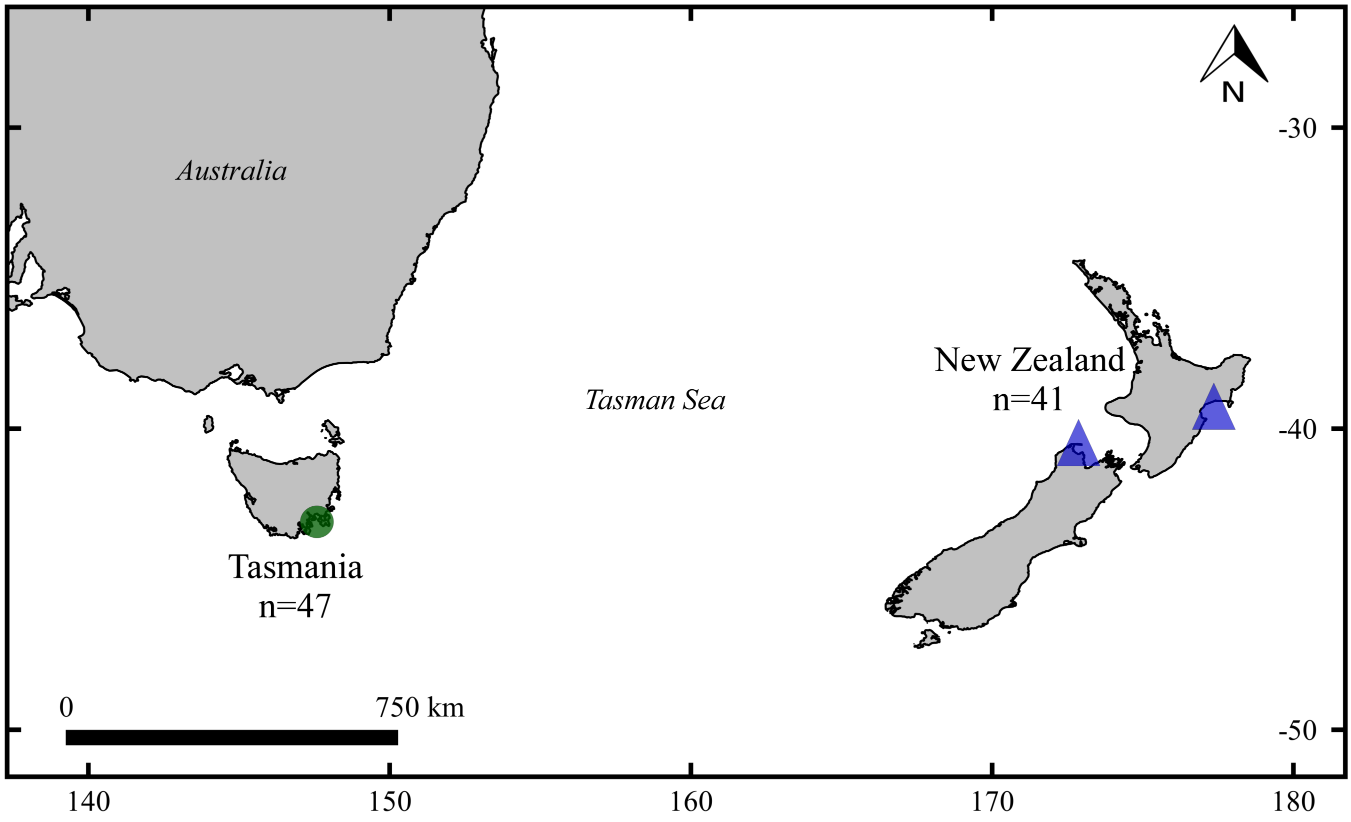

Sampling map for neonate school sharks from Tasmania and New Zealand. Green circle represents Pittwater and Norfolk Bay. Blue triangles represent Golden Bay (West, n=33) and Napier (East, n=8).

3 Load packages

```r
library(ade4)
library(adegenet)
library(SNPRelate)
library(dartR)
library(pegas)
library(assigner)
library(ape)
library(hierfstat)
library(plyr)
library(diveRsity)
library(StAMPP)
library(mmod)
library(ggplot2)
library(data.table)
library(vioplot)
library(qvalue)
library(OutFLANK)
library(pcadapt)
source("Filtering funtions.R")  #script with personal filtering functions

# Setting for bootstrap throughout the document
boots <- 10000
boots2 <- 100
```

4 First analysis: With full-sibs

4.1 Load data

```r
dartfile <- "DGale15_1833_1863_SNP_CallRate_0.1-Edited2.csv"
meta <- "schoolshark_meta2.csv"
lastmetric <- "TotalPicRepSnpTest"

gl <- dartR::gl.read.dart(filename = dartfile, covfilename = meta,
    topskip = 0, lastmetric = lastmetric, probar = F)
```

```
## Trying to determine if one row or two row format...
## Found  1  row(s) format. Proceed...
## Added the following covmetrics:
## AlleleID CloneID ClusterTempIndex AlleleSequence ClusterConsensusSequence ClusterSize
AlleleSeqDist SNP SnpPosition CallRate OneRatioRef OneRatioSnp FreqHomRef FreqHomSnp FreqHets PICRef
PICSnp AvgPIC AvgCountRef AvgCountSnp SumCount RatioAvgCountRefAvgCountSnp FreqHetsMinusFreqMinHom A
lleleCountsCorrelation aggregateTagsTotal DerivedCorrMinusSeedCorr RepRef RepSNP RepAvg PicRepRef Pi
cRepSNP TotalPicRepRefTest TotalPicRepSnpTest .
## Number of rows per Clone. Should be only  1 s: 1
##  Recognised: 88 individuals and 31550  SNPs in a 1 row format using
DGale15_1833_1863_SNP_CallRate_0.1-Edited2.csv
## Start conversion....
## Format is 1 rows.
## Please note conversion of bigger data sets will take some time!
## Once finished, we recommend to save the object using save(object, file="object.rdata")
## Try to add covariate file: schoolshark_meta2.csv .
## Ids of covariate file does not match the number of ids in the genetic file. Maybe this is fine if
a subset matches.
## Ids of covariate file (at least a subset of) are matching!
## Found  88 matching ids out of 723 ids provided in the covariate file. Subsetting snps now!.
##  Added pop factor.
## Please note:there is no lat column
## Please note:there is no lon column
## Added  id  to the other$ind.metrics slot.
##  Added  SpeciesName  to the other$ind.metrics slot.
##  Added  pop  to the other$ind.metrics slot.
##  Added  Sex  to the other$ind.metrics slot.
##  Added  Collection.Location  to the other$ind.metrics slot.
##  Added  State  to the other$ind.metrics slot.
##  Added  CollectionContact  to the other$ind.metrics slot.
##  Added  DateCollected  to the other$ind.metrics slot.
##  Added  Date  to the other$ind.metrics slot.
##  Added  Day.Collected  to the other$ind.metrics slot.
##  Added  Month.Collected  to the other$ind.metrics slot.
##  Added  Year_collected  to the other$ind.metrics slot.
##  Added  DateArrivedCSIRO  to the other$ind.metrics slot.
##  Added  Day.Arrived.CSIRO  to the other$ind.metrics slot.
##  Added  Month.Arrived.CSIRO  to the other$ind.metrics slot.
##  Added  Year.Arrived.CSIRO  to the other$ind.metrics slot.
##  Added  VesselName  to the other$ind.metrics slot.
##  Added  Log.Book.Number  to the other$ind.metrics slot.
##  Added  Page.number  to the other$ind.metrics slot.
##  Added  Length_TOT_cm  to the other$ind.metrics slot.
##  Added  Length_PAR_cm  to the other$ind.metrics slot.
##  Added  Length  to the other$ind.metrics slot.
##  Added  X  to the other$ind.metrics slot.
##  Added  FSP  to the other$ind.metrics slot.
##  Added  Sibs  to the other$ind.metrics slot.
##  Added  Siblings  to the other$ind.metrics slot.
##  Added  SexSiblings  to the other$ind.metrics slot.
##  Added  Tag.Number  to the other$ind.metrics slot.
##  Added  Sample.box.position..DNA.  to the other$ind.metrics slot.
##  Added  X.1  to the other$ind.metrics slot.
##  Added  DArT.Plate.number  to the other$ind.metrics slot.
##  Added  DTU.number  to the other$ind.metrics slot.
##  Added  DNAQuality  to the other$ind.metrics slot.
##  Added  Location.of.DNA.sample  to the other$ind.metrics slot.
##  Added  Location.of.tissue.sample  to the other$ind.metrics slot.
##  Added  Comments  to the other$ind.metrics slot.
##  Added  Sibs2  to the other$ind.metrics slot.
##  Added  Sex2  to the other$ind.metrics slot.
##  Added  yearcollect  to the other$ind.metrics slot.
##  Added  Age  to the other$ind.metrics slot.
##  Added  Cohort  to the other$ind.metrics slot.
```

```
pop.levels <- c("TAS", "NZd")
gl$pop <- factor(x = gl$pop, levels = pop.levels)
gl <- gl[order(gl$pop, gl$other$ind.metrics$FSP, gl$ind.names)]
```

4.2 Sample summary

Here we provide a visual summary of the collection date, assigned age/cohort and sex.

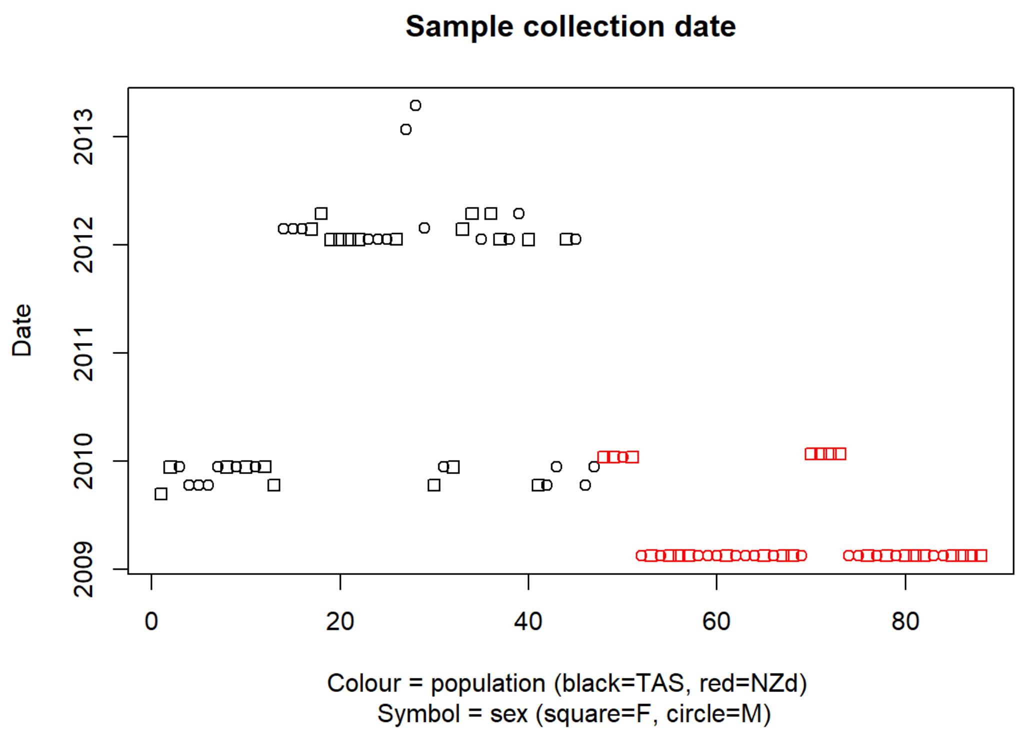

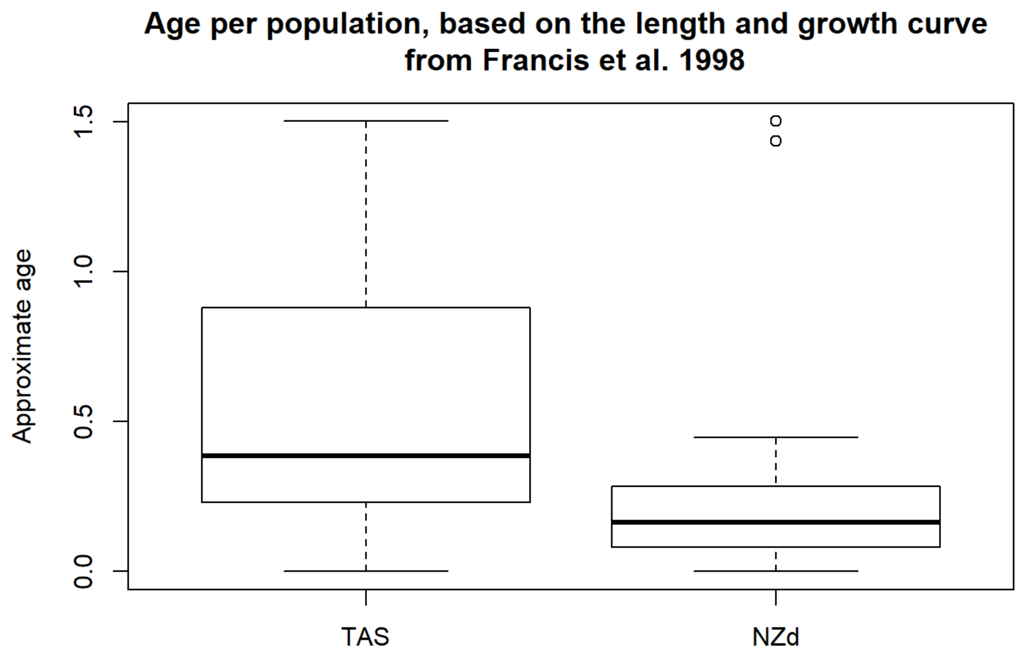

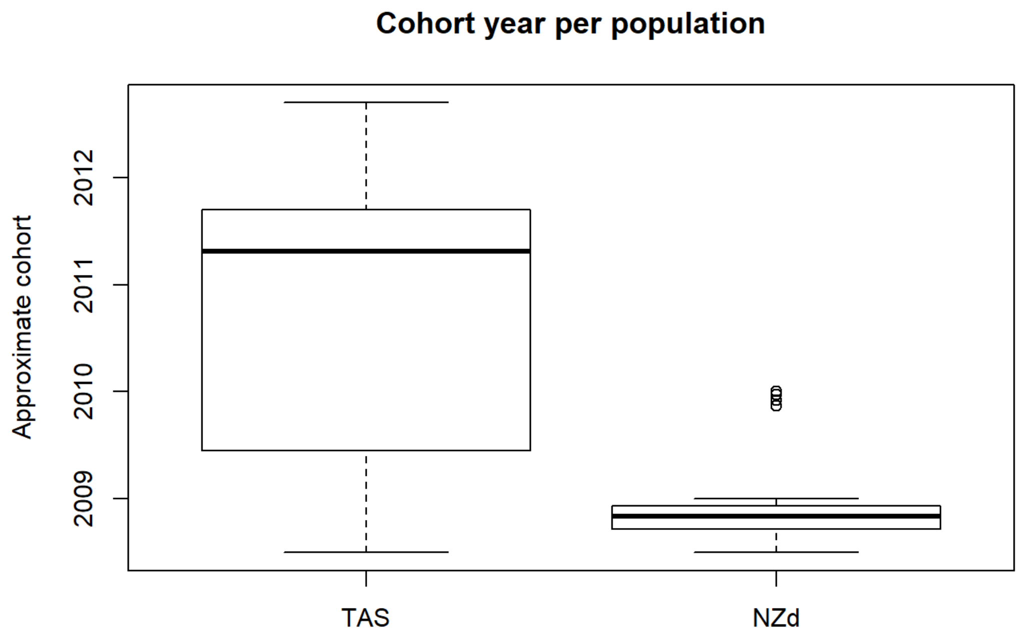

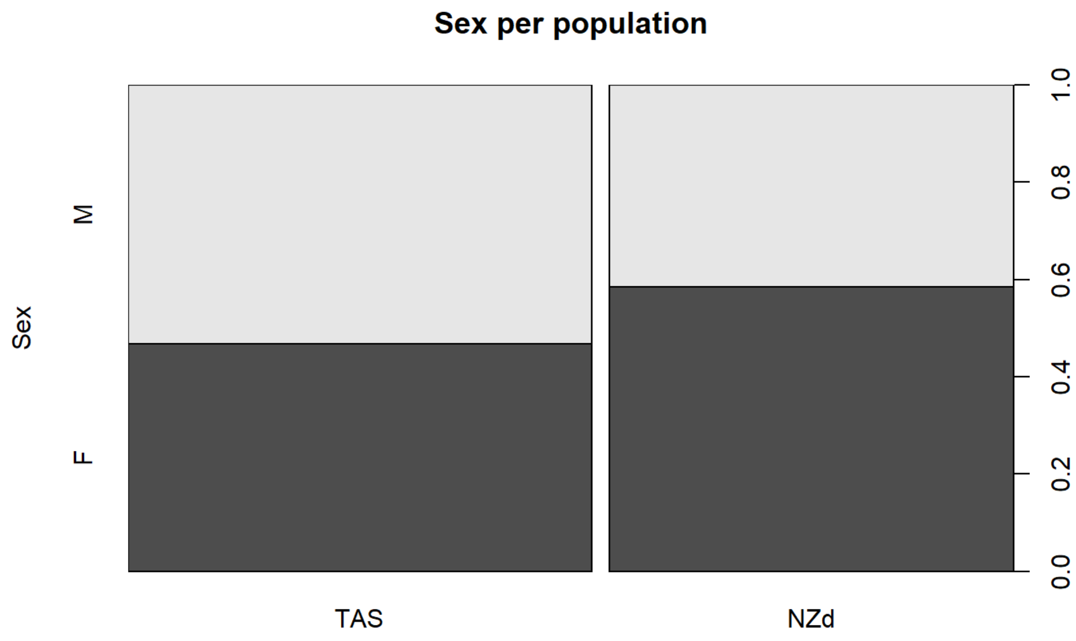

4.3 Filtering

4.3.1 Filter for duplicate loci/CloneID

```
gl <- dartR::gl.filter.secondaries(gl, v = 5)
```

```
## Starting gl.filter.secondaries: Deleting all but one SNP per sequence tag
##   Total number of SNP loci: 31550
##   Selecting one SNP per sequence tag at random
##     Number of secondaries: 7046
##     Number of loci after secondaries removed: 24504
## gl.filter.secondaries completed
```

4.3.2 Monomorphic loci

```
set.seed(124)
gl <- dartR::gl.filter.monomorphs(gl, v = 5)
```

```
## Starting gl.filter.monomorphs: Deleting monomorphic loci
##   Polymorphic loci: 21275
##   Monomorphic loci: 3229
##   Loci with no scores (all NA): 0
##   Deleting monomorphic loci and loci with all NA scores
## Completed gl.filter.monomorphs
```

4.3.3 Filter on Callrate

Checks how much missing data you have.

> If you have individuals with many missing data, it might be good to first filter on individuals (e.g. 0.80), then loci (0.80), then ind (0.85), then loci (0.85), etc....

```
dartR::gl.report.callrate(gl, method = "loc")
```

```
## Starting gl.report.callrate
## Starting utils.recalc.callrate: Recalculating CallRate
## Completed utils.recalc.callrate
```

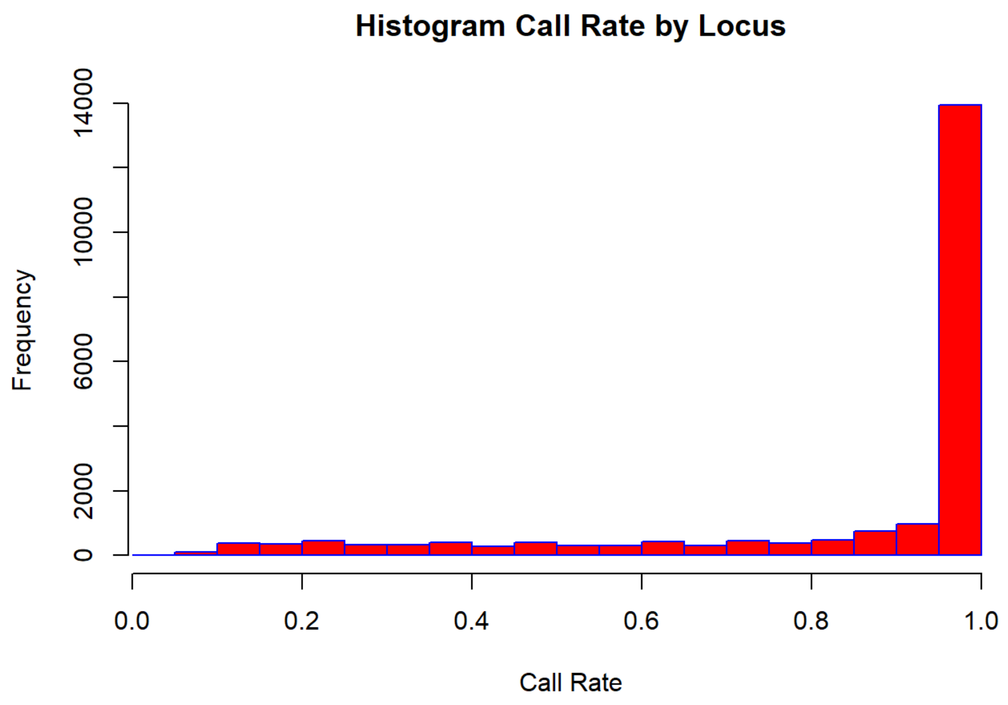

```
##   Loci with no missing values = 10979 [51.6%]
##     < 5% missing values = 13931 [65.5%]
##     < 10% missing values = 14912 [70.1%]
##     < 15% missing values = 15664 [73.6%]
##     < 20% missing values = 16133 [75.8%]
##     < 25% missing values = 16583 [77.9%]
##     < 30% missing values = 16940 [79.6%]
##     < 35% missing values = 17250 [81.1%]
##     < 40% missing values = 17682 [83.1%]
##     < 45% missing values = 17976 [84.5%]
##     < 50% missing values = 18351 [86.3%]
##     < 55% missing values = 18666 [87.7%]
##     < 60% missing values = 18944 [89%]
##     < 65% missing values = 19337 [90.9%]
##     < 70% missing values = 19655 [92.4%]
##     < 75% missing values = 20075 [94.4%]
##     < 80% missing values = 20441 [96.1%]
##     < 85% missing values = 20790 [97.7%]
##     < 90% missing values = 21179 [99.5%]
## gl.report.callrate Completed
```

```
## NULL
```

```
dartR::gl.report.callrate(gl, method = "ind")
```

```
## Starting gl.report.callrate
## Starting utils.recalc.callrate: Recalculating CallRate
## Completed utils.recalc.callrate
```

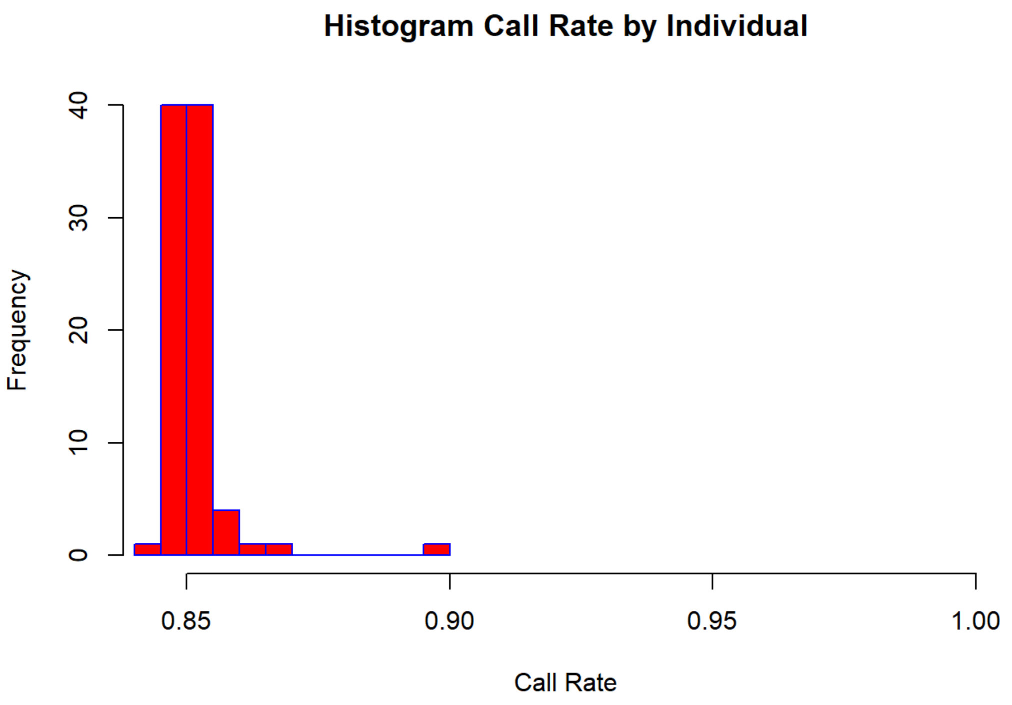


```
## Individuals no missing values = 0 [0%] across loci; all individuals would be filtered
##      with less than or equal to 5% = 0 [0%]; 88 individuals would be filtered
##      with less than or equal to 10% = 0 [0%]; 88 individuals would be filtered
##      with less than or equal to 15% = 47 [53.4%]; 41 individuals would be filtered
##      with less than or equal to 20% = 88 [100%]; 0 individuals would be filtered
## gl.report.callrate Completed
```

```
## NULL
```

```
gl <- gl.filter.missing.data.FDD(gl, loc.lb = 0.5, loc.hb = 0.95,
    ind.lb = 0.5, ind.hb = 0.95, iterations = 100)
```

```
## Summary of filtered dataset
##   SNPs with CallRate > 0.95 : 13931
##   Individuals with CallRate > 0.95 : 88
##   No. of loci removed: 7344
##   No. of individuals removed: 0
```

4.3.4 Individual Heterozygosity

Delete individuals with a heterozygosity above the threshold. High heterozygozity could be due to cross-contamination from other individuals.

```
gl <- gl.filter.het.FDD(gl, LowerT = 0.1, UpperT = 0.2)
```

```
## Reporting for a genlight object
## Initial no. of individuals = 88
## Filtering a genlight object
##   no. of individuals deleted = 1
## Individuals retained = 87
##
## List of individuals deleted because of high heterozygosity
##  SsTAS_Bx02_E10
##
##    from populations
##  TAS
##
## Summary of filtered dataset
## 0.1 <  Individuals with heterozygosity <= 0.2
##   No. of loci: 13931
##   No. of individuals: 87
##   No. of populations:  2
```

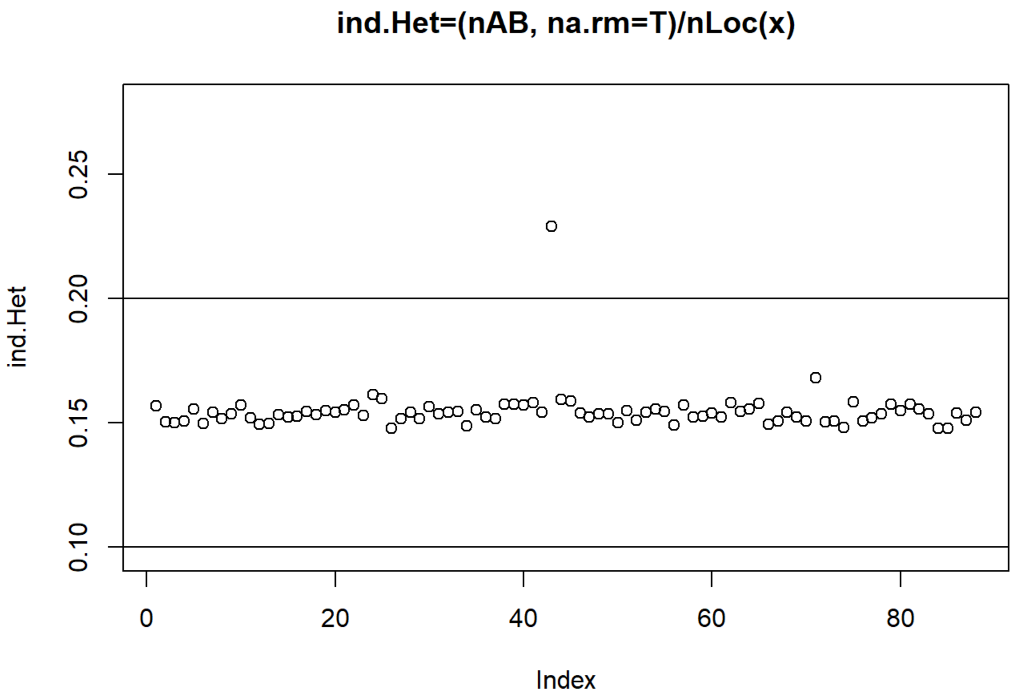

4.3.5 Monomorphic loci

```
gl <- dartR::gl.filter.monomorphs(gl, v = 5)
```

```
## Starting gl.filter.monomorphs: Deleting monomorphic loci
##   Polymorphic loci: 13918
##   Monomorphic loci: 13
##   Loci with no scores (all NA): 0
##   Deleting monomorphic loci and loci with all NA scores
## Completed gl.filter.monomorphs
```

4.3.6 Filter on average reproducability

DArT includes several duplicates of your samples to see how reproducable they results are.

```
dartR::gl.report.repavg(gl)
```

```
## Reporting for a genlight object
## Note: RepAvg is a DArT statistic reporting reproducibility averaged across alleles for each locus
.
##
## No. of loci = 13918
##
##   Loci with perfect reproducibility = 11335 [81.4%]
##   > 0.995 = 11335 [81.4%]
##   > 0.99 = 11335 [81.4%]
##   > 0.985 = 13538 [97.3%]
##   > 0.98 = 13581 [97.6%]
##   > 0.975 = 13581 [97.6%]
##   > 0.97 = 13918 [100%]
```

```
## [1] "Completed"
```

```
gl <- dartR::gl.filter.repavg(gl, t = 0.98, v = 5)
```

```
## Starting gl.filter.repavg: Filtering on repeatability
## Note: RepAvg is a DArT statistic reporting repeatability averaged across alleles for each locus.
##
## Initial no. of loci = 13918
##   Removing loci with RepAvg < 0.98
## No. of loci deleted = 337
## Summary of filtered dataset
##   Repeatability >= 0.98
##   No. of loci: 13581
##   No. of individuals: 87
##   No. of populations:  2
## gl.filter.repavg completed
```

4.3.7 Counts

Check the histogram to see how many SNP have low or high counts.

```
hist(gl@other$loc.metrics[, "SumCount"], breaks = 20, plot = T,
    main = "Histogram of read depth of reference and SNP allele")
```

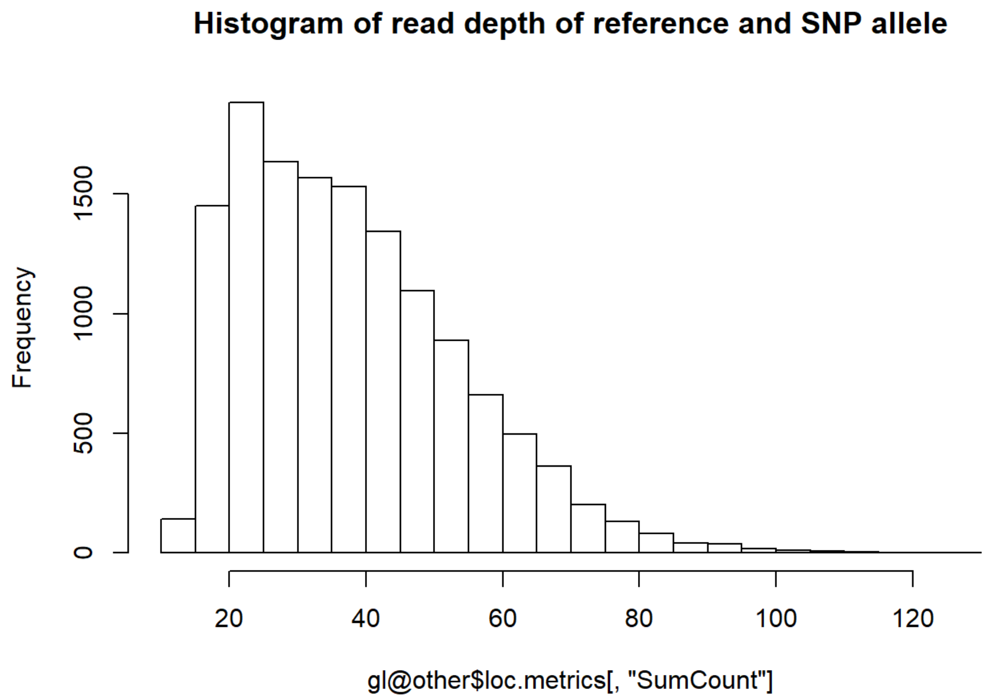

4.3.7.1 Low counts

Low counts might not be reliably genotyped.

```
gl <- gl.filter.lowcount.FDD(gl, threshold = 15)
```

```
## Total number of SNP loci: 13581
## No. of loci deleted = 142
##   Read depth >= 15
##   No. of loci: 13439
##   No. of individuals: 87
##   No. of populations:  2
```

4.3.7.2 High Counts

High counts might be due to paralogous fragments.

```
gl <- gl.filter.highcount.FDD(gl, threshold = 90)
```

```
## Total number of SNP loci: 13439
## No. of loci deleted = 76
##   Read depth <= 90
##   No. of loci: 13363
##   No. of individuals: 87
##   No. of populations:  2
```

4.3.8 Minor allele frequency

Loci with a low MAF might not be informative enough and just increase computation time.

```
gl <- gl.filter.maf.FDD(gl, threshold = 0.05)
```

```
## Filtering a genlight object
## Total number of SNP loci: 13363
##    Number of loci with MAF < 0.05 : 6595
##    Number of loci after filtering: 6768
##    Number of individuals: 87
##    Number of populations:  2
```

4.3.9 Heterzygozity per SNP

SNPs that are too heterozygous could be due to paralogous reads.

```
gl <- gl.filter.loc.het.FDD(gl, threshold = 0.6)
```

```
## Total number of SNP loci: 6768
## No. of loci deleted = 5
##   SNPs with heterozygosity <= 0.6
##   No. of loci: 6763
##   No. of individuals: 87
##   No. of populations:  2
```

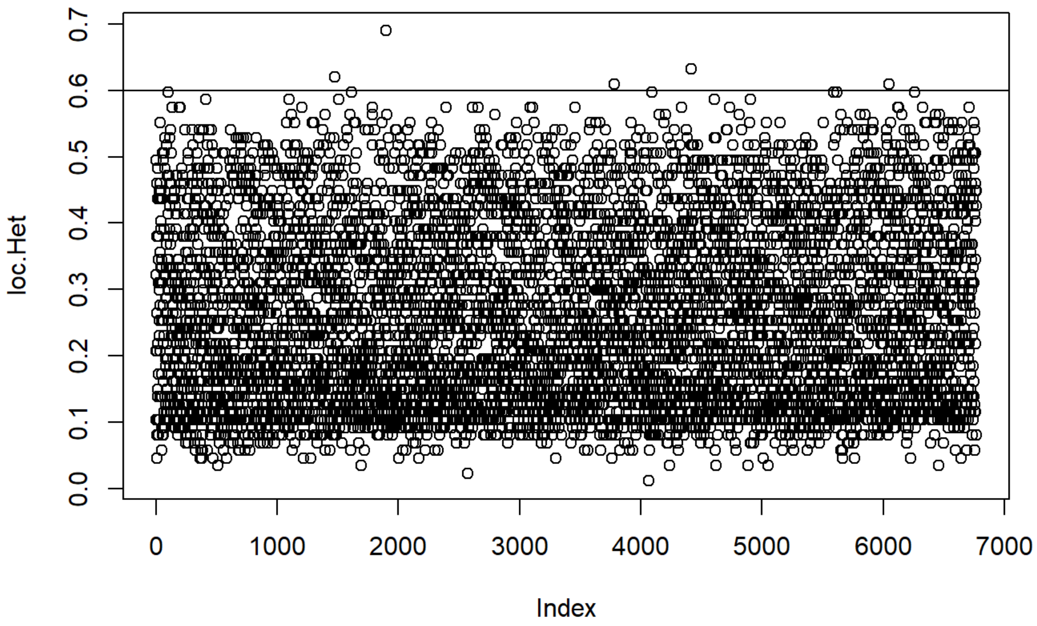

4.4 Convert to other data types

```
gi <- gl2gi_mvb(gl)
gl2Adm(gl, filename = "SS_ADMIXTURE-PCadapt")
```

4.5 Calculate outliers

4.5.1 OutFLANK

```
SNPmat <- as.matrix(gl)
colnames(SNPmat) <- NULL
row.names(SNPmat) <- NULL
SNPmat[is.na(SNPmat)] <- 9
FstDataFrame <- OutFLANK::MakeDiploidFSTMat(SNPmat, gl$loc.names,
    as.character(gl$pop))
```

```
## Calculating FSTs, may take a few minutes...
```

```
plot(FstDataFrame$FST, FstDataFrame$FSTNoCorr, xlim = c(-0.01,
    0.3), ylim = c(-0.01, 0.3), pch = 20) + abline(0, 1)
```

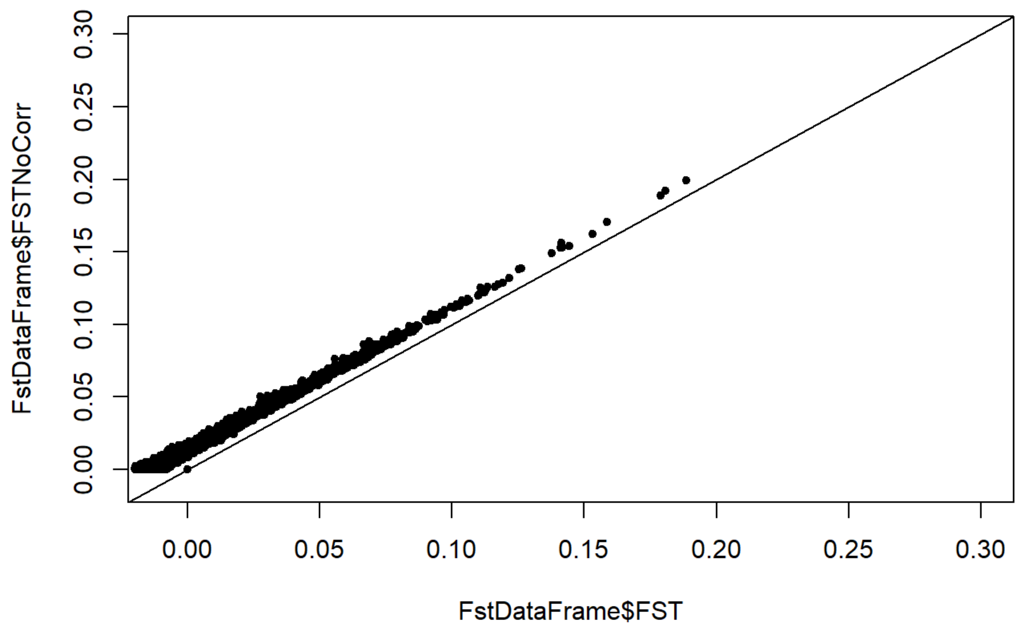


```
## integer(0)
```

```
hist(FstDataFrame$FSTNoCorr)
```

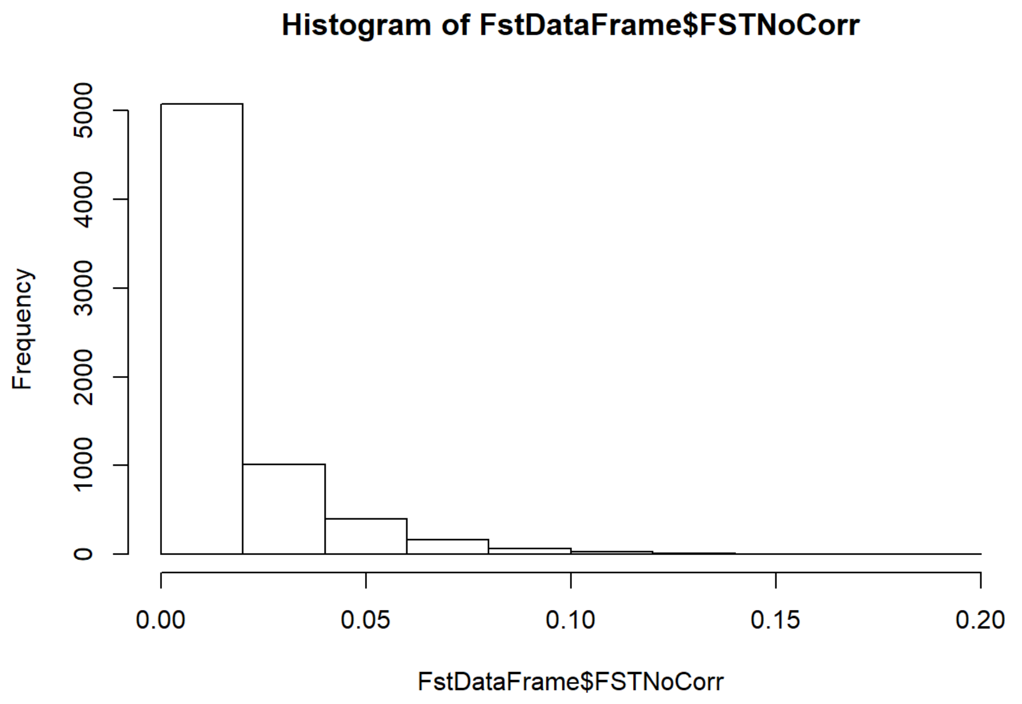


```
Outliers <- OutFLANK(FstDataFrame, NumberOfSamples = length(levels(gl@pop)),
    qthreshold = 0.01, LeftTrimFraction = 0.01, RightTrimFraction = 0.01,
    Hmin = 0.001)

OutFLANKResultsPlotter(Outliers, withOutliers = TRUE, NoCorr = TRUE,
    Hmin = 0.1, binwidth = 0.005, Zoom = FALSE, RightZoomFraction = 0.05,
    titletext = NULL)
```

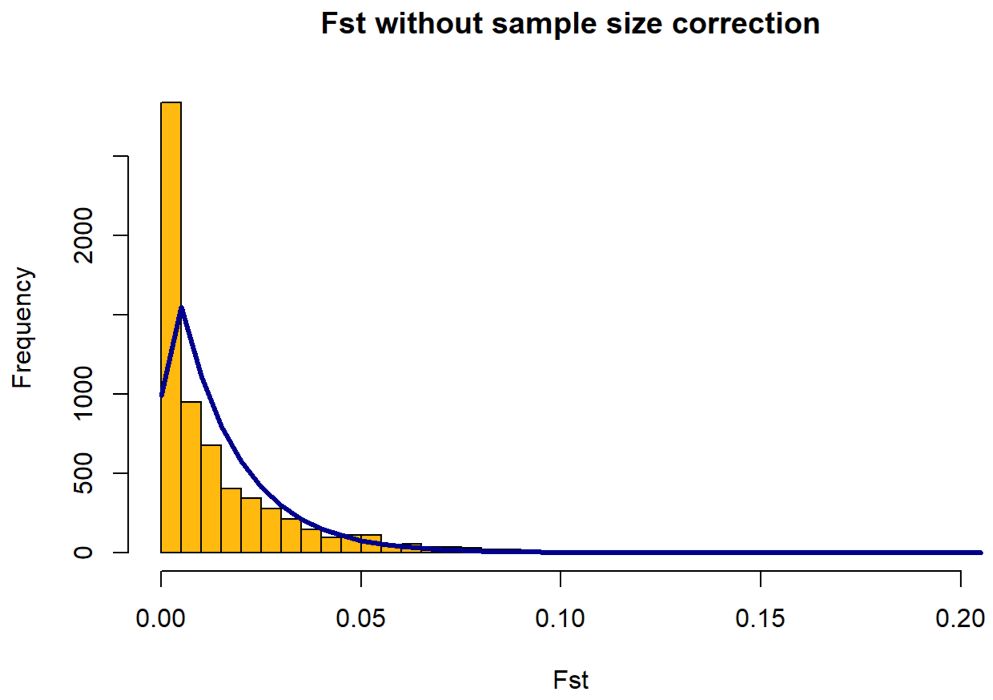


```
Outliers$numberLowFstOutliers
```

```
## [1] 0
```

```
Outliers$numberHighFstOutliers
```

```
## [1] 3
```

```
OutflankNames <- Outliers$results$LocusName[Outliers$results$OutlierFlag ==
    TRUE]
print(OutflankNames)
```

```
## [1] 12858443-53-T/G 12874190-22-C/T 12863758-49-C/T
## 6763 Levels: 100026312-20-A/G ... 12875817-24-G/A
```

4.5.2 PCadapt

```
data <- read.pcadapt("SS_ADMIXTURE-PCadapt.ped", type = "ped")  #samples in columns and Loci in Rows
```

```
## Summary:
##
##  - input file:               SS_ADMIXTURE-PCadapt.ped
##  - output file:
C:\Users\fdevloo\AppData\Local\Temp\RtmpGeJbwp\file2e1c1b443688.pcadapt
##
##  - number of individuals detected:   87
##  - number of loci detected:      6763
##
## 6763 lines detected.
## 87 columns detected.
```

```r
popdata <- read.table("SS_ADMIXTURE-PCadapt.ped")
poplist <- popdata[, 1]

K <- 25
x <- pcadapt(data, K = K, min.maf = 0.05)
x$singular.values
```

```
##  [1] 0.1682868 0.1653029 0.1613231 0.1414614 0.1392615
##  [6] 0.1384362 0.1364192 0.1237626 0.1228226 0.1217008
## [11] 0.1214592 0.1210575 0.1205781 0.1202810 0.1201297
## [16] 0.1198622 0.1195052 0.1192141 0.1190172 0.1185460
## [21] 0.1182754 0.1177375 0.1174259 0.1173063 0.1168911
```

```r
plot(x, option = "screeplot")  #K = 8
```

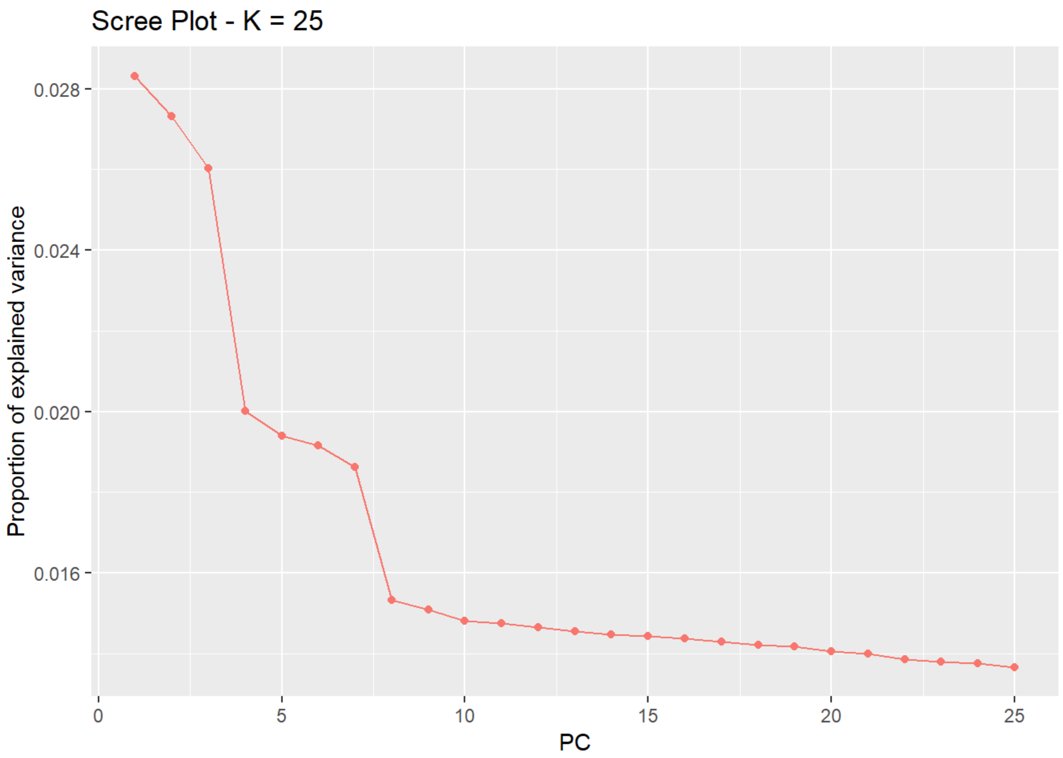


```r
plot(x, option = "scores", i = 1, j = 2, pop = poplist)
```

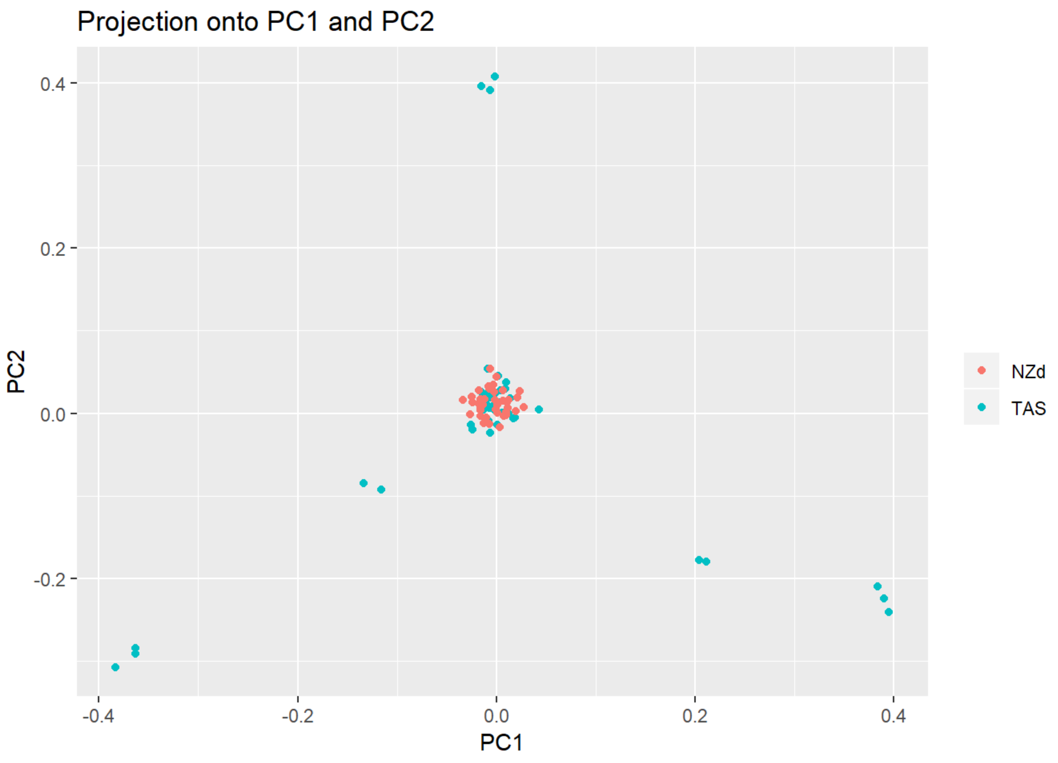


```
plot(x, option = "scores", i = 1, j = 3, pop = poplist)
```

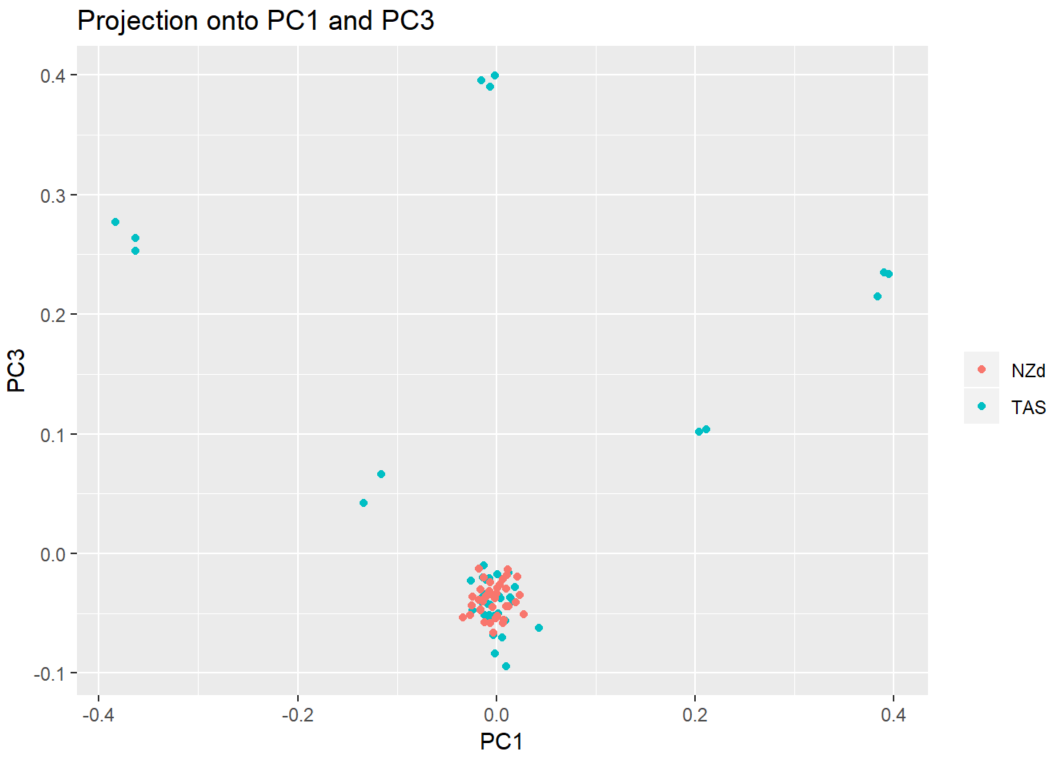


```
plot(x, option = "scores", i = 2, j = 3, pop = poplist)
```

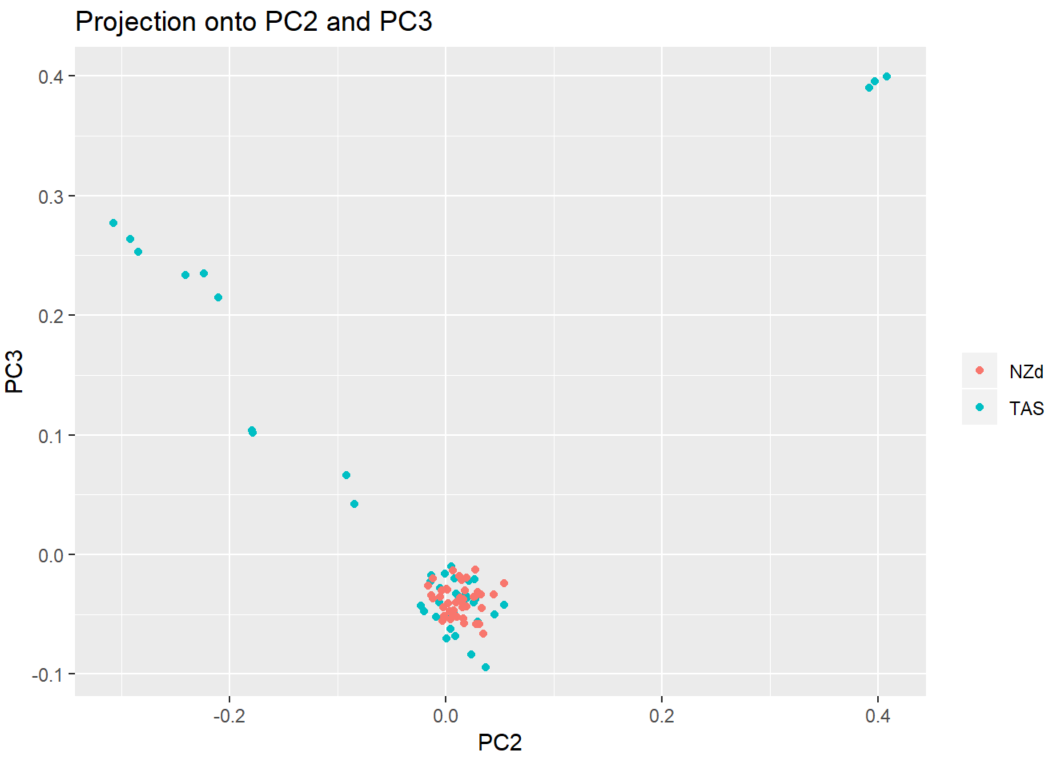


```
plot(x, option = "scores", i = 3, j = 4, pop = poplist)
```

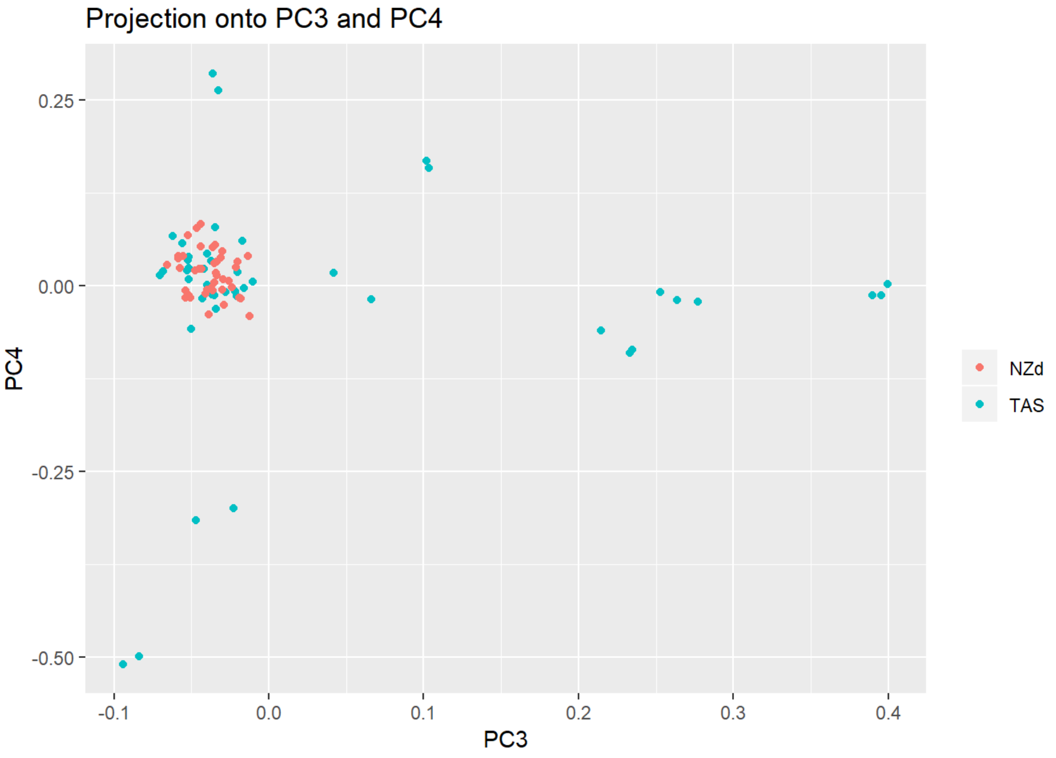


```
K <- 8
x <- pcadapt(data, K = K, min.maf = 0.05)
x$singular.values
```

```
## [1] 0.1682868 0.1653029 0.1613231 0.1414614 0.1392614
## [6] 0.1384358 0.1364192 0.1237625
```

```
plot(x, option = "manhattan")
```

Manhattan Plot

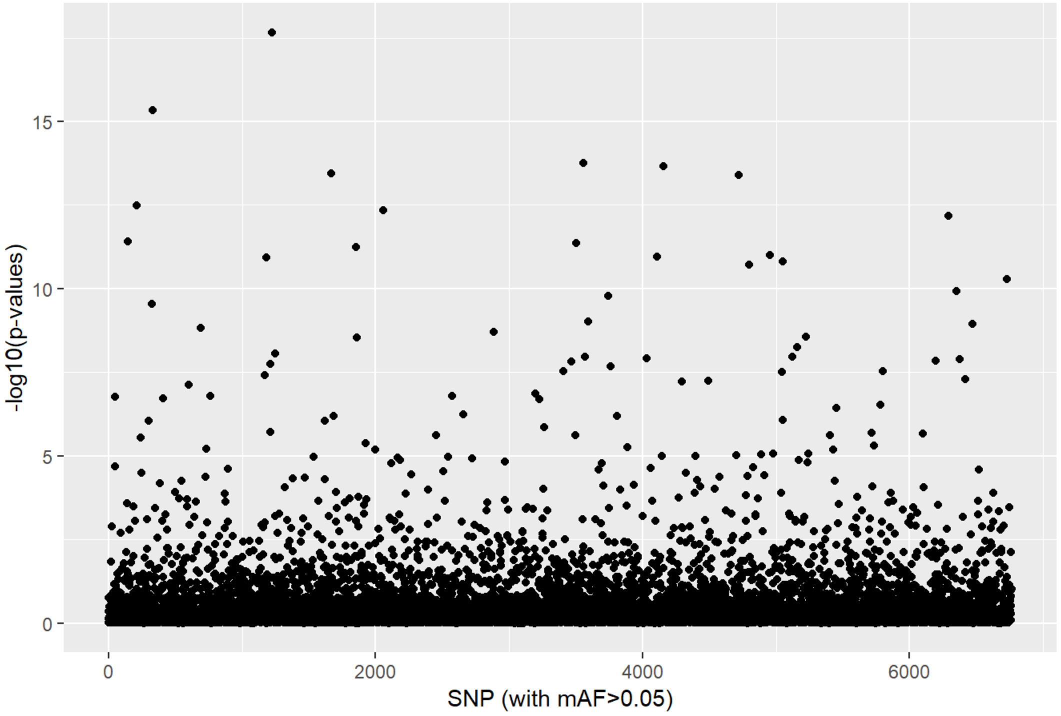


```
plot(x, option = "qqplot", threshold = 0.05)
```

Q-Q plot

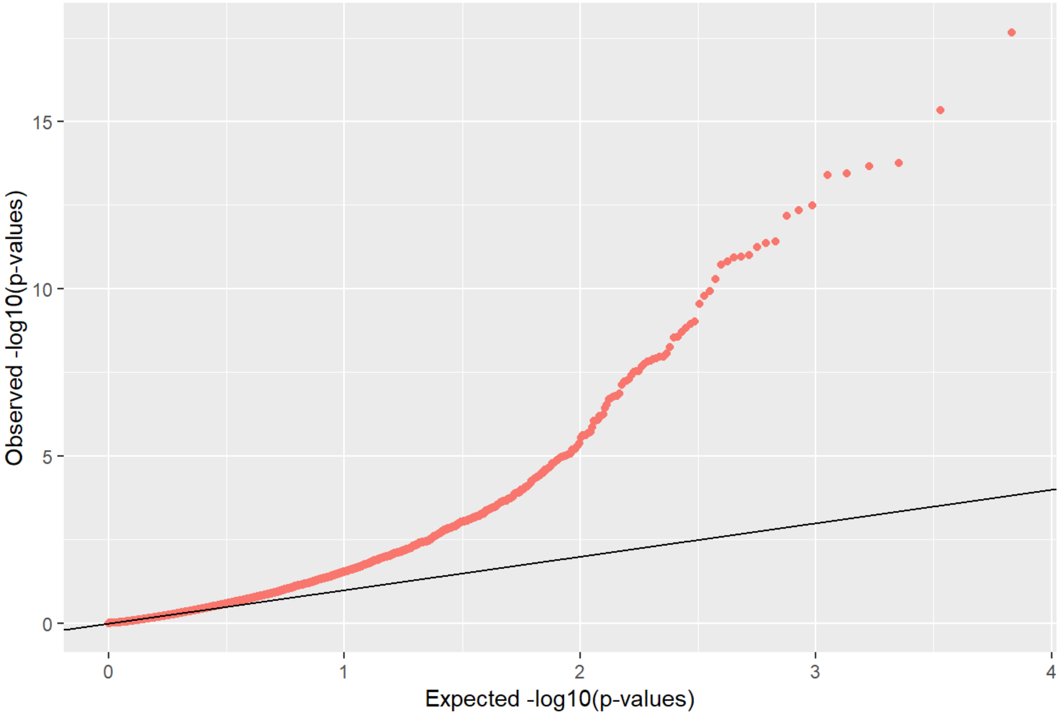


```
hist(x$pvalues, xlab = "p-values", main = NULL, breaks = 50)
```

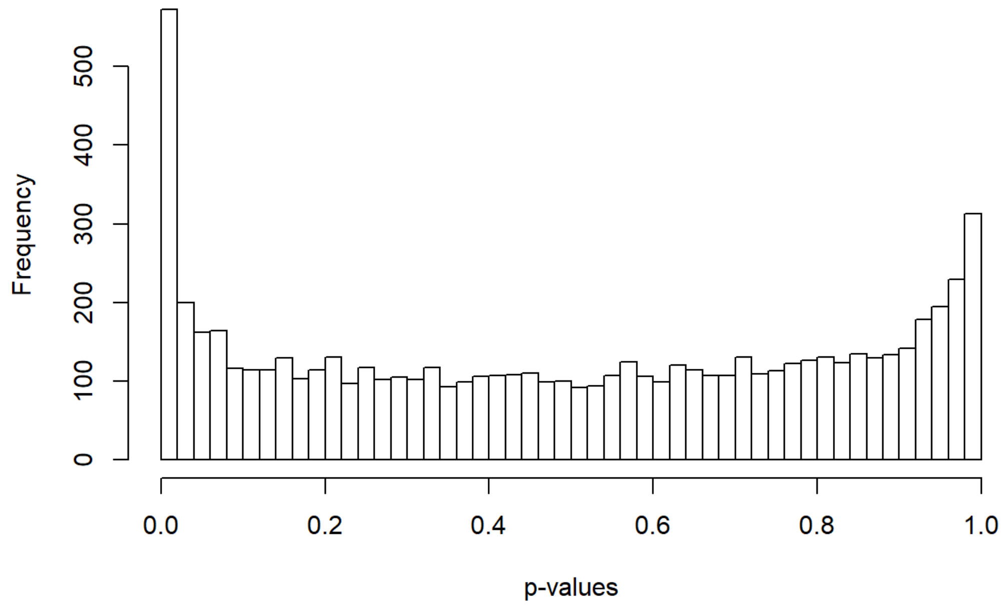


```
plot(x, option = "stat.distribution")
```

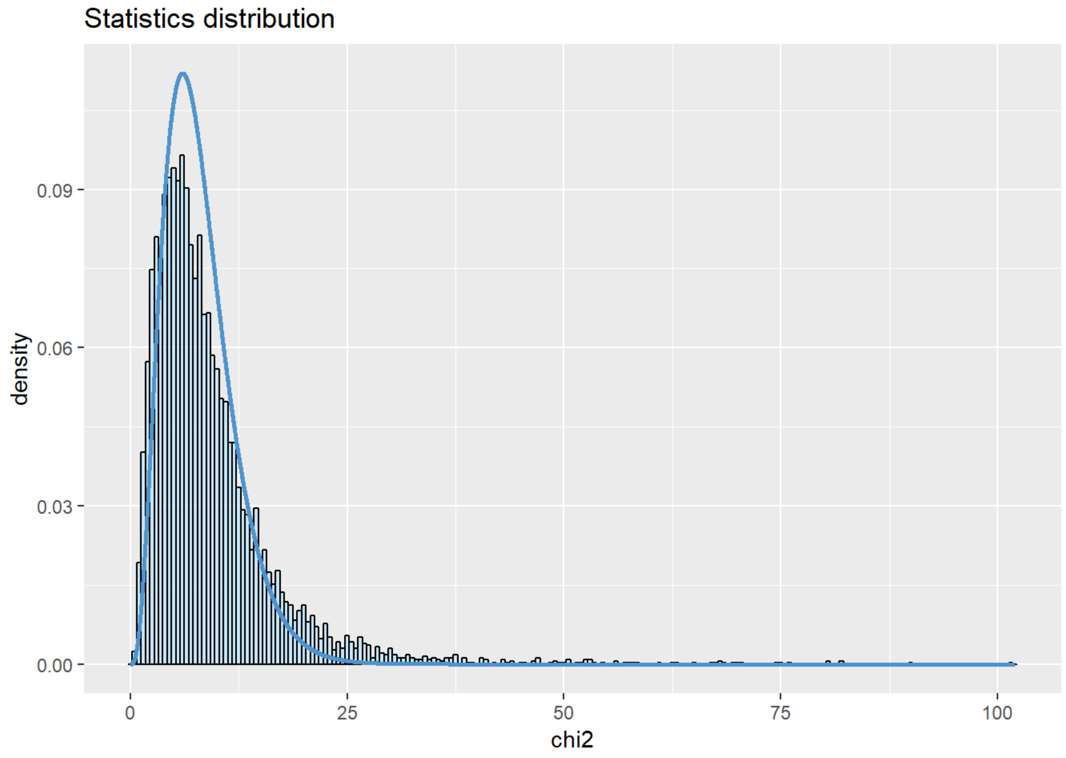


```
qval <- qvalue(x$pvalues)$qvalues
alpha <- 0.01
pcadapt <- which(qval < alpha)
length(pcadapt)
```

```
## [1] 137
```

```
PCadaptkNames <- gl$loc.names[pcadapt]
print(PCadaptkNames)
```

```
##   [1] "12867420-10-A/G"  "12867741-21-C/T"
##   [3] "12870507-14-T/A"  "12873713-31-C/T"
```

```
##   [3] "12870507-14-T/A"  "12875715-31-C/T"
##   [5] "12869508-10-G/A"  "12875683-30-T/C"
##   [7] "12866050-22-C/T"  "12874505-27-G/A"
##   [9] "12869317-40-C/T"  "12872819-32-G/A"
##  [11] "12857944-52-T/C"  "100085690-10-C/A"
##  [13] "12860165-20-C/G"  "12873445-26-C/A"
##  [15] "12868721-14-A/C"  "12869676-24-T/C"
##  [17] "12869693-17-G/T"  "12872378-12-G/A"
##  [19] "12869277-33-T/C"  "12872185-35-T/A"
##  [21] "12869450-44-C/G"  "12873754-10-C/T"
##  [23] "12861307-35-G/A"  "12857998-40-A/C"
##  [25] "12866206-51-A/G"  "12863493-20-G/T"
##  [27] "12871843-21-G/A"  "12868400-41-T/C"
##  [29] "12859346-47-C/T"  "12874700-26-C/T"
##  [31] "12865874-38-G/A"  "12867634-48-A/T"
##  [33] "12866529-30-G/A"  "12872057-22-G/A"
##  [35] "12875169-5-C/T"   "12871236-21-G/A"
##  [37] "12866860-40-C/T"  "12859742-45-A/C"
##  [39] "12867338-49-A/C"  "12863202-15-C/T"
##  [41] "12874191-7-T/C"   "12863264-44-A/G"
##  [43] "12873572-7-A/G"   "12859439-6-T/C"
##  [45] "12867811-39-A/G"  "12870615-61-C/T"
##  [47] "12870193-33-A/G"  "12871948-12-A/G"
##  [49] "12872025-23-T/C"  "12858204-56-A/G"
##  [51] "12861147-9-G/A"   "12870143-27-G/T"
##  [53] "12864323-64-G/A"  "12860766-29-T/C"
##  [55] "12864395-37-G/A"  "12864347-21-A/C"
##  [57] "12869795-56-T/G"  "12869091-12-A/C"
##  [59] "12871462-20-T/C"  "12874274-43-A/G"
##  [61] "12873543-8-A/C"   "12871216-12-G/C"
##  [63] "12860374-21-G/A"  "12857717-51-G/A"
##  [65] "12865665-56-G/C"  "12861775-14-T/C"
##  [67] "12857654-31-T/C"  "12867078-47-C/G"
##  [69] "100051940-21-C/T" "12861048-33-A/T"
##  [71] "12875495-42-G/A"  "12873199-36-C/T"
##  [73] "12867148-27-G/A"  "12857580-7-C/T"
##  [75] "12869607-57-G/A"  "12867343-28-A/G"
##  [77] "12867260-31-G/A"  "12873567-5-C/A"
##  [79] "12868268-40-G/A"  "12864783-44-A/G"
##  [81] "12868871-42-A/C"  "12870690-32-C/T"
##  [83] "12862576-15-T/C"  "12872213-13-G/A"
##  [85] "12860832-7-G/A"   "12867626-36-G/A"
##  [87] "12868151-6-T/G"   "12860594-12-A/G"
##  [89] "100058533-15-T/G" "12862695-44-A/C"
##  [91] "12869965-36-G/C"  "12874887-45-G/A"
##  [93] "12867245-18-T/C"  "12867122-26-C/T"
##  [95] "12862423-38-C/G"  "12857423-32-G/A"
##  [97] "12864906-42-T/C"  "12869599-44-G/A"
##  [99] "12868862-22-T/G"  "12859744-51-G/A"
## [101] "12870423-12-A/C"  "12868800-21-T/C"
## [103] "12871519-11-C/G"  "12865171-20-A/T"
## [105] "12863713-43-G/A"  "100106995-53-C/T"
## [107] "12862889-13-T/C"  "12869682-41-T/C"
## [109] "12870953-28-T/G"  "12864215-40-A/T"
## [111] "12867193-28-C/T"  "12866731-13-G/T"
## [113] "12865986-51-G/A"  "12863955-66-G/C"
## [115] "100127413-27-T/A" "12858212-67-A/G"
## [117] "12873803-19-G/C"  "12860508-26-G/C"
## [119] "12865209-23-T/A"  "12867244-7-C/T"
## [121] "12861296-27-T/C"  "12866372-64-G/A"
## [123] "12864718-16-T/G"  "12871724-39-A/G"
## [125] "12868136-5-G/A"   "12869083-12-T/C"
## [127] "12859453-9-A/C"   "12860985-15-C/G"
## [129] "12861569-36-C/T"  "100052522-29-A/G"
## [131] "12862183-36-G/A"  "12863005-35-A/G"
## [133] "12863278-13-G/A"  "12859846-45-C/G"
```

```
## [135] "12858372-54-A/G"  "12857810-49-G/A"
## [137] "12859099-39-G/A"
```

4.5.3 Remove outliers

First we check if there is any overlap between the two methods for detecting outliers.
Since OutFLANK is more stringent, we chose to only remove outliers based on OutFLANK

```
PCadaptkNames %in% OutflankNames
```

```
##   [1] FALSE FALSE FALSE FALSE FALSE FALSE FALSE FALSE FALSE
##  [10] FALSE FALSE FALSE FALSE FALSE FALSE FALSE FALSE FALSE
##  [19] FALSE FALSE FALSE FALSE FALSE FALSE FALSE FALSE FALSE
##  [28] FALSE FALSE FALSE FALSE FALSE FALSE FALSE FALSE FALSE
##  [37] FALSE FALSE FALSE FALSE FALSE FALSE FALSE FALSE FALSE
##  [46] FALSE FALSE FALSE FALSE FALSE FALSE FALSE FALSE FALSE
##  [55] FALSE FALSE FALSE FALSE FALSE FALSE FALSE FALSE FALSE
##  [64] FALSE FALSE FALSE FALSE FALSE FALSE FALSE FALSE FALSE
##  [73] FALSE FALSE FALSE FALSE FALSE FALSE FALSE FALSE FALSE
##  [82] FALSE FALSE FALSE FALSE FALSE FALSE FALSE FALSE FALSE
##  [91] FALSE FALSE FALSE FALSE FALSE FALSE FALSE FALSE FALSE
## [100] FALSE FALSE FALSE FALSE FALSE FALSE FALSE FALSE FALSE
## [109] FALSE FALSE FALSE FALSE FALSE FALSE FALSE FALSE FALSE
## [118] FALSE FALSE FALSE FALSE FALSE FALSE FALSE FALSE FALSE
## [127] FALSE FALSE FALSE FALSE FALSE FALSE FALSE FALSE FALSE
## [136] FALSE FALSE
```

```
OutflankNames %in% PCadaptkNames
```

```
## [1] FALSE FALSE FALSE
```

```
rm <- gl$loc.names %in% OutflankNames
gl <- gl[, !rm]
gl$other$loc.metrics <- gl$other$loc.metrics[!rm, ]
```

4.6 Export data to other software formats

```
gi <- gl2gi_mvb(gl)
save(gl, gi, file = "gl-gi_with.rdata")
# load('gl-gi_with.rdata')
gl2gpop(gl, filename = "SS_genepop.txt")
gl2Adm(gl, filename = "SS_ADMIXTURE-PCadapt")
dartR::gl2structure(gl, outfile = "SS_STRUCTURE.txt", outpath = getwd())
```

```
## Structure file saved as: SS_STRUCTURE.txt
## in folder: C:/Users/fdevloo/OneDrive - University of Tasmania/PhD/3A.School shark/R-analysis
```

4.7 Genetic diversity

```
set.seed(124)
bastat <- diveRsity::basicStats(infile = "SS_genepop.txt", outfile = "SS_BasicStat_output.txt",
    fis_ci = T, ar_ci = T, fis_boots = boots, ar_boots = boots,
    mc_reps = boots, rarefaction = F, ar_alpha = 0.05, fis_alpha = 0.05)

gendiv <- data.frame(Tasmania = bastat$main_tab$SsTAS_Bx02_A01$overall,
    `New Zealand` = bastat$main_tab$SsNZd_Bx03_A01$overall, Combined =
rowMeans(cbind(bastat$main_tab$SsTAS_Bx02_A01$overall,
        bastat$main_tab$SsNZd_Bx03_A01$overall)))
row.names(gendiv) <- rownames(bastat$main_tab$SsTAS_Bx02_A01)
knitr::kable(gendiv, caption = "Genetic diversity with full siblings")
```

Genetic diversity with full siblings

	Tasmania	New.Zealand	Combined
ar	1.994	1.994	1.9940
size	45.760	40.787	43.2735
obs_het	0.264	0.262	0.2630
exp_het	0.284	0.283	0.2835
uexp_het	0.287	0.286	0.2865
fis	0.069	0.068	0.0685
hwe_glb	0.000	1.000	0.5000
hwe_hom	1.000	1.000	1.0000
hwe_het	0.000	0.002	0.0010
fis_lo	0.050	0.047	0.0485
fis_hi	0.063	0.061	0.0620
ar_lo	1.989	1.989	1.9890
ar_hi	1.998	1.997	1.9975

4.8 Fixation and differentiation indices

4.8.1 Global

```
set.seed(124)
pwfst <- StAMPP::stamppFst(gl, nboots = boots, percent = 95,
    nclusters = 3)
Fst <- c(pwfst$Bootstraps$Fst, pwfst$Bootstraps$`p-value`, pwfst$Bootstraps$`Lower bound CI limit`,
    pwfst$Bootstraps$`Upper bound CI limit`)
names(Fst) <- c("Fst", "P-value", "Lower CI", "Upper CI")
print(Fst)
```

```
##         Fst     P-value    Lower CI    Upper CI
## 0.002265426 0.000000000 0.001733970 0.002825730
```

```
bs <- mmod::chao_bootstrap(gi, nreps = boots2)
bs.D <- mmod::summarise_bootstrap(bs, D_Jost)
```

```
## Warning in mmod::summarise_bootstrap(bs, D_Jost): Bootstrap
## distribution of D_Jost includes negative values, harmonic
## mean is undefined
```

```
print(bs.D$summary.global.het)
```

```
##          observed      lower.normal      upper.normal
##      0.0013504272      0.0010430104      0.0016578439
##           std.dev              mean  lower.percentile
##      0.0001568453      0.0060622305      0.0058128908
## upper.precentile
##      0.0063873981
```

```
nc.diff_stats <- mmod::diff_stats(gi, phi_st = TRUE)
print(nc.diff_stats$global)
```

```
##          Hs          Ht     Gst_est   Gprime_st       D_het
## 0.294856191 0.295332314 0.001612159 0.004565208 0.001350427
##      D_mean      Phi_st
##          NA 0.006487541
```

```
with(nc.diff_stats, pairs(per.locus[, 3:6], upper.panel = panel.smooth))
```

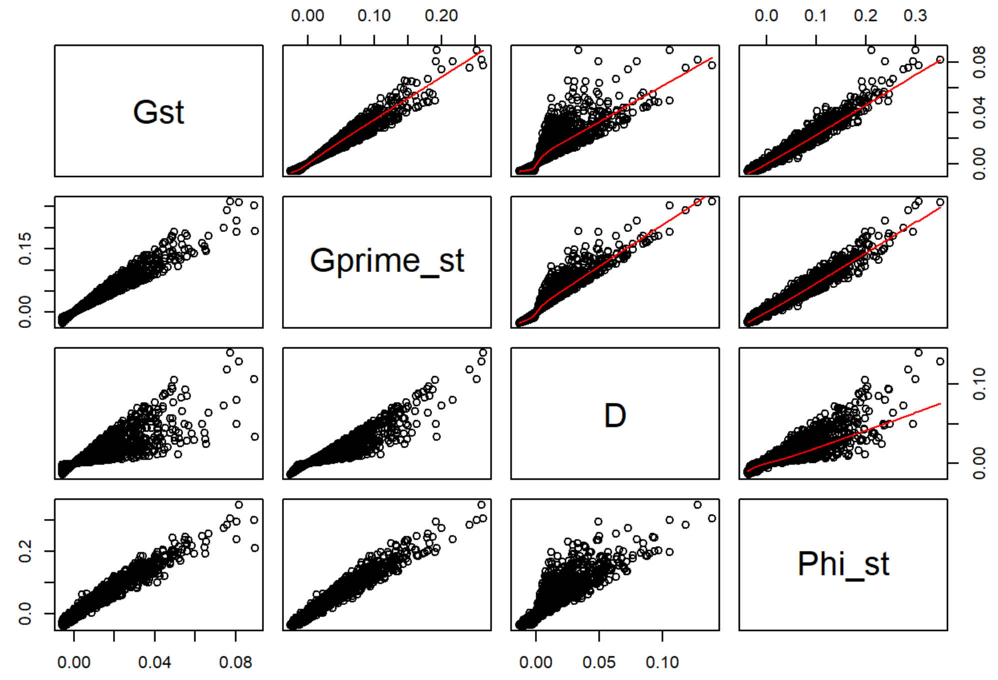

4.8.2 Pairwise

```
set.seed(124)
knitr::kable(rbind(pwfst$Fsts, pwfst$Pvalues), digits = 5, caption = " Weir and Cockerham's Fst (abo
ve) and p-values (below)")
```

Weir and Cockerham's Fst (above) and p-values (below)

	TAS	NZd
TAS	NA	NA
NZd	0.00227	NA
TAS	NA	NA
NZd	0.00000	NA

```
PD <- as.matrix(mmod::pairwise_D(gi, linearized = FALSE, hsht_mean = "arithmetic"))
knitr::kable(PD, digits = 5, caption = "Jost D - arithmetic mean")
```

Jost D - arithmetic mean

	TAS	NZd
TAS	0.00000	0.00135
NZd	0.00135	0.00000

4.9 DAPC

4.9.1 DAPC with location prior

4.9.1.1 Cross-validation to identify the optimal number of PC's

Uses a training - holdout set of individuals to check how reliable individuals can be assigned.

```
x <- gl
set.seed(124)
xval <- adegenet::xvalDapc(tab(x, NA.method = "mean"), pop(x),
    n.pca.max = nInd(x), training.set = 0.9, result = "groupMean",
    center = TRUE, scale = FALSE, n.rep = boots, xval.plot = TRUE)
```

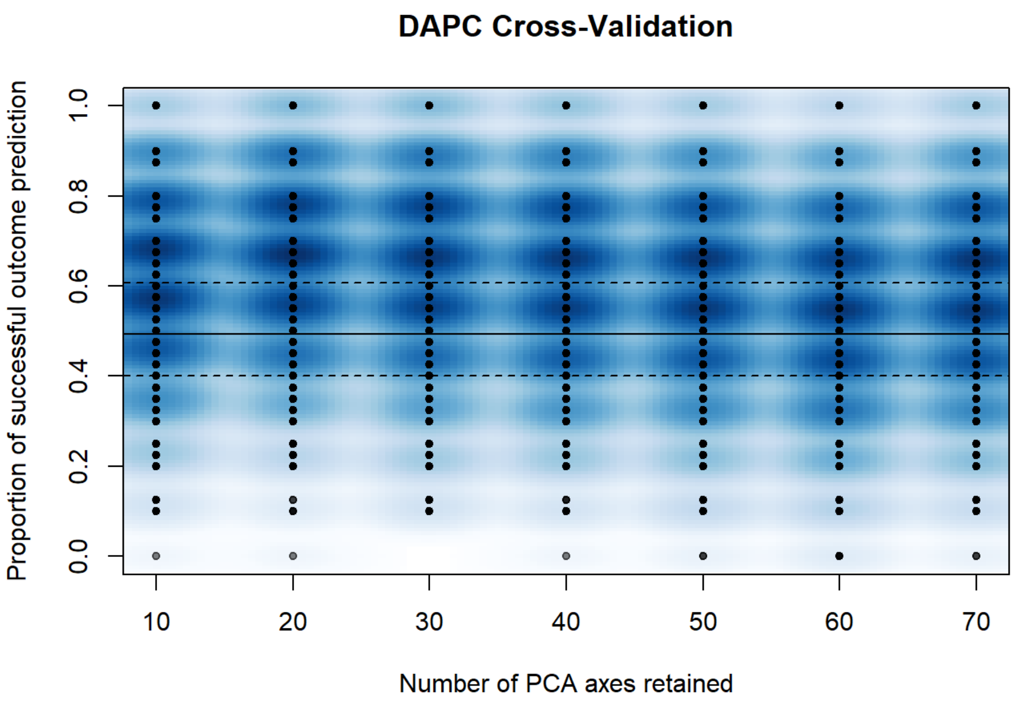


```
print(xval[2:6])
PC <- as.numeric(xval$`Number of PCs Achieving Lowest MSE`)

# refine the analysis
xval <- adegenet::xvalDapc(tab(x, NA.method = "mean"), pop(x),
    n.pca = (PC - 15):(PC + 15), n.rep = boots, parallel = "multicore",
    ncpus = 3L, xval.plot = TRUE)
```

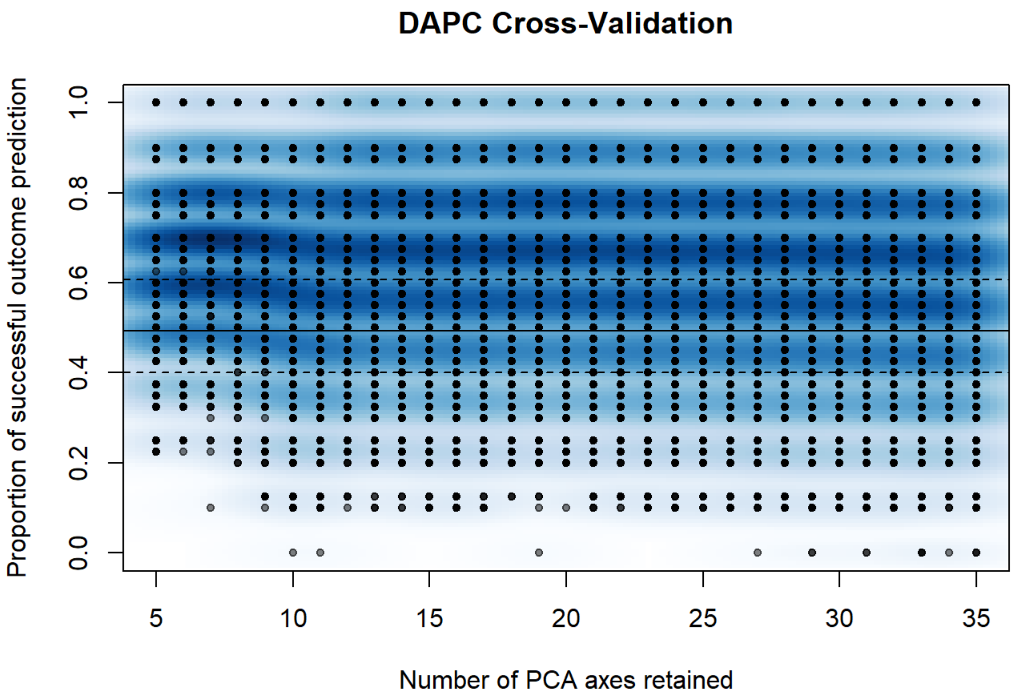


```
print(xval[2:6])
PC <- as.numeric(xval$`Number of PCs Achieving Lowest MSE`)
```

```
## $`Median and Confidence Interval for Random Chance`
##      2.5%       50%     97.5%
## 0.4003181 0.4925769 0.6079003
##
## $`Mean Successful Assignment by Number of PCs of PCA`
##        10        20        30        40        50        60
## 0.6164650 0.6572650 0.6397725 0.6184550 0.5958700 0.5557375
##        70
## 0.5886325
##
## $`Number of PCs Achieving Highest Mean Success`
## [1] "20"
##
## $`Root Mean Squared Error by Number of PCs of PCA`
##        10        20        30        40        50        60
## 0.4117528 0.3731916 0.3926314 0.4125832 0.4334683 0.4719115
##        70
## 0.4400413
##
## $`Number of PCs Achieving Lowest MSE`
## [1] "20"
##
## $`Median and Confidence Interval for Random Chance`
##      2.5%       50%     97.5%
## 0.4003181 0.4925769 0.6079003
##
## $`Mean Successful Assignment by Number of PCs of PCA`
##         5         6         7         8         9        10
## 0.6406250 0.6453500 0.6825550 0.6513875 0.6300975 0.6179200
##        11        12        13        14        15        16
## 0.6021275 0.6570900 0.6668875 0.6583175 0.6501425 0.6449000
##        17        18        19        20        21        22
## 0.6321800 0.6728925 0.6637275 0.6597725 0.6621175 0.6486675
##        23        24        25        26        27        28
## 0.6456750 0.6443350 0.6447600 0.6484725 0.6405400 0.6330200
##        29        30        31        32        33        34
## 0.6217200 0.6409075 0.6286000 0.6260175 0.6145025 0.6076250
##        35
## 0.6156375
##
## $`Number of PCs Achieving Highest Mean Success`
## [1] "7"
##
## $`Root Mean Squared Error by Number of PCs of PCA`
##         5         6         7         8         9        10
## 0.3771654 0.3757468 0.3366859 0.3706826 0.3927037 0.4098943
##        11        12        13        14        15        16
## 0.4259489 0.3729139 0.3646912 0.3733953 0.3804825 0.3856569
##        17        18        19        20        21        22
## 0.3976974 0.3581437 0.3668141 0.3709324 0.3704685 0.3830489
##        23        24        25        26        27        28
## 0.3858055 0.3868060 0.3868078 0.3845061 0.3928344 0.3995652
##        29        30        31        32        33        34
## 0.4096413 0.3909176 0.4024163 0.4058518 0.4163872 0.4213378
##        35
## 0.4138989
##
## $`Number of PCs Achieving Lowest MSE`
## [1] "7"
```

4.9.1.2 Barplot

Group individuals according to DAPC posterior membership.
carefull: the strong priors can sometimes assign random noise to the predefined structure

```
dapc <- xval$DAPC
colour <- funky(2)
post <- as.data.frame(dapc$posterior)
barplot(t(as.matrix(post)), col = colour, xlab = "Individual",
    ylab = "Assignment", main = paste0("Location prior: K=2 & PC=",
        PC, sep = ""), border = NA, ylim = c(0, 1.2), names.arg = row.names(post),
    las = 2, cex.names = 0.5, cex.lab = 1.5, cex.axis = 1.5)
```

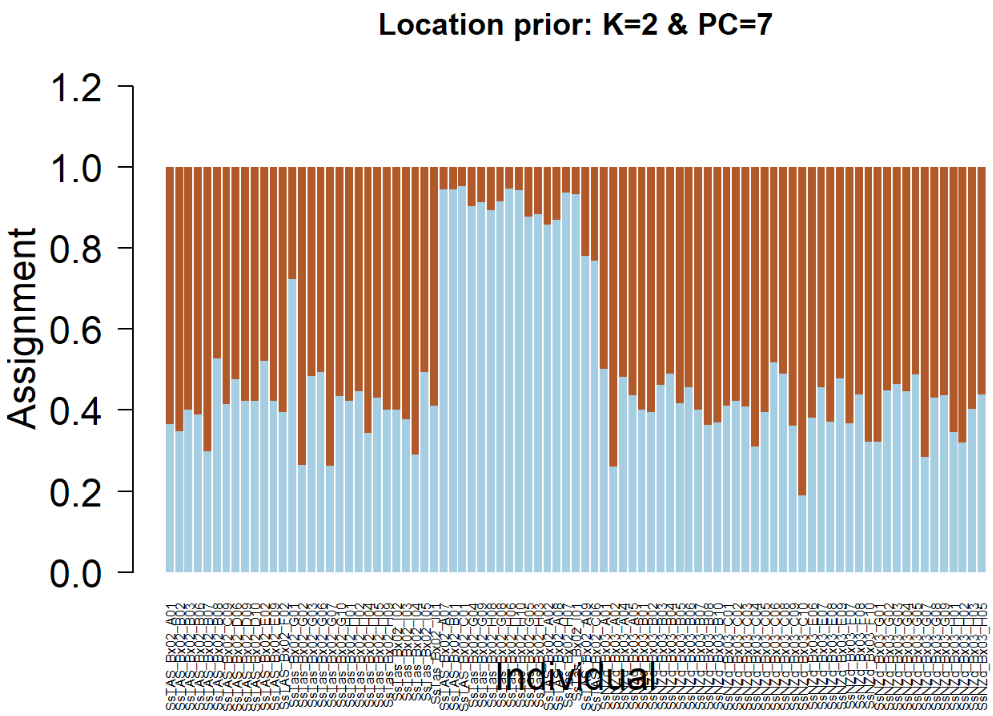

4.9.2 DAPC without location prior

The find.clusters function will give a PRIOR assignment according to the data to run DAPC.

4.9.2.1 Optimal number of clusters

```
# set.seed(124) grp <- adegenet::find.clusters(gi,
# max.n.clust = 20, n.pca = nInd(gl)/3, stat='BIC') save(grp,
# file = 'Neo_K8_DAPC2.rdata')
load("Neo_K8_DAPC2.rdata")

y <- as.numeric(grp$Kstat)
x <- 1:35
data <- data.frame(x, y, stringsAsFactors = F)
plot <- ggplot2::ggplot(data, aes(x, y)) + geom_point(size = 5,
    shape = 1, color = "blue") + geom_line(size = 1, color = "blue") +
    scale_x_continuous(name = waiver(), breaks = seq(from = 0,
        to = nrow(gl) - 1, by = 5)) + labs(subtitle = "", y = "Bayesian Information Criterion",
    x = "Number of clusters", title = "", caption = "") + theme_classic() +
    theme(axis.text = element_text(size = 15), axis.title.x = element_text(size = 20,
        vjust = 0, hjust = 0.5), axis.title.y = element_text(size = 20,
        vjust = 2, hjust = 0.5))
print(plot)
```

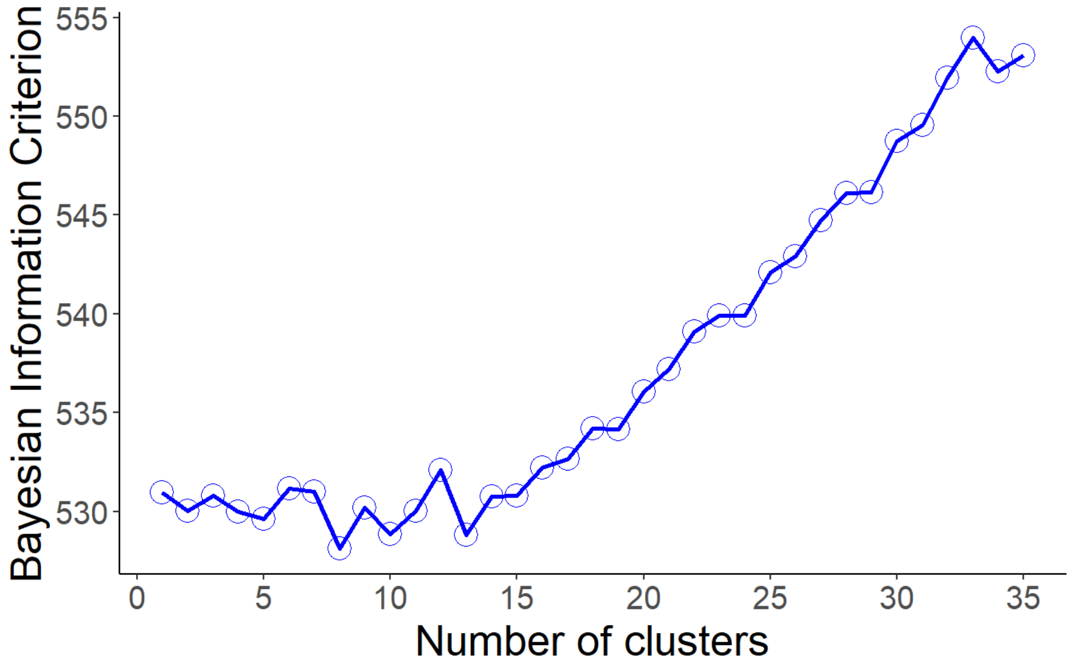

4.9.2.2 Barplot

Group individuals according to DAPC posterior membership.

```
for (K in 2:9) {
    set.seed(124)
    grp <- adegenet::find.clusters(gl, n.clust = K, n.pca = PC)
    set.seed(124)
    dapc <- adegenet::dapc(gl, grp$grp, n.da = K - 1, n.pca = 5)
    colour <- funky(K)
    post <- as.data.frame(dapc$posterior)
    colnames(post) <- paste("Group", 1:nlevels(dapc$grp))
    barplot(t(as.matrix(post)), col = colour, xlab = "", ylab = "Assignment",
        main = paste0("K=", K, " & PC=", PC, sep = ""), border = NA,
        ylim = c(0, 1.2), names.arg = row.names(post), las = 2,
        cex.names = 0.5, cex.lab = 1.5, cex.axis = 1.5)
}
```

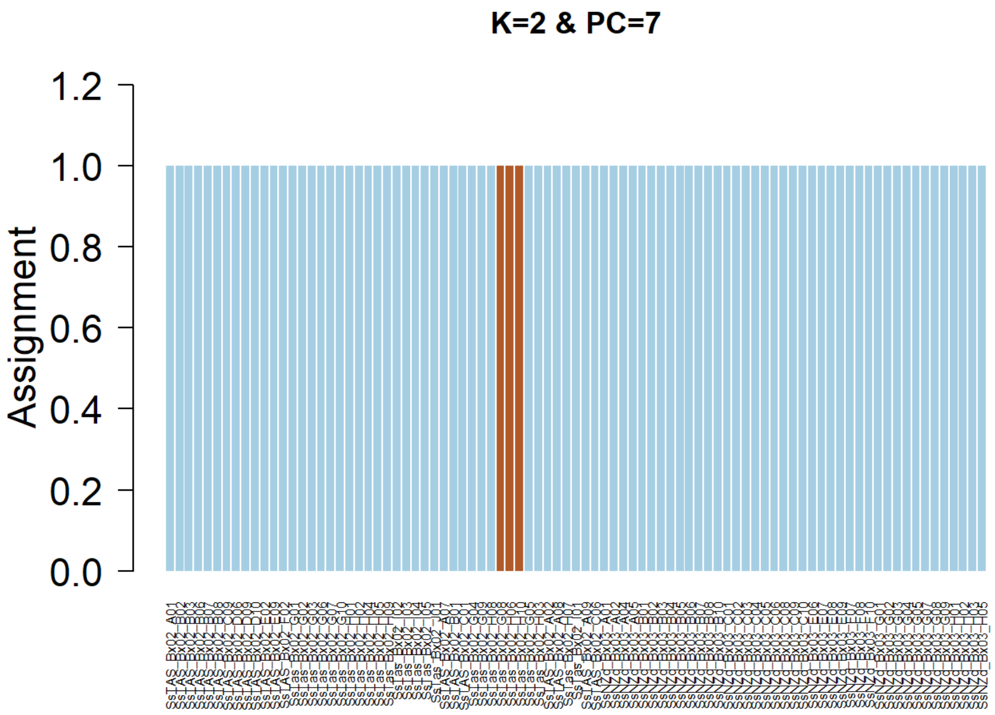
K=2 & PC=7
Assignment
1.2
1.0
0.8
0.6
0.4
0.2
0.0

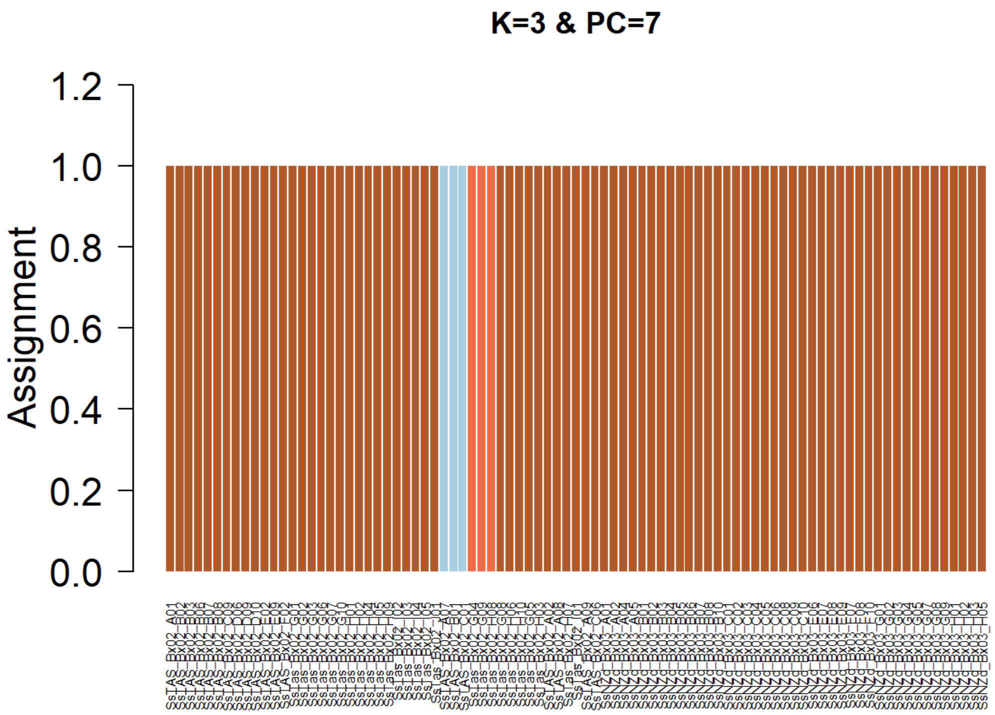
K=3 & PC=7
Assignment
1.2
1.0
0.8
0.6
0.4
0.2
0.0

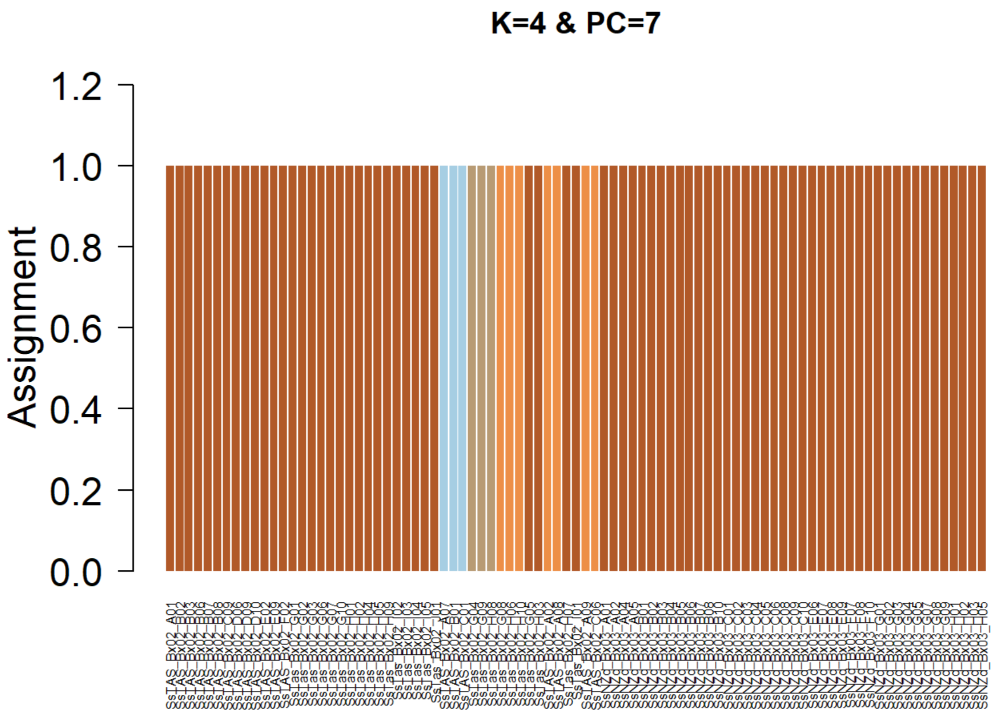
K=4 & PC=7
Assignment
1.2
1.0
0.8
0.6
0.4
0.2
0.0

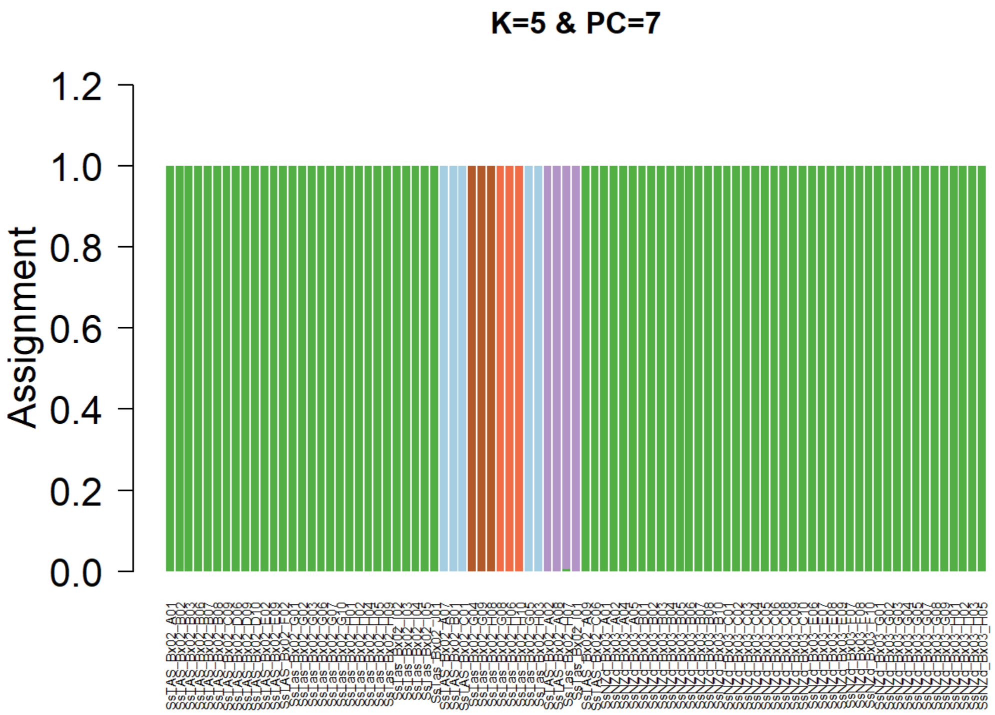
K=5 & PC=7
Assignment
1.2
1.0
0.8
0.6
0.4
0.2
0.0

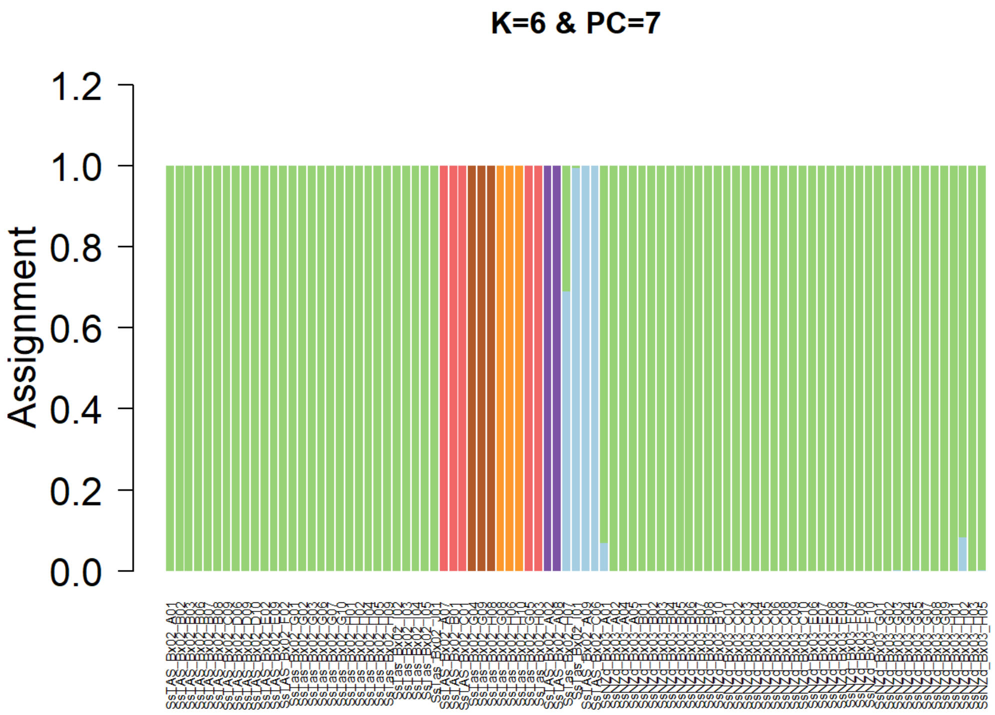
K=6 & PC=7
Assignment
1.2
1.0
0.8
0.6
0.4
0.2
0.0

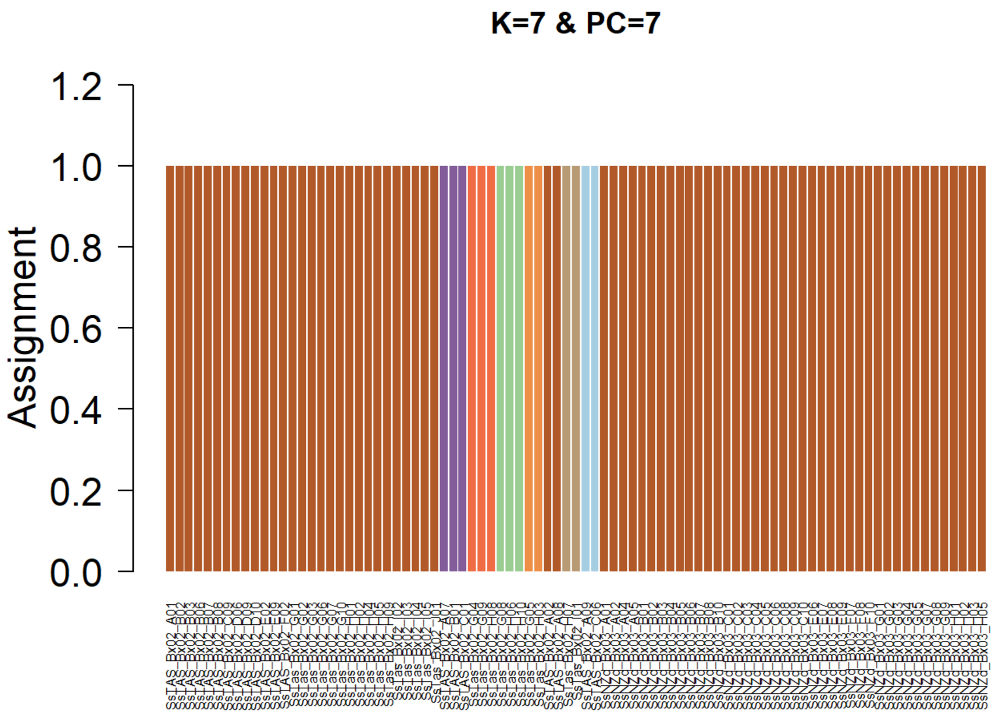
K=7 & PC=7
Assignment
1.2
1.0
0.8
0.6
0.4
0.2
0.0

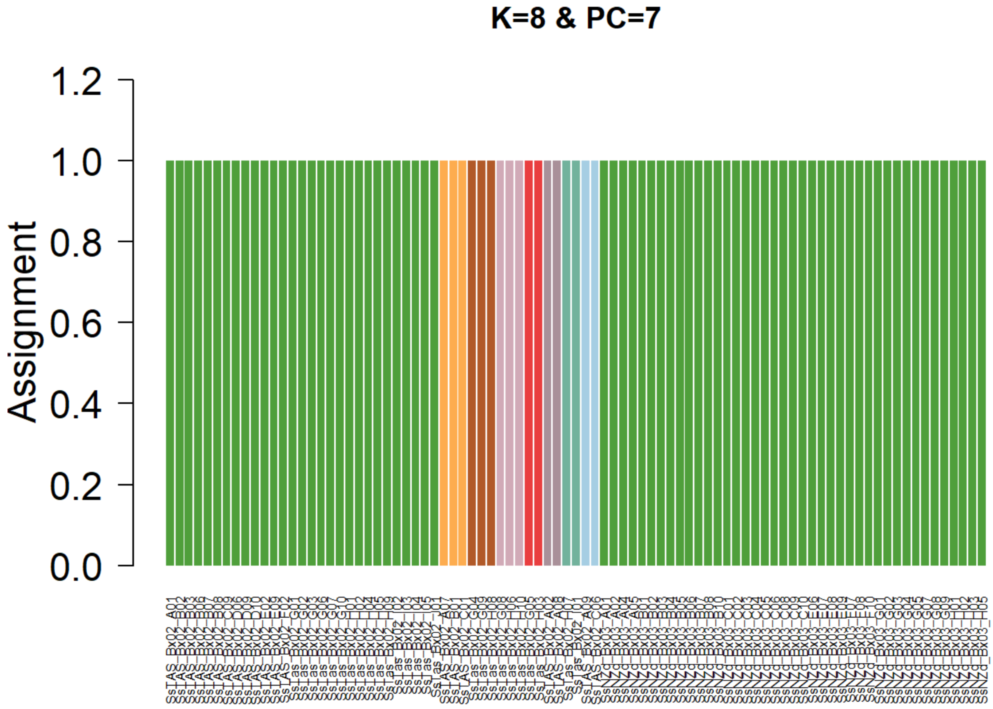

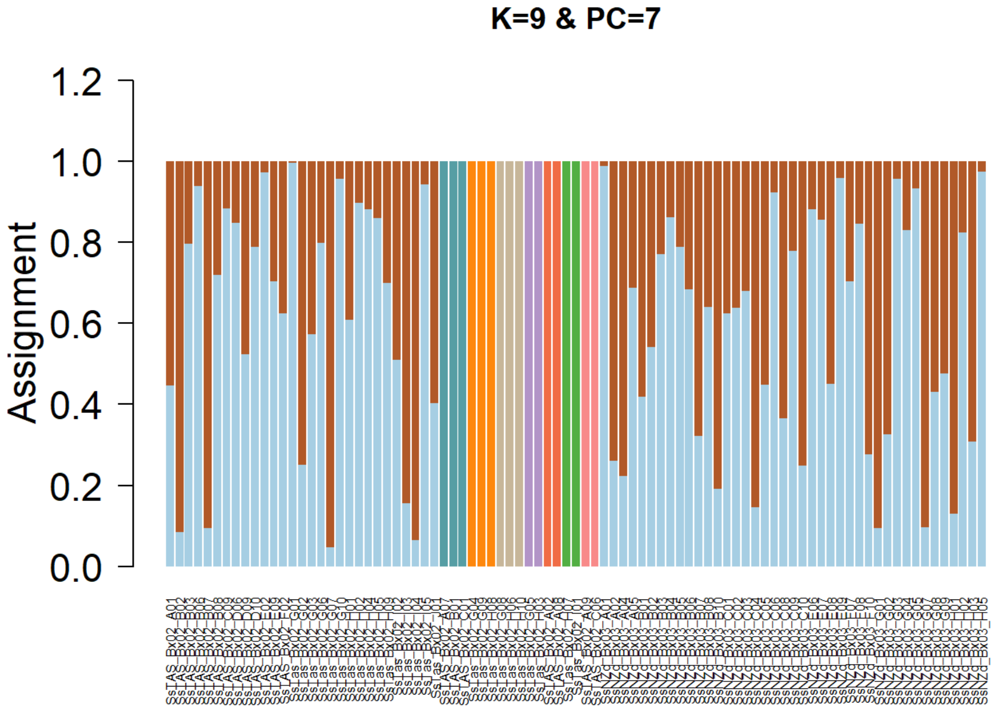

4.10 STRUCTURE output

```r
for (k in 2:9) {
    colour <- funky(k)
    tbl <- read.table(paste0("./STRUCTURE/Neo2/K", k, "/K", k,
        "outfile1_q", sep = ""))
    for (n in 2:20) {
        tmp <- read.table(paste0("./STRUCTURE/Neo2/K", k, "/K",
            k, "outfile", n, "_q", sep = ""))
        tmp2 <- tmp[, -c(1, 2)]
        tbl2 <- tbl[, -c(1, 2)]
        out <- cbind(tmp2, tbl2)
        Means <- sapply(unique(colnames(out)), function(i) rowMeans(out[,
            colnames(out) == i]))
        tbl <- data.frame(V1 = tbl$V1, V2 = tbl$V2, Means)
    }
    names <- tbl$V1
    tbl$V1 <- NULL
    tbl$V2 <- NULL

    suppressWarnings(barplot(t(as.matrix(tbl)), col = colour,
        xlab = "", ylab = "Ancestry", main = paste0("K=", k,
            sep = ""), border = NA, ylim = c(0, 1.2), names.arg = names,
        las = 2, cex.names = 0.5, cex.lab = 1.5, cex.axis = 1.5))
}
```

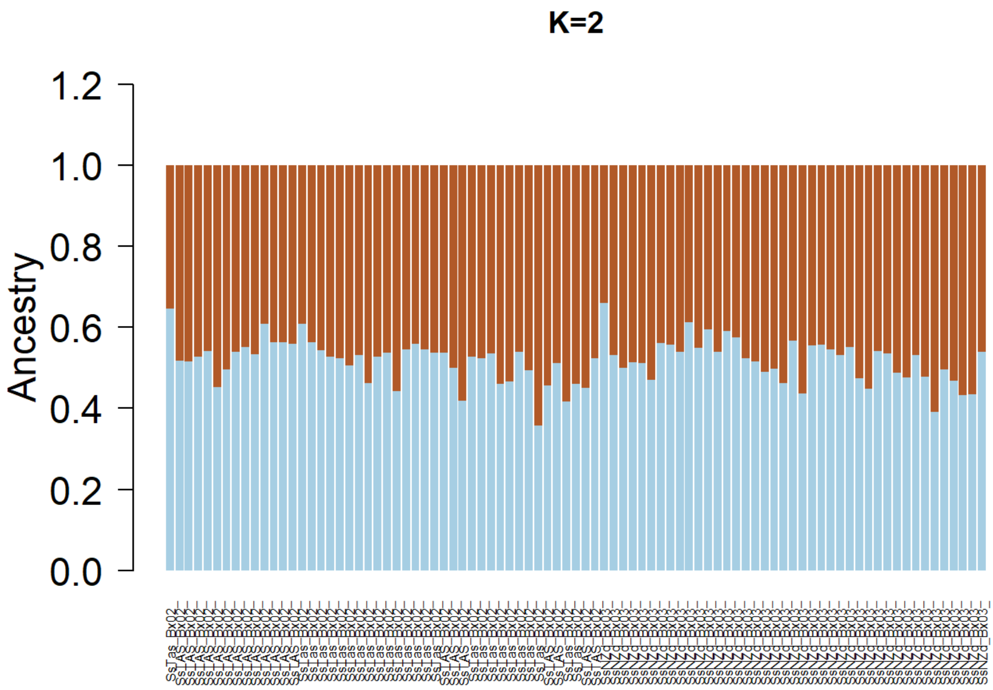

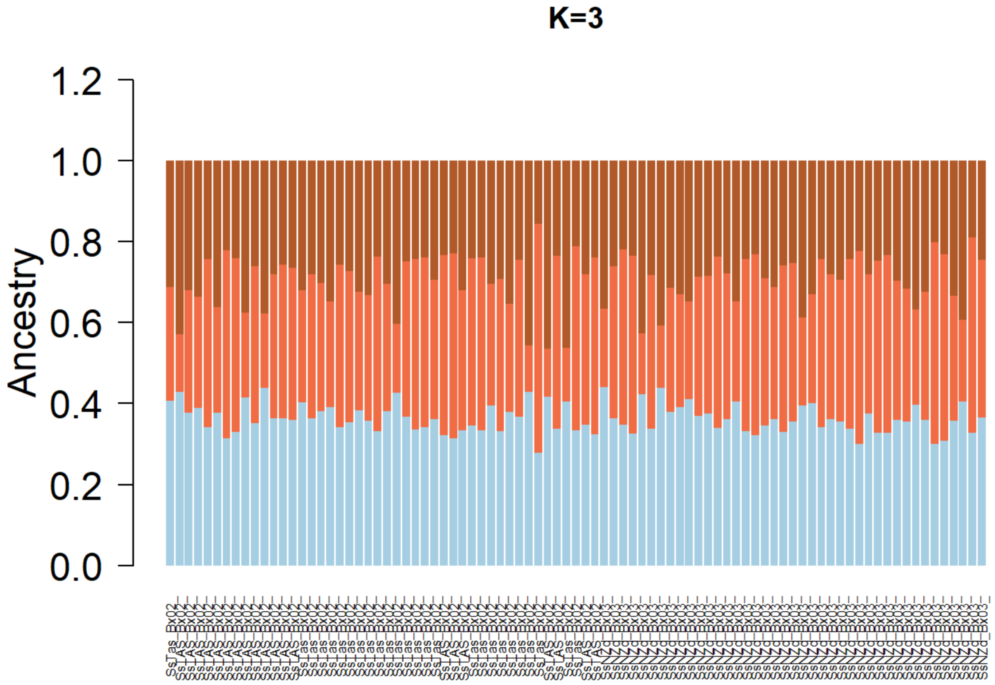
K=3
Ancestry
1.2
1.0
0.8
0.6
0.4
0.2
0.0

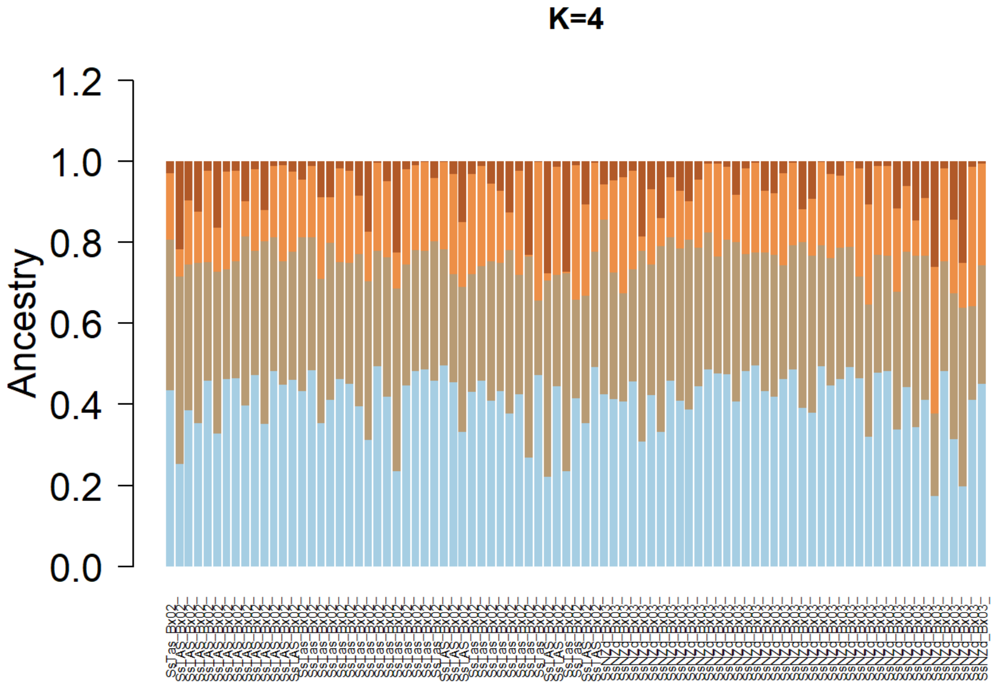
K=4
Ancestry
1.2
1.0
0.8
0.6
0.4
0.2
0.0

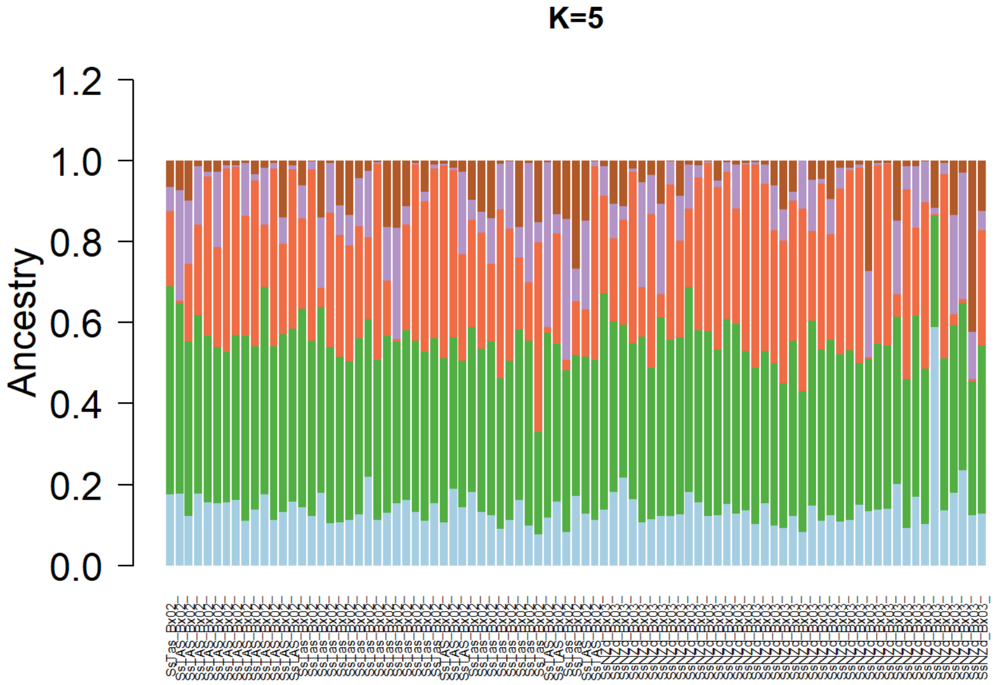
K=5
Ancestry
0.0
0.2
0.4
0.6
0.8
1.0
1.2

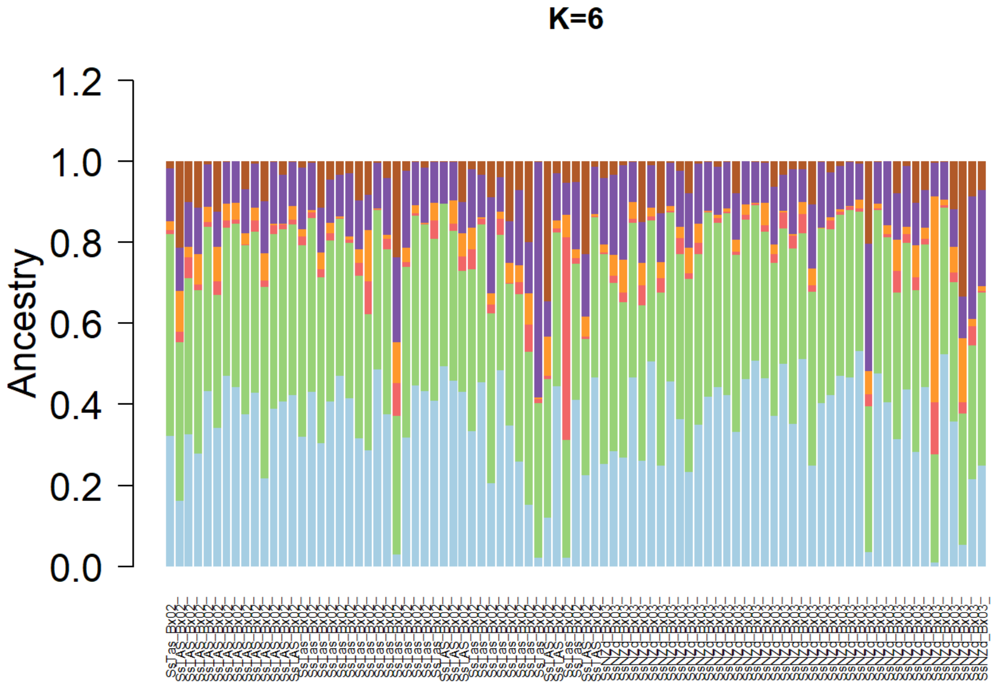
K=6
Ancestry
0.0
0.2
0.4
0.6
0.8
1.0
1.2

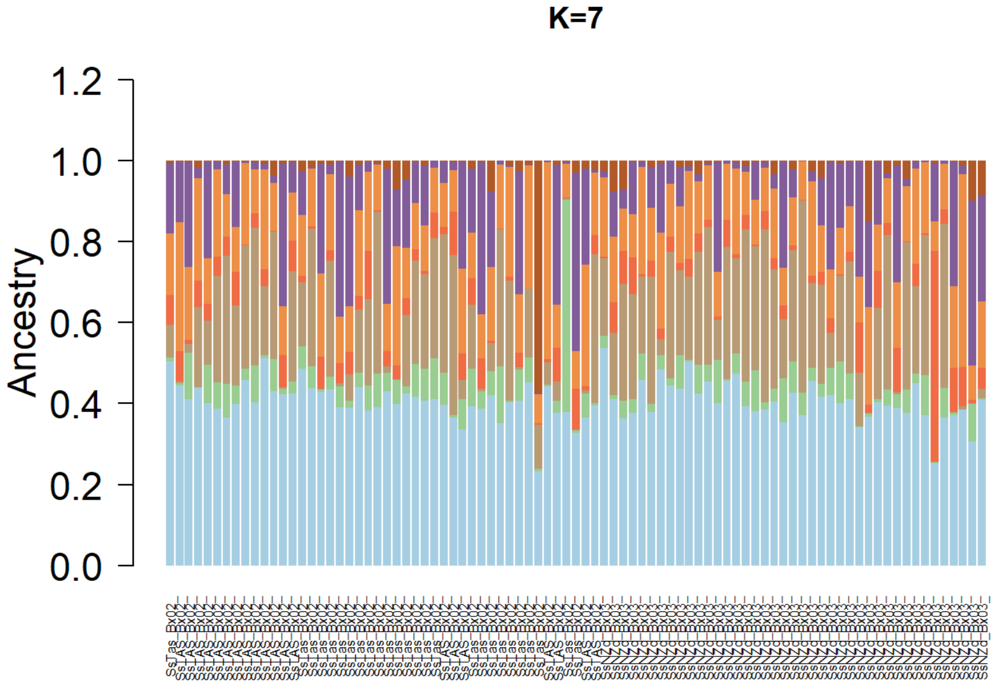
K=7
Ancestry
1.2
1.0
0.8
0.6
0.4
0.2
0.0

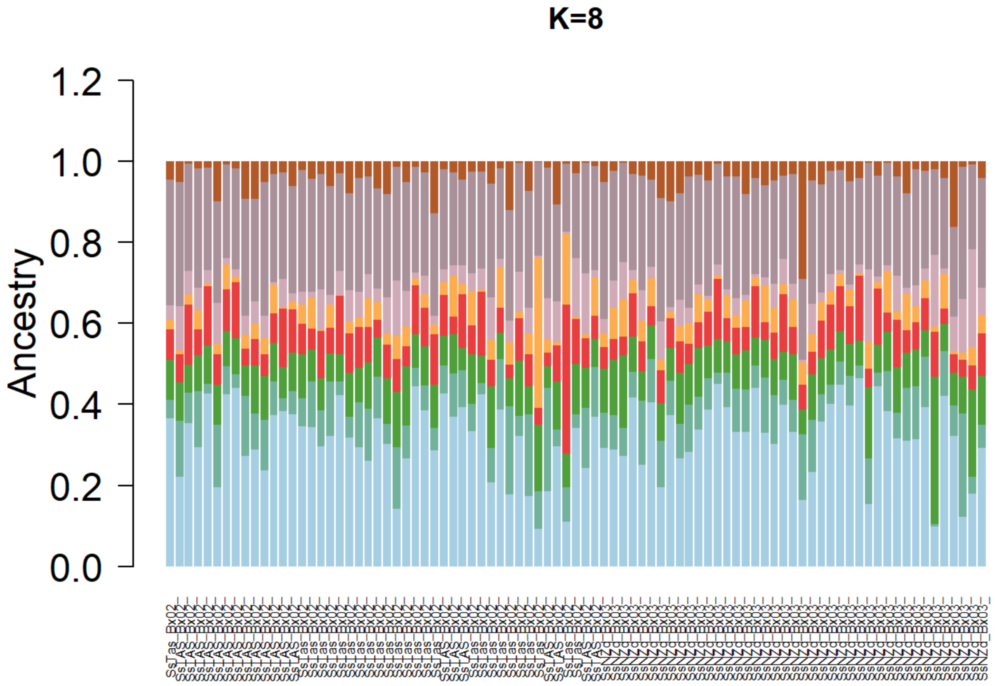
K=8
Ancestry
1.2
1.0
0.8
0.6
0.4
0.2
0.0

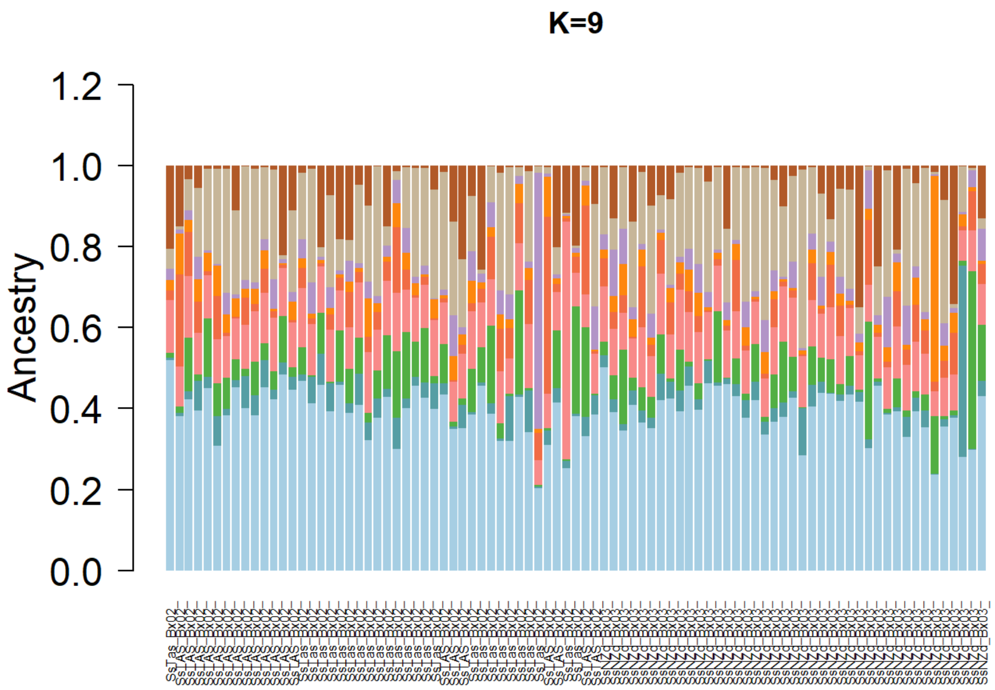

5 Second analysis: Without full-sibs

5.1 Load data

```
dartfile <- "DGale15_1833_1863_SNP_CallRate_0.1-Edited2.csv"
meta <- "schoolshark_meta2.csv"
lastmetric <- "TotalPicRepSnpTest"

gl <- dartR::gl.read.dart(filename = dartfile, covfilename = meta,
    topskip = 0, lastmetric = lastmetric, probar = F)
```

```
## Trying to determine if one row or two row format...
## Found  1  row(s) format. Proceed...
## Added the following covmetrics:
## AlleleID CloneID ClusterTempIndex AlleleSequence ClusterConsensusSequence ClusterSize
AlleleSeqDist SNP SnpPosition CallRate OneRatioRef OneRatioSnp FreqHomRef FreqHomSnp FreqHets PICRef
PICSnp AvgPIC AvgCountRef AvgCountSnp SumCount RatioAvgCountRefAvgCountSnp FreqHetsMinusFreqMinHom A
lleleCountsCorrelation aggregateTagsTotal DerivedCorrMinusSeedCorr RepRef RepSNP RepAvg PicRepRef Pi
cRepSNP TotalPicRepRefTest TotalPicRepSnpTest .
## Number of rows per Clone. Should be only  1 s: 1
##  Recognised: 88 individuals and 31550  SNPs in a 1 row format using
DGale15_1833_1863_SNP_CallRate_0.1-Edited2.csv
## Start conversion....
## Format is 1 rows.
## Please note conversion of bigger data sets will take some time!
## Once finished, we recommend to save the object using save(object, file="object.rdata")
## Try to add covariate file: schoolshark_meta2.csv .
## Ids of covariate file does not match the number of ids in the genetic file. Maybe this is fine if
a subset matches.
## Ids of covariate file (at least a subset of) are matching!
## Found  88 matching ids out of 723 ids provided in the covariate file. Subsetting snps now!.
##  Added pop factor.
## Please note:there is no lat column
## Please note:there is no lon column
## Added  id  to the other$ind.metrics slot.
##  Added  SpeciesName  to the other$ind.metrics slot.
##  Added  pop  to the other$ind.metrics slot.
##  Added  Sex  to the other$ind.metrics slot.
##  Added  Collection.Location  to the other$ind.metrics slot.
##  Added  State  to the other$ind.metrics slot.
##  Added  CollectionContact  to the other$ind.metrics slot.
##  Added  DateCollected  to the other$ind.metrics slot.
##  Added  Date  to the other$ind.metrics slot.
##  Added  Day.Collected  to the other$ind.metrics slot.
##  Added  Month.Collected  to the other$ind.metrics slot.
##  Added  Year_collected  to the other$ind.metrics slot.
##  Added  DateArrivedCSIRO  to the other$ind.metrics slot.
##  Added  Day.Arrived.CSIRO  to the other$ind.metrics slot.
##  Added  Month.Arrived.CSIRO  to the other$ind.metrics slot.
##  Added  Year.Arrived.CSIRO  to the other$ind.metrics slot.
##  Added  VesselName  to the other$ind.metrics slot.
##  Added  Log.Book.Number  to the other$ind.metrics slot.
##  Added  Page.number  to the other$ind.metrics slot.
##  Added  Length_TOT_cm  to the other$ind.metrics slot.
##  Added  Length_PAR_cm  to the other$ind.metrics slot.
##  Added  Length  to the other$ind.metrics slot.
##  Added  X  to the other$ind.metrics slot.
##  Added  FSP  to the other$ind.metrics slot.
##  Added  Sibs  to the other$ind.metrics slot.
##  Added  Siblings  to the other$ind.metrics slot.
##  Added  SexSiblings  to the other$ind.metrics slot.
##  Added  Tag.Number  to the other$ind.metrics slot.
##  Added  Sample.box.position..DNA.  to the other$ind.metrics slot.
##  Added  X.1  to the other$ind.metrics slot.
##  Added  DArT.Plate.number  to the other$ind.metrics slot.
##  Added  DTU.number  to the other$ind.metrics slot.
##  Added  DNAQuality  to the other$ind.metrics slot.
##  Added  Location.of.DNA.sample  to the other$ind.metrics slot.
##  Added  Location.of.tissue.sample  to the other$ind.metrics slot.
##  Added  Comments  to the other$ind.metrics slot.
##  Added  Sibs2  to the other$ind.metrics slot.
##  Added  Sex2  to the other$ind.metrics slot.
##  Added  yearcollect  to the other$ind.metrics slot.
##  Added  Age  to the other$ind.metrics slot.
##  Added  Cohort  to the other$ind.metrics slot.
```

```
pop.levels <- c("TAS", "NZd")
gl$pop <- factor(x = gl$pop, levels = pop.levels)
gl <- gl[order(gl$pop, gl$other$ind.metrics$FSP, gl$ind.names)]
```

5.2 Remove full siblings

Full siblings were identified with COLONY2

```
FSP <- gl$other$ind.metrics[grepl("FS*", gl$other$ind.metrics$FSP),
    ]
gl <- gl[!grepl("FS*", gl$other$ind.metrics$Sibs), ]
gl$other$ind.metrics <- gl$other$ind.metrics[!grepl("FS*", gl$other$ind.metrics$Sibs),
    ]
```

5.3 FSP summary

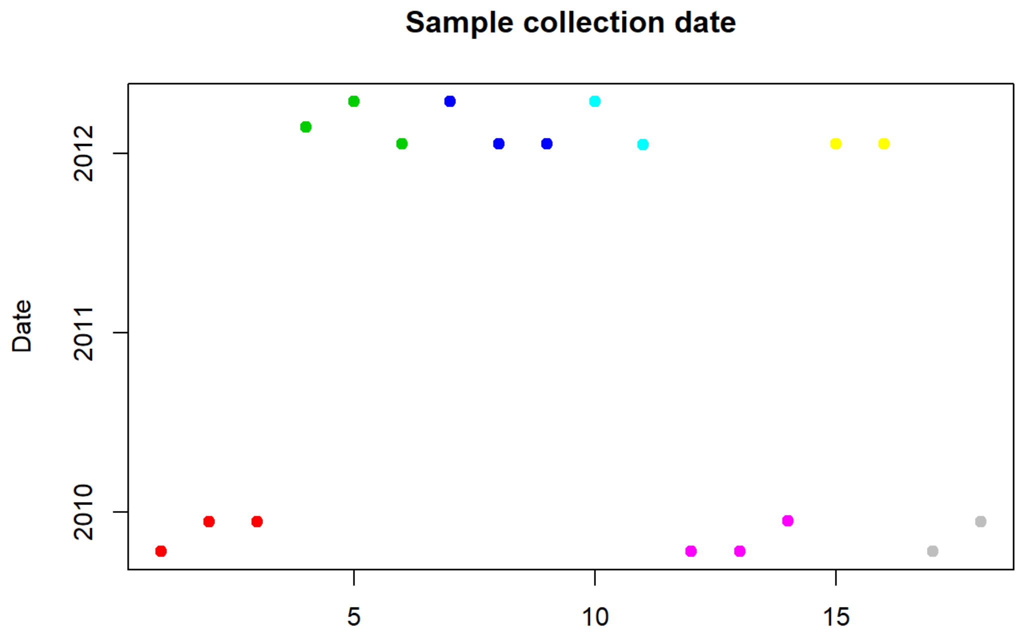

The points are coloured according to FS group

FSP Age in Tasmania, based on the length and growth curve from Francis et al. 1998

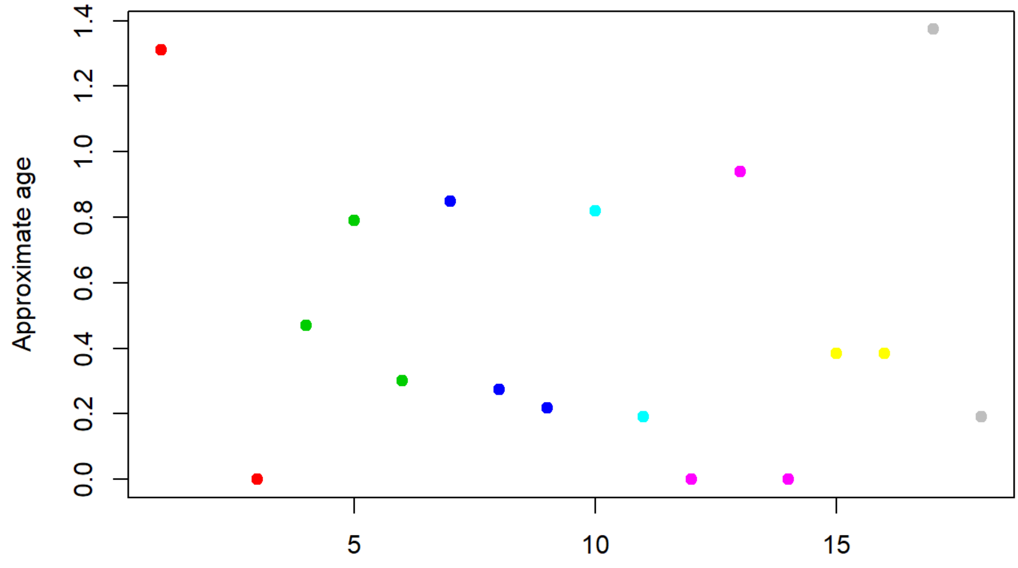

The points are coloured according to FS group

FSP Cohort year in Tasmania

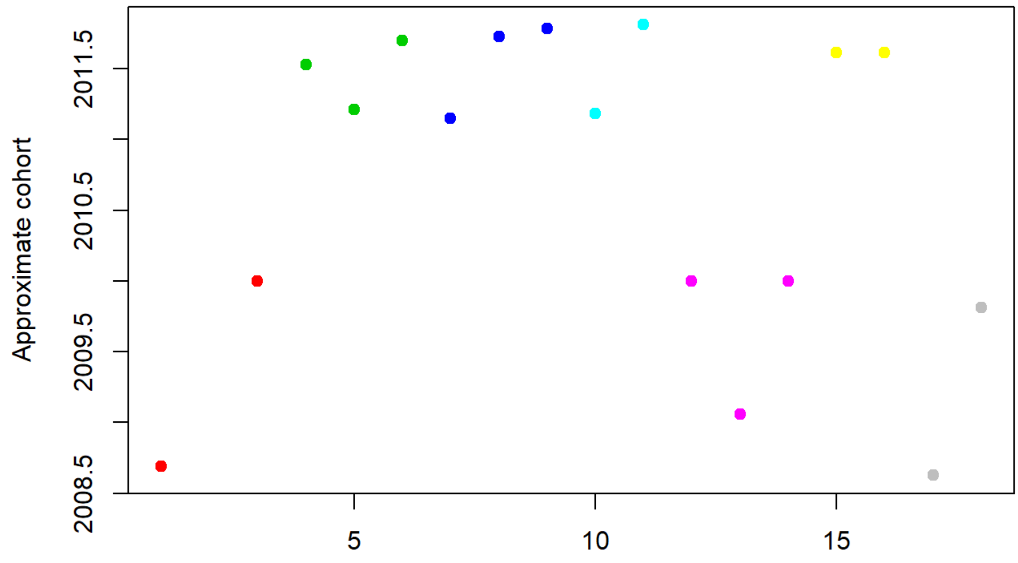

The points are coloured according to FS group

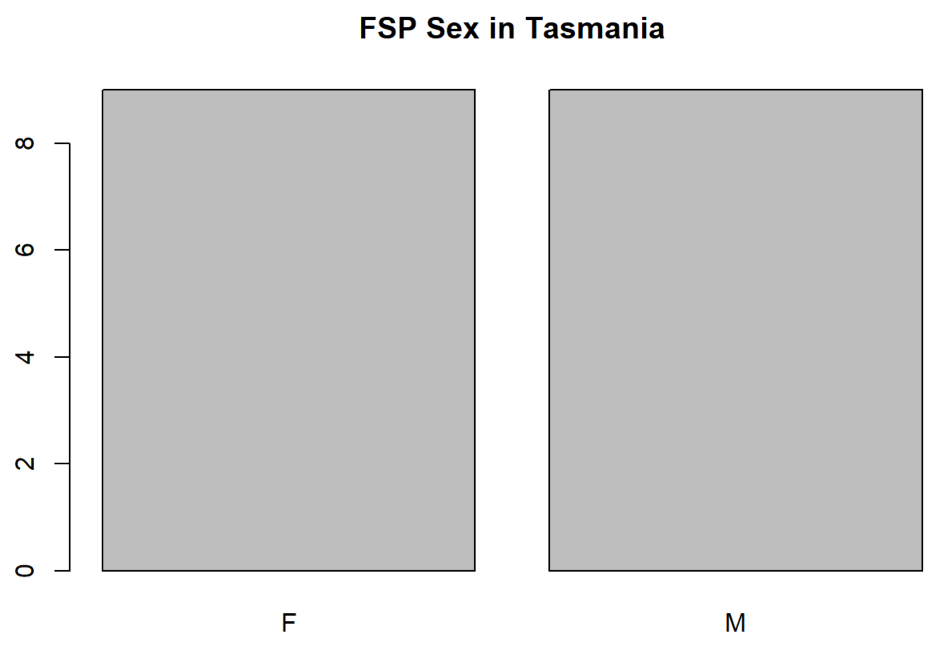

5.4 Filtering

5.4.1 Filter for duplicate loci/CloneID

Removes SNPs on the same cloneID.

```
gl <- dartR::gl.filter.secondaries(gl, v = 5)
```

```
## Starting gl.filter.secondaries: Deleting all but one SNP per sequence tag
##   Total number of SNP loci: 31550
##   Selecting one SNP per sequence tag at random
##     Number of secondaries: 7046
##     Number of loci after secondaries removed: 24504
## gl.filter.secondaries completed
```

5.4.2 Monomorphic loci

```
gl <- dartR::gl.filter.monomorphs(gl, v = 5)
```

```
## Starting gl.filter.monomorphs: Deleting monomorphic loci
##   Polymorphic loci: 20953
##   Monomorphic loci: 3551
##   Loci with no scores (all NA): 0
##   Deleting monomorphic loci and loci with all NA scores
## Completed gl.filter.monomorphs
```

5.4.3 Filter on Callrate

Checks how much missing data you have.

> If you have individuals with many missing data, it might be good to first filter on individuals (e.g. 0.80), then loci (0.80), then ind (0.85), then loci (0.85), etc....

```
dartR::gl.report.callrate(gl, method = "loc")
```

```
## Starting gl.report.callrate
## Starting utils.recalc.callrate: Recalculating CallRate
## Completed utils.recalc.callrate
```

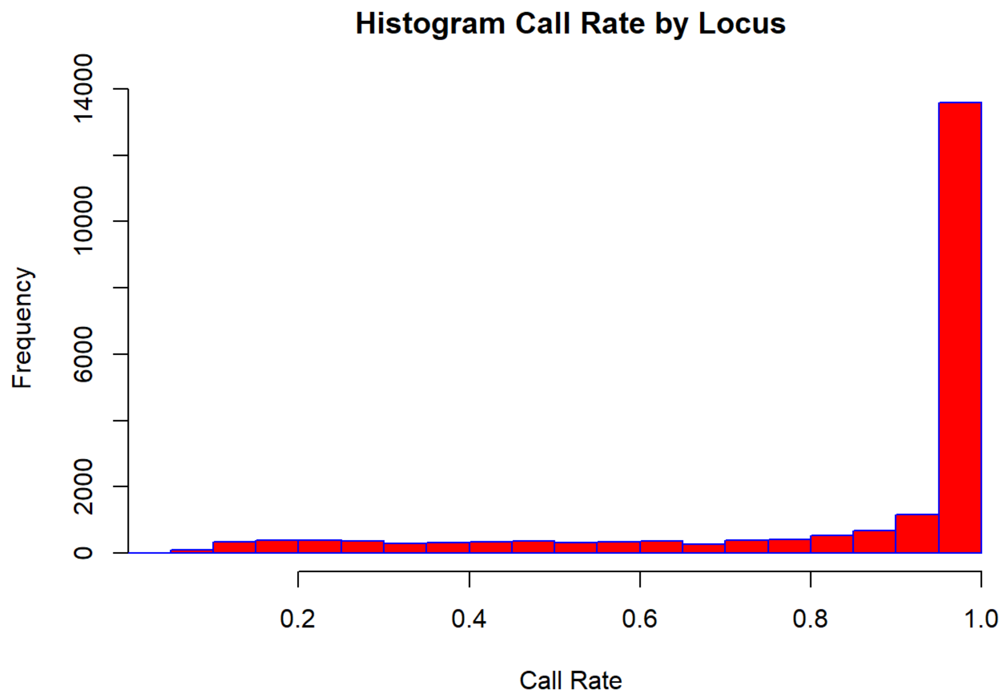


```
##   Loci with no missing values = 11000 [52.5%]
##     < 5% missing values = 13579 [64.8%]
##     < 10% missing values = 14732 [70.3%]
##     < 15% missing values = 15410 [73.5%]
##     < 20% missing values = 15938 [76.1%]
##     < 25% missing values = 16361 [78.1%]
##     < 30% missing values = 16755 [80%]
##     < 35% missing values = 17019 [81.2%]
##     < 40% missing values = 17391 [83%]
##     < 45% missing values = 17741 [84.7%]
##     < 50% missing values = 18053 [86.2%]
##     < 55% missing values = 18414 [87.9%]
##     < 60% missing values = 18751 [89.5%]
##     < 65% missing values = 19077 [91%]
##     < 70% missing values = 19368 [92.4%]
##     < 75% missing values = 19739 [94.2%]
##     < 80% missing values = 20121 [96%]
##     < 85% missing values = 20510 [97.9%]
##     < 90% missing values = 20842 [99.5%]
##     < 95% missing values = 20952 [100%]
## gl.report.callrate Completed
```

```
## NULL
```

```
dartR::gl.report.callrate(gl, method = "ind")
```

```
## Starting gl.report.callrate
## Starting utils.recalc.callrate: Recalculating CallRate
## Completed utils.recalc.callrate
```

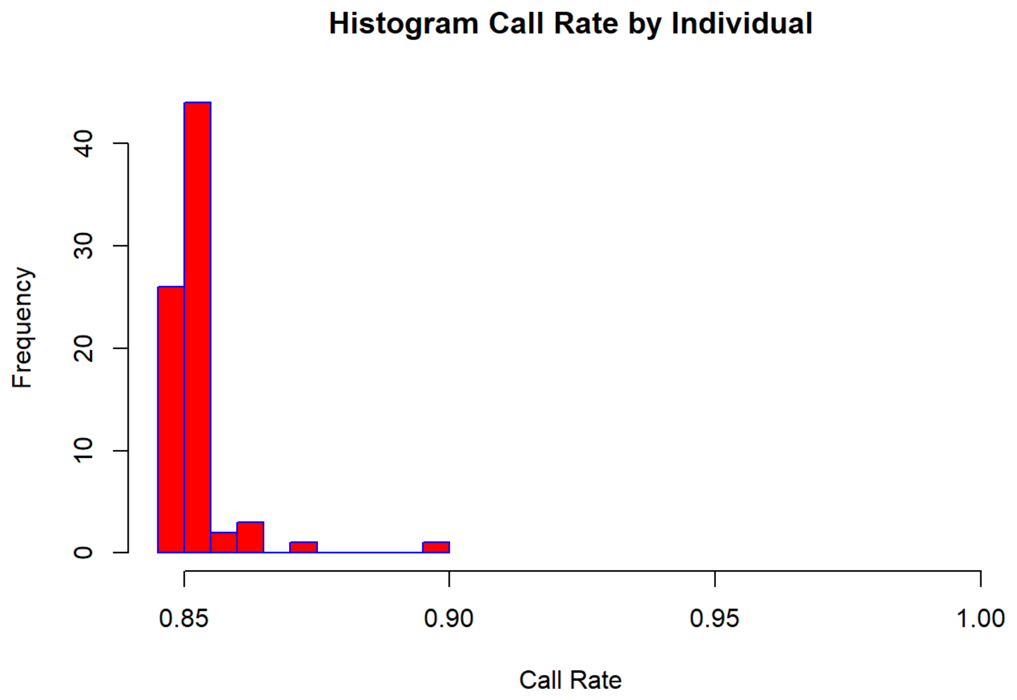


```
## Individuals no missing values = 0 [0%] across loci; all individuals would be filtered
##      with less than or equal to 5% = 0 [0%]; 77 individuals would be filtered
##      with less than or equal to 10% = 0 [0%]; 77 individuals would be filtered
##      with less than or equal to 15% = 51 [66.2%]; 26 individuals would be filtered
##      with less than or equal to 20% = 77 [100%]; 0 individuals would be filtered
## gl.report.callrate Completed
```

```
## NULL
```

```
gl <- gl.filter.missing.data.FDD(gl, loc.lb = 0.5, loc.hb = 0.95,
    ind.lb = 0.5, ind.hb = 0.95, iterations = 100)
```

```
## Summary of filtered dataset
##   SNPs with CallRate > 0.95 : 13579
##   Individuals with CallRate > 0.95 : 77
##   No. of loci removed: 7374
##   No. of individuals removed: 0
```

5.4.4 Individual Heterozygosity

Delete individuals with a heterozygosity above the threshold. High heterozygozity could be due to cross-contamination from other individuals.

```
gl <- gl.filter.het.FDD(gl, LowerT = 0.1, UpperT = 0.2)
```

```
## Reporting for a genlight object
## Initial no. of individuals = 77
## Filtering a genlight object
##   no. of individuals deleted = 1
## Individuals retained = 76
##
## List of individuals deleted because of high heterozygosity
##  SsTAS_Bx02_E10
##
##    from populations
##  TAS
##
## Summary of filtered dataset
## 0.1 <  Individuals with heterozygosity <= 0.2
##   No. of loci: 13579
##   No. of individuals: 76
##   No. of populations:  2
```

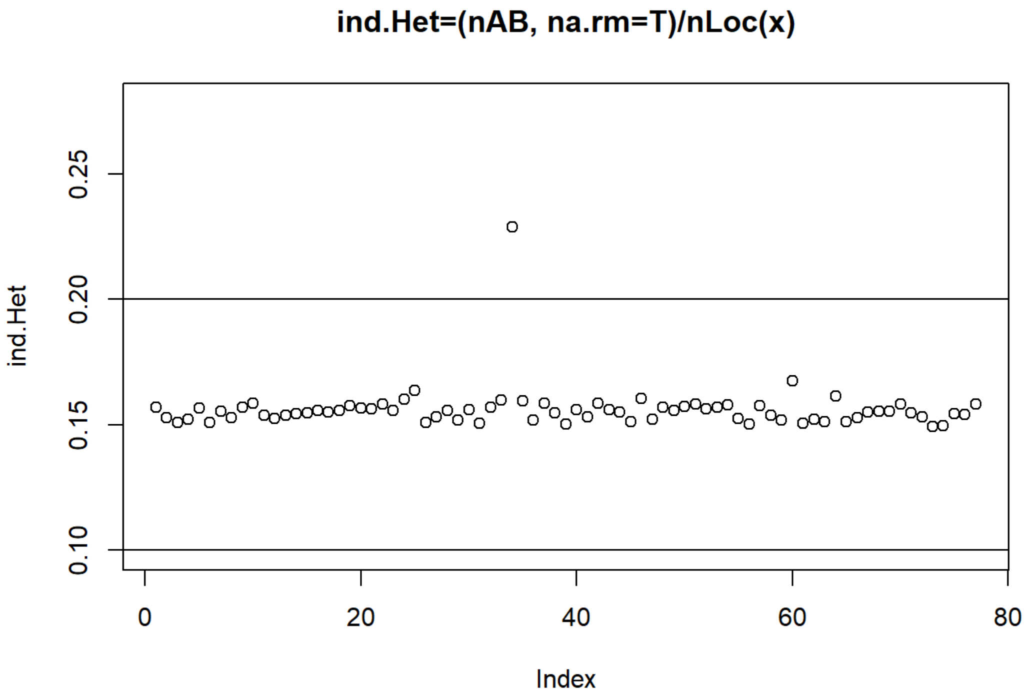

5.4.5 Monomorphic loci

Do an additional monomorph filter after you removed several individuals.

```
gl <- dartR::gl.filter.monomorphs(gl, v = 5)
```

```
## Starting gl.filter.monomorphs: Deleting monomorphic loci
##   Polymorphic loci: 13555
##   Monomorphic loci: 24
##   Loci with no scores (all NA): 0
##   Deleting monomorphic loci and loci with all NA scores
## Completed gl.filter.monomorphs
```

5.4.6 Filter on average reproducability

DArT includes several duplicates of your samples to see how reproducable they results are.

```
dartR::gl.report.repavg(gl)
```

```
## Reporting for a genlight object
## Note: RepAvg is a DArT statistic reporting reproducibility averaged across alleles for each locus
.
##
## No. of loci = 13555
##
##   Loci with perfect reproducibility = 11005 [81.2%]
##   > 0.995 = 11005 [81.2%]
##   > 0.99 = 11005 [81.2%]
##   > 0.985 = 13199 [97.4%]
##   > 0.98 = 13237 [97.7%]
##   > 0.975 = 13237 [97.7%]
##   > 0.97 = 13555 [100%]
```

```
## [1] "Completed"
```

```
gl <- dartR::gl.filter.repavg(gl, t = 0.98, v = 5)
```

```
## Starting gl.filter.repavg: Filtering on repeatability
## Note: RepAvg is a DArT statistic reporting repeatability averaged across alleles for each locus.
##
## Initial no. of loci = 13555
##   Removing loci with RepAvg < 0.98
## No. of loci deleted = 318
## Summary of filtered dataset
##   Repeatability >= 0.98
##   No. of loci: 13237
##   No. of individuals: 76
##   No. of populations:  2
## gl.filter.repavg completed
```

5.4.7 Counts

Check the histogram to see how many SNP have low or high counts.

```
hist(gl@other$loc.metrics[, "SumCount"], breaks = 20, plot = T,
    main = "Histogram of read depth of reference and SNP allele")
```

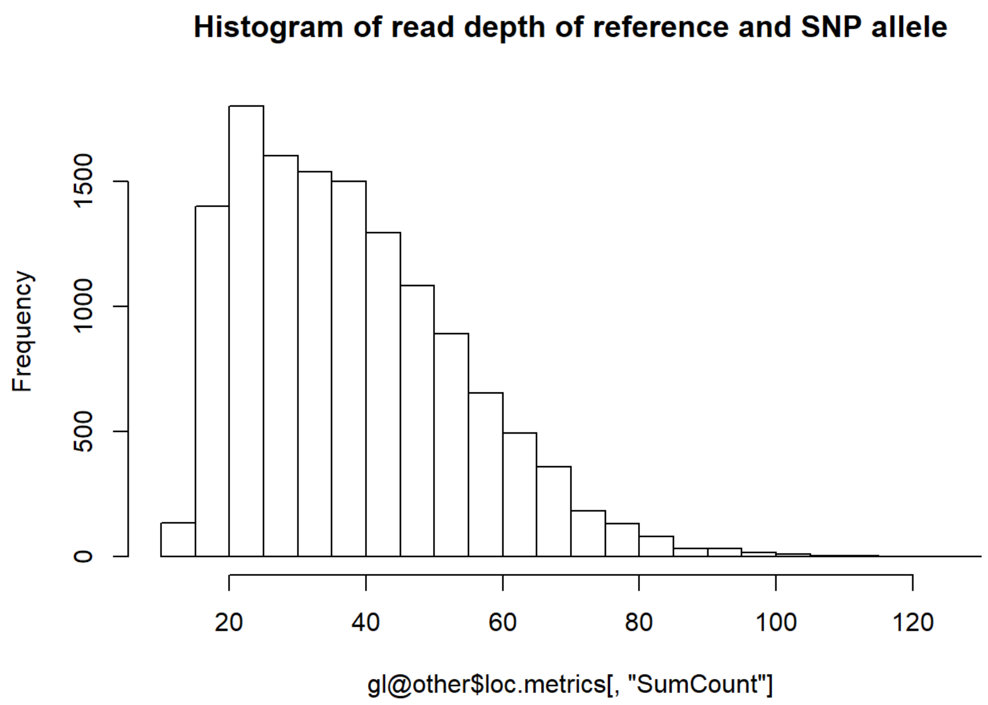

5.4.7.1 Low counts

Low counts might not be reliably genotyped.

```
gl <- gl.filter.lowcount.FDD(gl, threshold = 15)
```

```
## Total number of SNP loci: 13237
## No. of loci deleted = 134
##   Read depth >= 15
##   No. of loci: 13103
##   No. of individuals: 76
##   No. of populations:  2
```

5.4.7.2 High Counts

High counts might be due to paralogous fragments.

```
gl <- gl.filter.highcount.FDD(gl, threshold = 90)
```

```
## Total number of SNP loci: 13103
## No. of loci deleted = 72
##   Read depth <= 90
##   No. of loci: 13031
##   No. of individuals: 76
##   No. of populations:  2
```

5.4.8 Minor allele frequency

Loci with a low MAF might not be informative enough and just increase computation time.

```
gl <- gl.filter.maf.FDD(gl, threshold = 0.05)
```

```
## Filtering a genlight object
## Total number of SNP loci: 13031
##    Number of loci with MAF < 0.05 : 6428
##    Number of loci after filtering: 6603
##    Number of individuals: 76
##    Number of populations:  2
```

5.4.9 Heterzygozity per SNP

SNPs that are too heterozygous could be due to paralogous reads.

```
gl <- gl.filter.loc.het.FDD(gl, threshold = 0.6)
```

```
## Total number of SNP loci: 6603
## No. of loci deleted = 9
##   SNPs with heterozygosity <= 0.6
##   No. of loci: 6594
##   No. of individuals: 76
##   No. of populations:  2
```

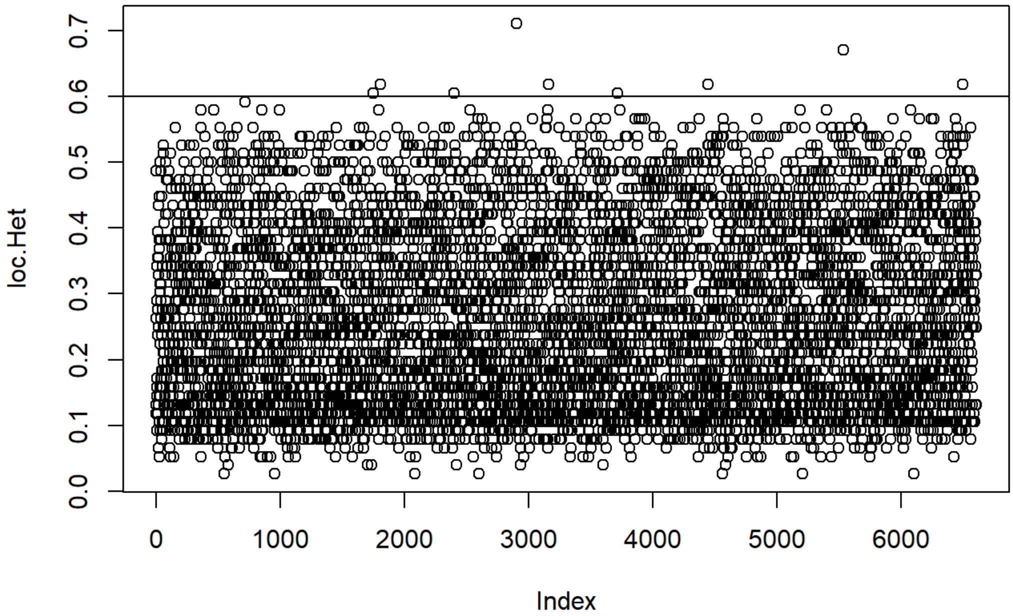

5.5 Convert to other data types

```
gi <- gl2gi_mvb(gl)
gl2Adm(gl, filename = "SS_noFS_ADMIXTURE-PCadapt")
```

5.6 Calculate outliers

5.6.1 OutFLANK

```
SNPmat <- as.matrix(gl)
colnames(SNPmat) <- NULL
row.names(SNPmat) <- NULL
SNPmat[is.na(SNPmat)] <- 9
FstDataFrame <- OutFLANK::MakeDiploidFSTMat(SNPmat, gl$loc.names,
    as.character(gl$pop))
```

```
## Calculating FSTs, may take a few minutes...
```

```
plot(FstDataFrame$FST, FstDataFrame$FSTNoCorr, xlim = c(-0.01,
    0.3), ylim = c(-0.01, 0.3), pch = 20) + abline(0, 1)
```

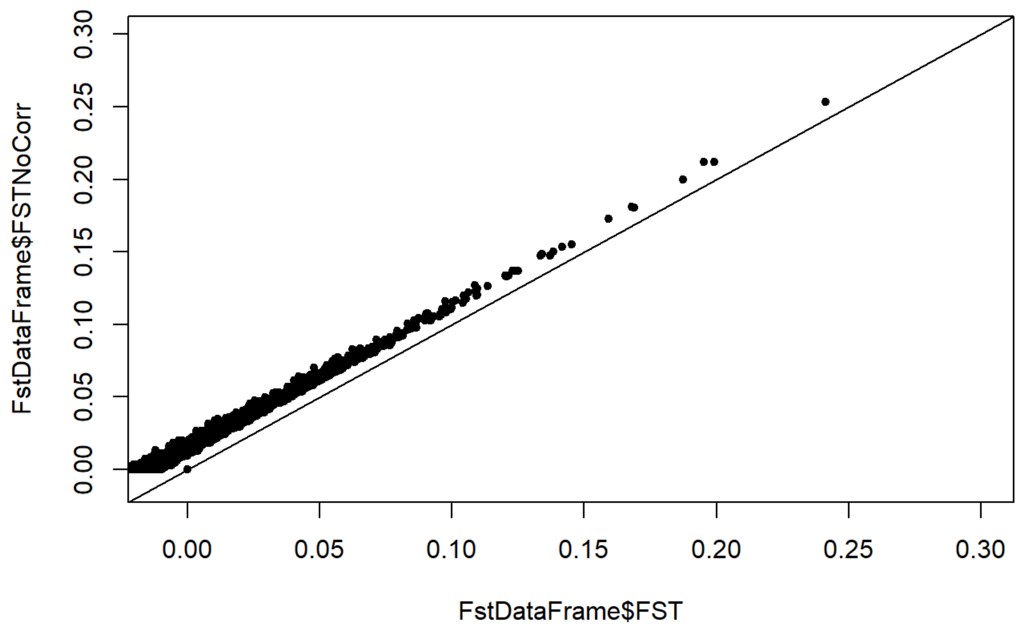


```
## integer(0)
```

```
hist(FstDataFrame$FSTNoCorr)
```

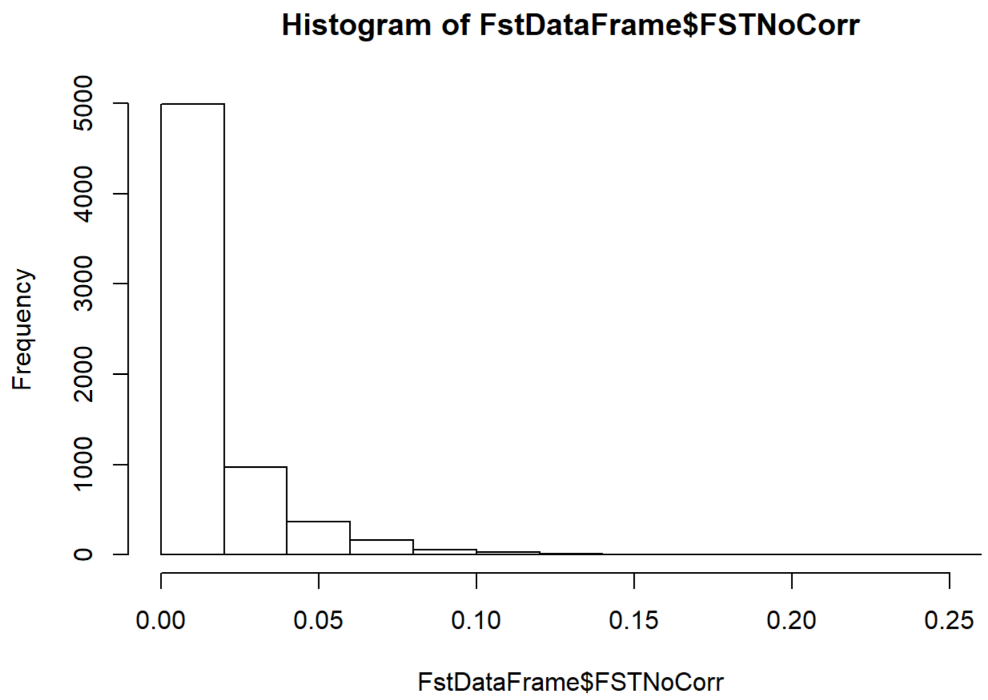


```
Outliers <- OutFLANK(FstDataFrame, NumberOfSamples = length(levels(gl@pop)),
    qthreshold = 0.01, LeftTrimFraction = 0.01, RightTrimFraction = 0.01,
    Hmin = 0.001)

OutFLANKResultsPlotter(Outliers, withOutliers = TRUE, NoCorr = TRUE,
    Hmin = 0.1, binwidth = 0.005, Zoom = FALSE, RightZoomFraction = 0.05,
    titletext = NULL)
```

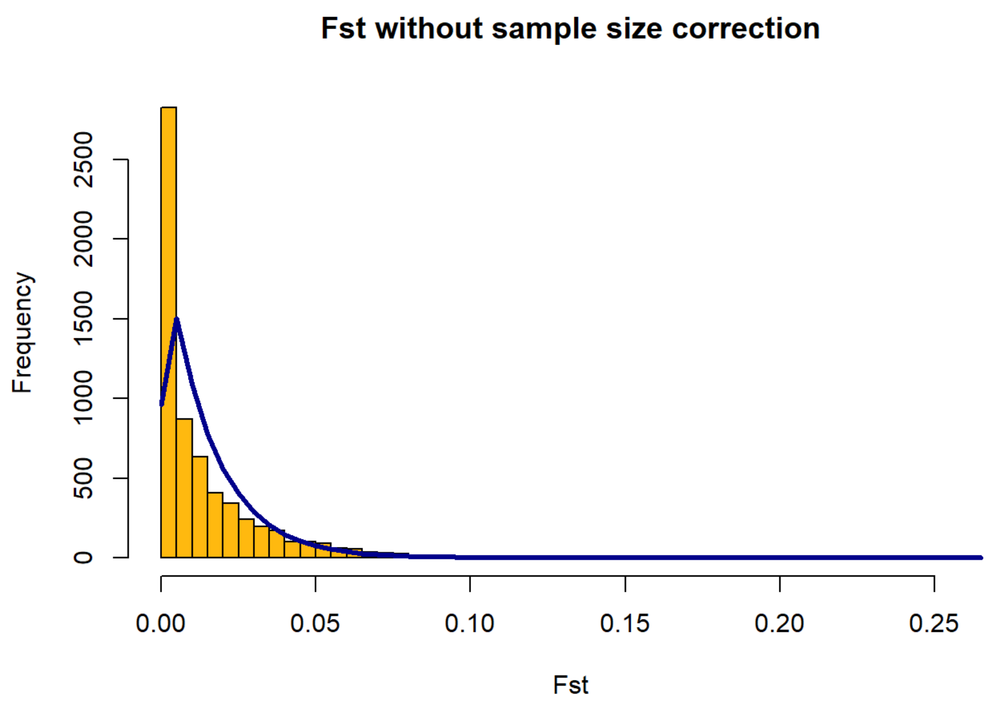


```
Outliers$numberLowFstOutliers
```

```
## [1] 0
```

```
Outliers$numberHighFstOutliers
```

```
## [1] 7
```

```
OutflankNames <- Outliers$results$LocusName[Outliers$results$OutlierFlag ==
    TRUE]
print(OutflankNames)
```

```
## [1] 12871469-48-G/A 12863758-49-C/T 12863555-66-C/A
## [4] 12868007-12-A/G 12863843-5-A/G  12858443-53-T/G
## [7] 12875588-49-A/G
## 6594 Levels: 100026312-20-A/G ... 12875816-9-T/C
```

5.6.2 PCadapt

```
data <- read.pcadapt("SS_noFS_ADMIXTURE-PCadapt.ped", type = "ped")  #samples in columns and Loci in
Rows
```

```
## Summary:
##
##  - input file:               SS_noFS_ADMIXTURE-PCadapt.ped
##  - output file:
C:\Users\fdevloo\AppData\Local\Temp\RtmpAF4bvo\file4144709bebd.pcadapt
##
##  - number of individuals detected:   76
##  - number of loci detected:      6594
##
## 6594 lines detected.
## 76 columns detected.
```

```
popdata <- read.table("SS_noFS_ADMIXTURE-PCadapt.ped")
poplist <- popdata[, 1]

K <- 25
x <- pcadapt(data, K = K, min.maf = 0.05)
x$singular.values
```

```
##  [1] 0.1344268 0.1328326 0.1322850 0.1314029 0.1305775
##  [6] 0.1303180 0.1299112 0.1295017 0.1292175 0.1290109
## [11] 0.1287586 0.1283473 0.1282017 0.1277556 0.1275166
## [16] 0.1271523 0.1266215 0.1263450 0.1256681 0.1256194
## [21] 0.1253760 0.1250427 0.1249225 0.1245370 0.1239461
```

```
plot(x, option = "screeplot")  #K = 8
```

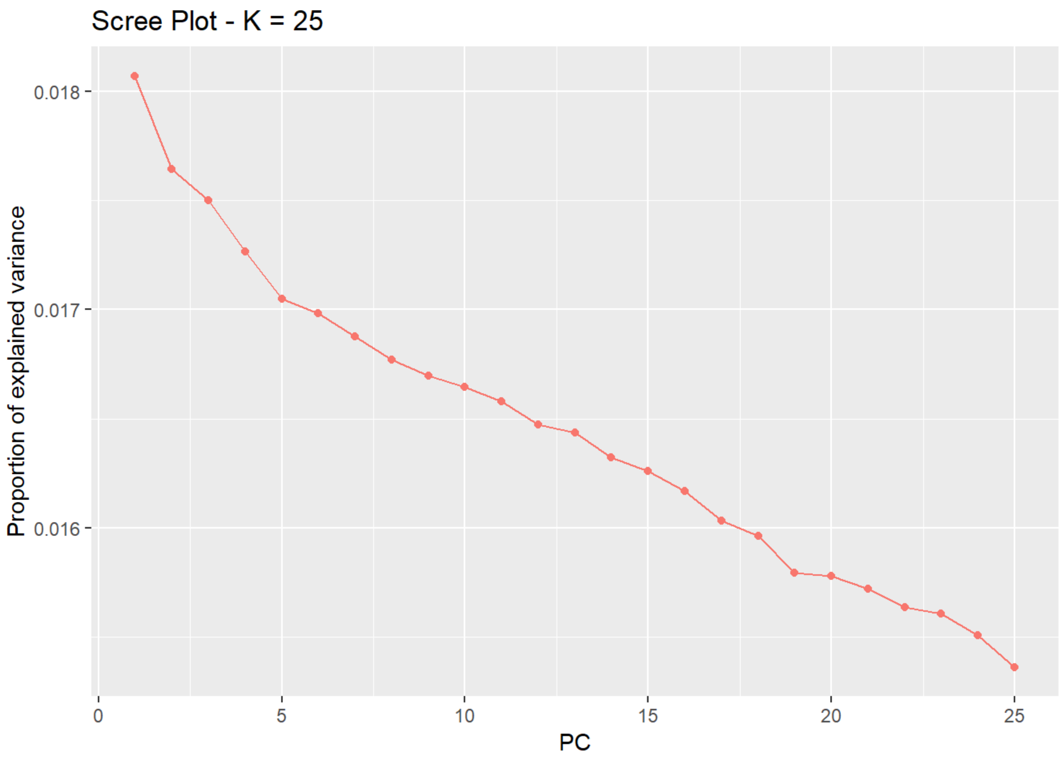


```
plot(x, option = "scores", i = 1, j = 2, pop = poplist)
```

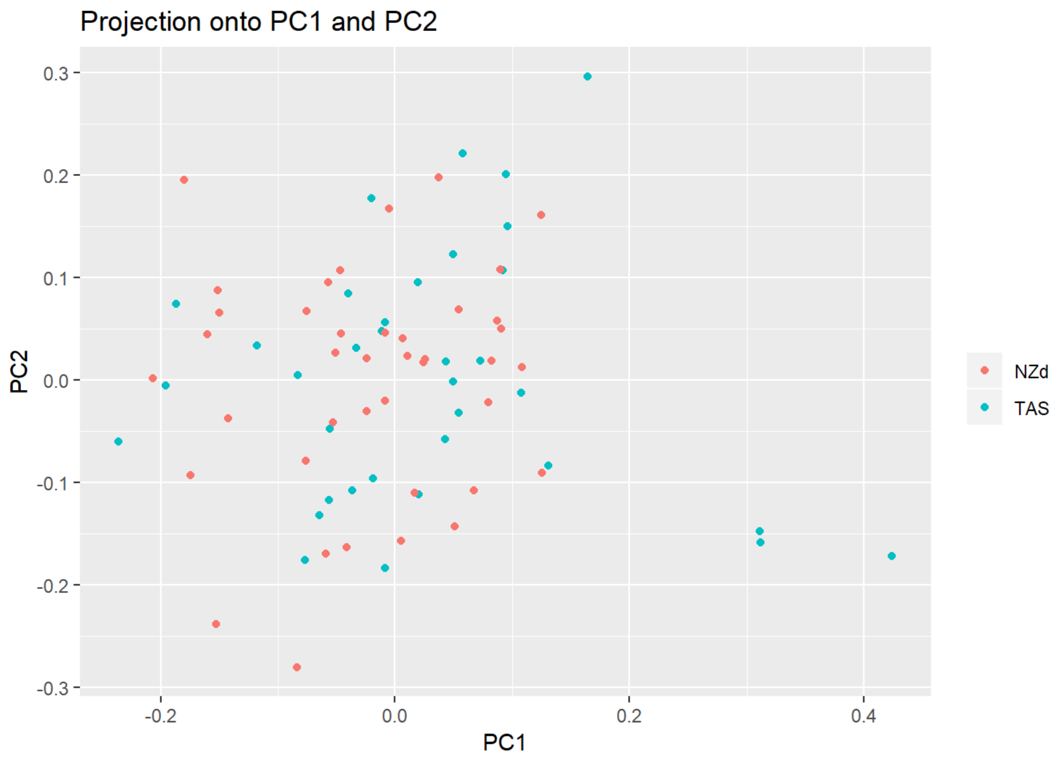


```
plot(x, option = "scores", i = 1, j = 3, pop = poplist)
```

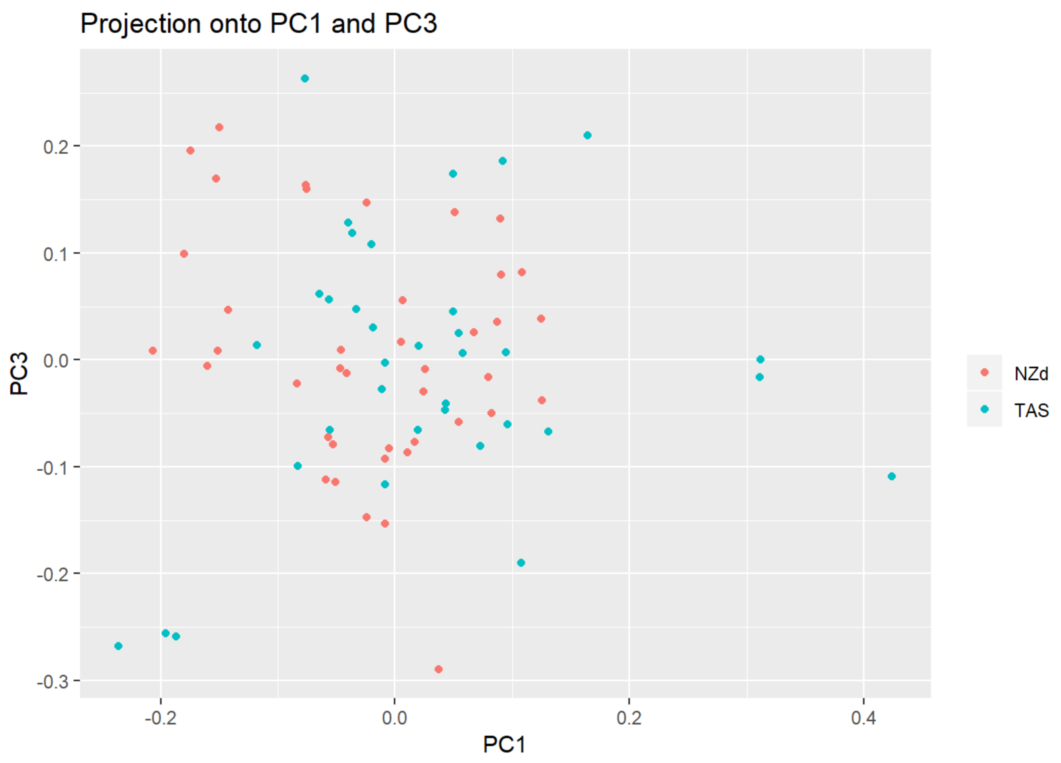


```
plot(x, option = "scores", i = 2, j = 3, pop = poplist)
```

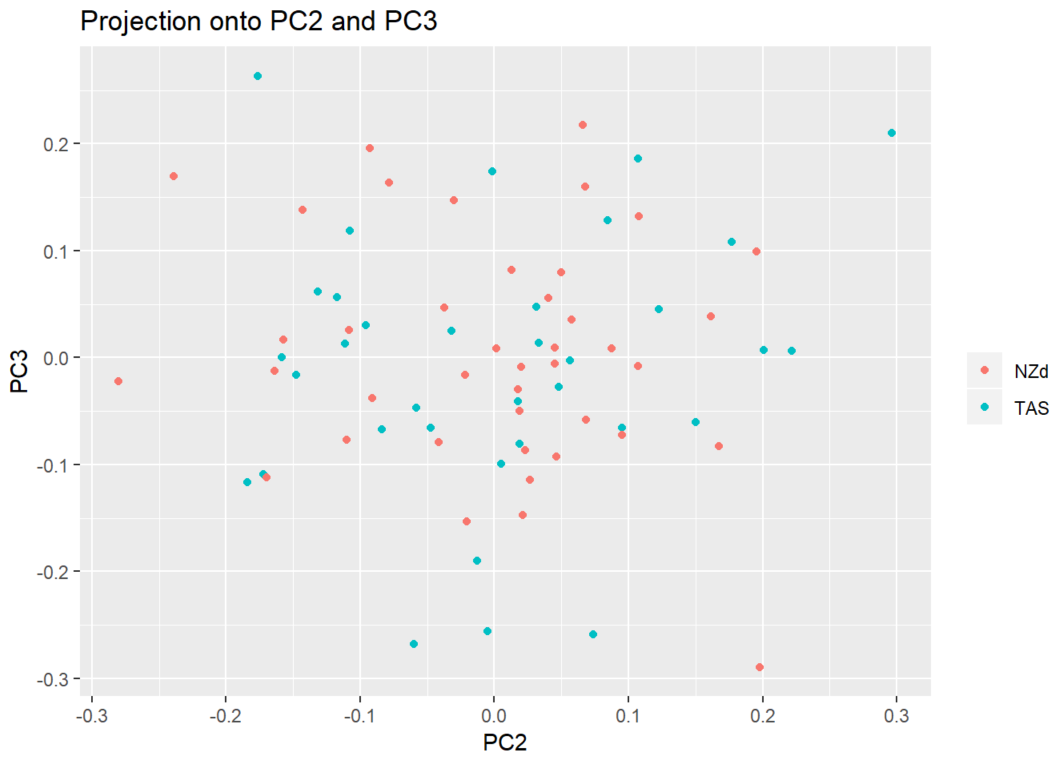


```
plot(x, option = "scores", i = 3, j = 4, pop = poplist)
```

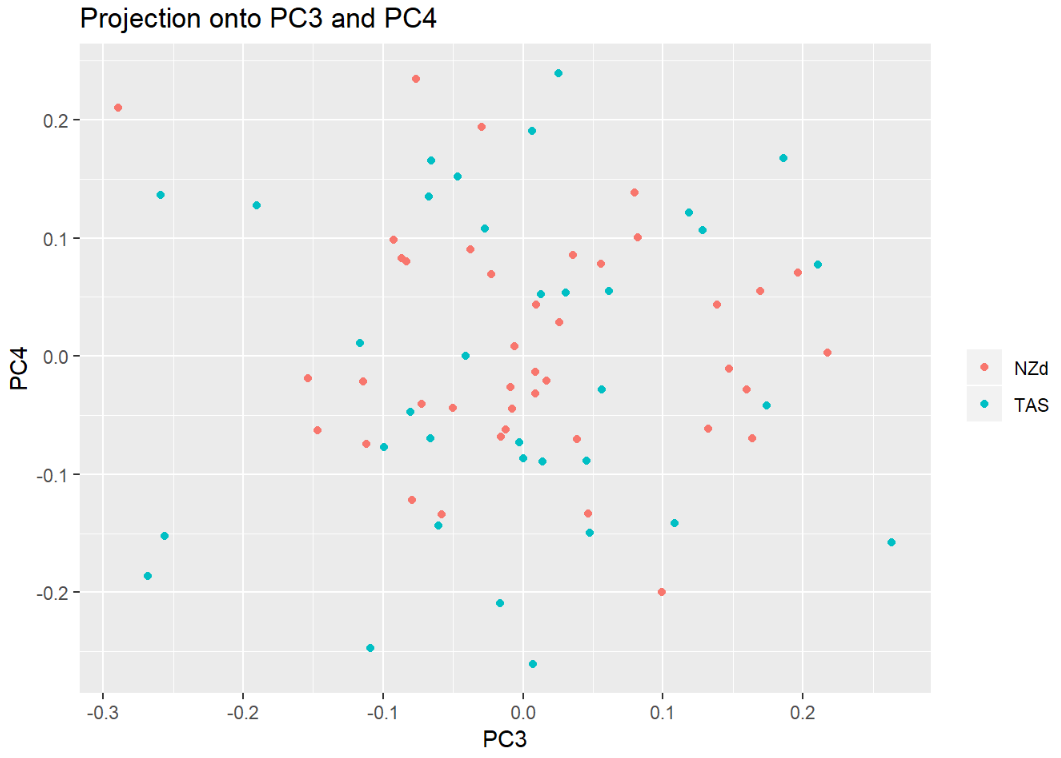


```
K <- 8
x <- pcadapt(data, K = K, min.maf = 0.05)
x$singular.values
```

```
## [1] 0.1344268 0.1328326 0.1322850 0.1314034 0.1305775
## [6] 0.1303180 0.1299111 0.1295014
```

```
plot(x, option = "manhattan")
```

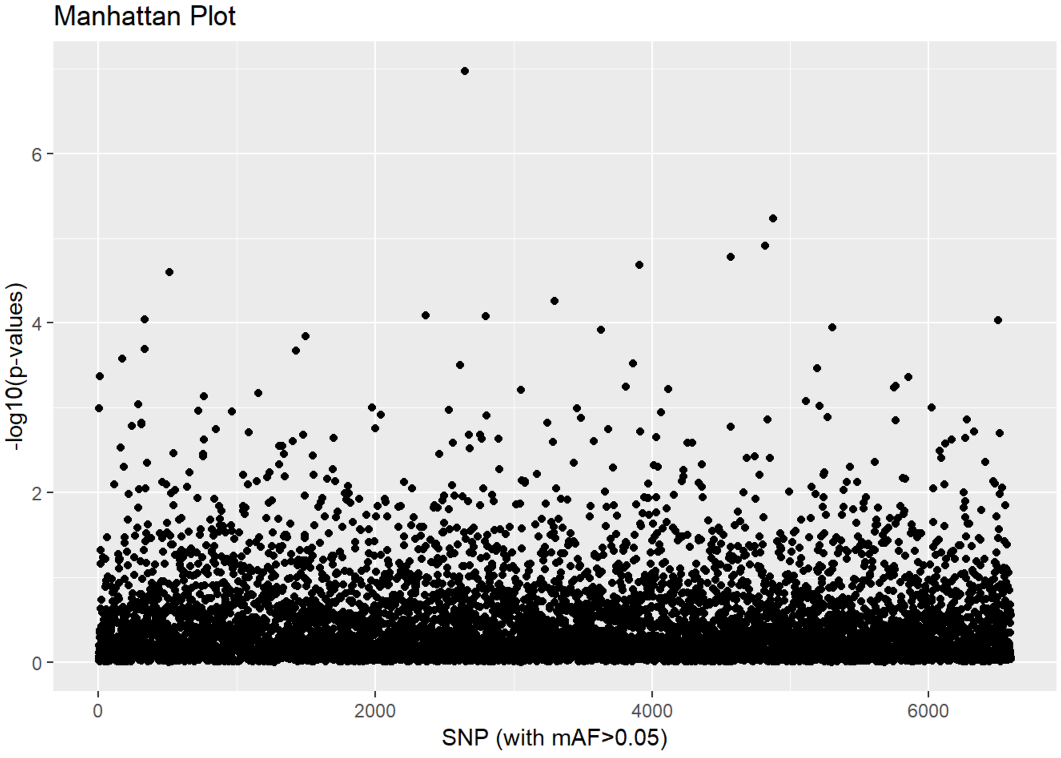


```r
plot(x, option = "qqplot", threshold = 0.05)
```

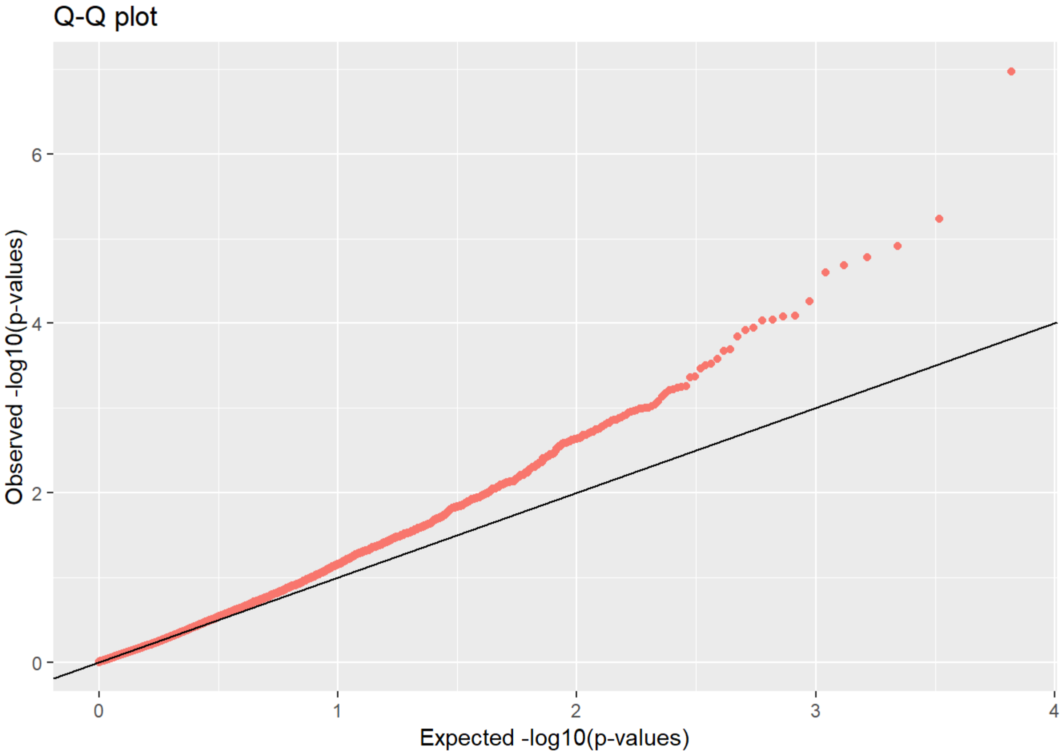


```r
hist(x$pvalues, xlab = "p-values", main = NULL, breaks = 50)
```

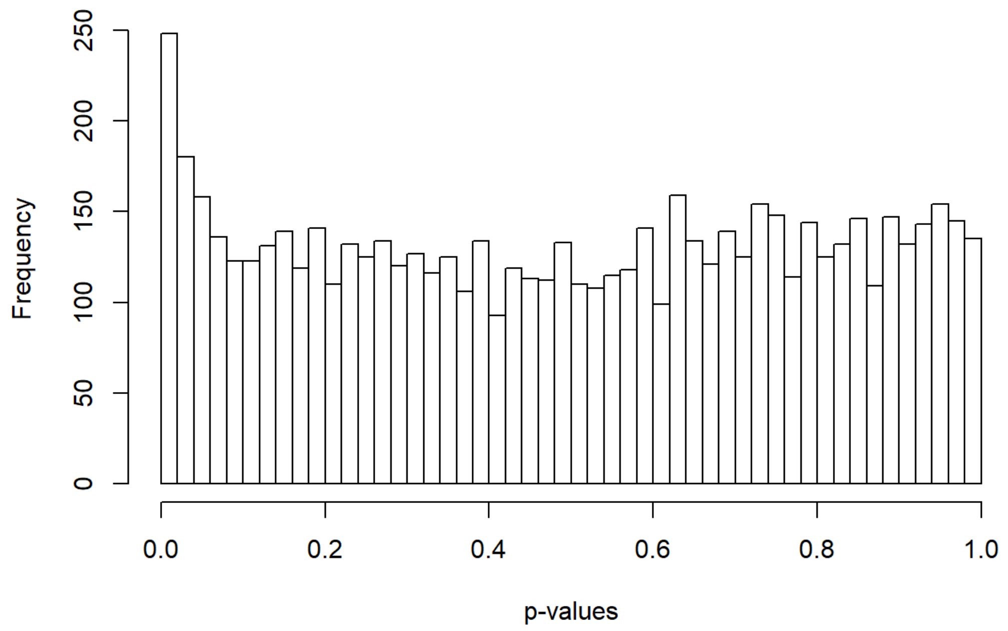


```
plot(x, option = "stat.distribution")
```

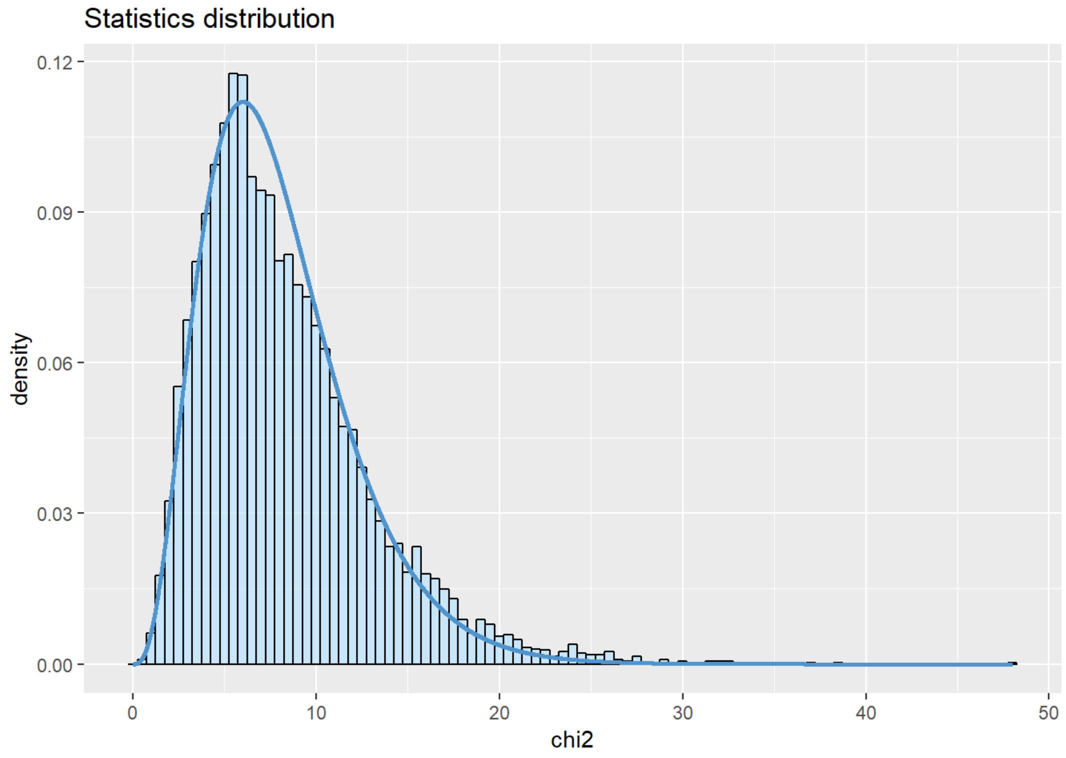


```
qval <- qvalue(x$pvalues)$qvalues
alpha <- 0.01
pcadapt <- which(qval < alpha)
length(pcadapt)
```

```
## [1] 1
```

```
PCadaptkNames <- gl$loc.names[pcadapt]
print(PCadaptkNames)
```

```
## [1] "12870391-59-A/C"
```

5.6.3 Remove outliers

First we check if there is any overlap between the two methods for detecting outliers.
Since OutFLANK is more stringent, we chose to only remove outliers based on OutFLANK

```
PCadaptkNames %in% OutflankNames
```

```
## [1] FALSE
```

```
OutflankNames %in% PCadaptkNames
```

```
## [1] FALSE FALSE FALSE FALSE FALSE FALSE FALSE
```

```
rm <- gl$loc.names %in% OutflankNames
gl <- gl[, !rm]
gl$other$loc.metrics <- gl$other$loc.metrics[!rm, ]
```

5.7 Export data to other software formats

```
gi <- gl2gi_mvb(gl)
save(gl, gi, file = "gl-gi_noFS.rdata")
# load('gl-gi_noFS.rdata')
gl2gpop(gl, filename = "SS_noFS_genepop.txt")
gl2Adm(gl, filename = "SS_noFS_ADMIXTURE-PCadapt")
dartR::gl2structure(gl, outfile = "SS_noFS_STRUCTURE.txt", outpath = getwd())
```

```
## Structure file saved as: SS_noFS_STRUCTURE.txt
## in folder: C:/Users/fdevloo/OneDrive - University of Tasmania/PhD/School shark/R-analysis
```

5.8 Genetic diversity

```
set.seed(124)
bastat <- diveRsity::basicStats(infile = "SS_noFS_genepop.txt",
    outfile = "SS_noFS_BasicStat_output.txt", fis_ci = T, ar_ci = T,
    fis_boots = boots, ar_boots = boots, mc_reps = boots, rarefaction = F,
    ar_alpha = 0.05, fis_alpha = 0.05)

gendiv <- data.frame(Tasmania = bastat$main_tab$SsTAS_Bx02_A01$overall,
    `New Zealand` = bastat$main_tab$SsNZd_Bx03_A01$overall, Combined =
rowMeans(cbind(bastat$main_tab$SsTAS_Bx02_A01$overall,
        bastat$main_tab$SsNZd_Bx03_A01$overall)))
row.names(gendiv) <- rownames(bastat$main_tab$SsTAS_Bx02_A01)
knitr::kable(gendiv, caption = "Genetic diversity without full siblings")
```

Genetic diversity without full siblings

	Tasmania	New.Zealand	Combined
ar	1.990	1.993	1.9915
size	34.844	40.820	37.8320
obs_het	0.265	0.264	0.2645
exp_het	0.284	0.285	0.2845
uexp_het	0.288	0.288	0.2880

	Tasmania	New.Zealand	Combined
fis	0.065	0.067	0.0660
hwe_glb	1.000	1.000	1.0000
hwe_hom	1.000	1.000	1.0000
hwe_het	1.000	0.012	0.5060
fis_lo	0.040	0.046	0.0430
fis_hi	0.057	0.060	0.0585
ar_lo	1.981	1.986	1.9835
ar_hi	1.995	1.997	1.9960

5.9 Fixation and differentiation indices

5.9.1 Global

```
set.seed(124)
pwfst <- StAMPP::stamppFst(gl, nboots = boots, percent = 95,
    nclusters = 3)
Fst <- c(pwfst$Bootstraps$Fst, pwfst$Bootstraps$`p-value`, pwfst$Bootstraps$`Lower bound CI limit`,
    pwfst$Bootstraps$`Upper bound CI limit`)
names(Fst) <- c("Fst", "P-value", "Lower CI", "Upper CI")
print(Fst)
```

```
##           Fst       P-value      Lower CI      Upper CI
##  0.0003374980  0.1163000000 -0.0002141734  0.0008846968
```

```
bs <- mmod::chao_bootstrap(gi, nreps = boots2)
bs.D <- mmod::summarise_bootstrap(bs, D_Jost)
```

```
## Warning in mmod::summarise_bootstrap(bs, D_Jost): Bootstrap
## distribution of D_Jost includes negative values, harmonic
## mean is undefined
```

```
print(bs.D$summary.global.het)
```

```
##         observed      lower.normal      upper.normal
##     0.0005934471      0.0001978329      0.0009890614
##          std.dev              mean  lower.percentile
##     0.0002018440      0.0059732370      0.0055547279
## upper.precentile
##     0.0063574134
```

```
nc.diff_stats <- mmod::diff_stats(gi, phi_st = TRUE)
print(nc.diff_stats$global)
```

```
##           Hs           Ht      Gst_est     Gprime_st
## 0.2945574980 0.2947668194 0.0007101254 0.0020118479
##        D_het       D_mean       Phi_st
## 0.0005934471           NA 0.0010201417
```

```
with(nc.diff_stats, pairs(per.locus[, 3:6], upper.panel = panel.smooth))
```

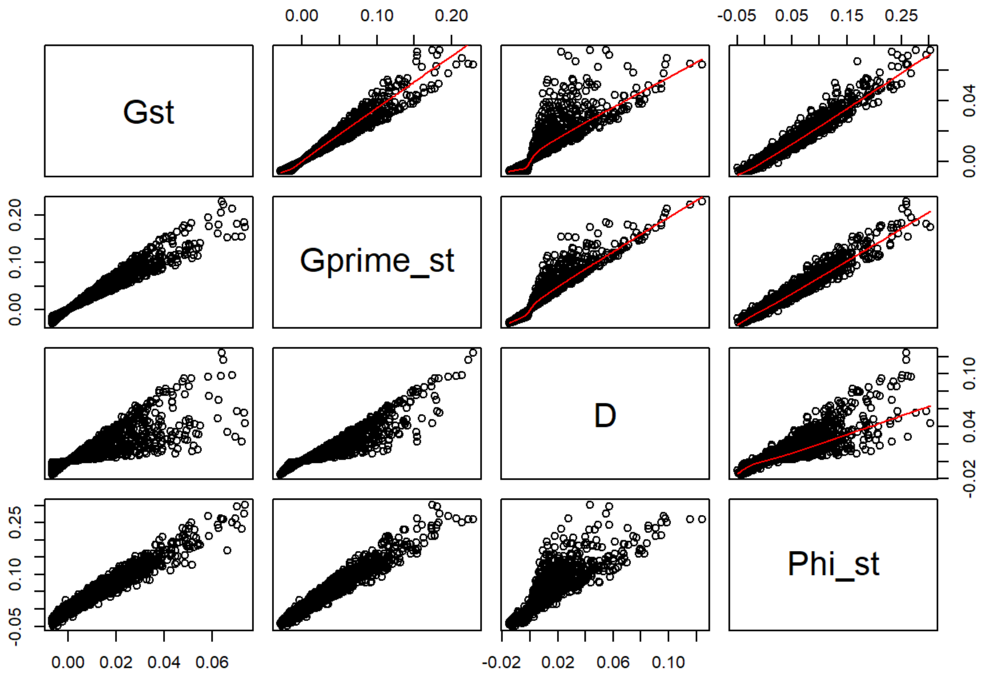

5.9.2 Pairwise

```
set.seed(124)
knitr::kable(rbind(pwfst$Fsts, pwfst$Pvalues), digits = 5, caption = " Weir and Cockerham's Fst (abo
ve) and p-values (below)")
```

Weir and Cockerham's Fst (above) and p-values (below)

	TAS	NZd
TAS	NA	NA
NZd	0.00034	NA
TAS	NA	NA
NZd	0.11630	NA

```
PD <- as.matrix(mmod::pairwise_D(gi, linearized = FALSE, hsht_mean = "arithmetic"))
knitr::kable(PD, digits = 5, caption = "Jost D - arithmetic mean")
```

Jost D - arithmetic mean

	TAS	NZd
TAS	0.00000	0.00059
NZd	0.00059	0.00000

5.10 DAPC

5.10.1 DAPC with location prior

5.10.1.1 Cross-validation to identify the optimal number of PC's

Uses a training - holdout set of individuals to check how reliable individuals can be assigned.

```
x <- gl
set.seed(124)
xval <- adegenet::xvalDapc(tab(x, NA.method = "mean"), pop(x),
    n.pca.max = nInd(x), training.set = 0.9, result = "groupMean",
    center = TRUE, scale = FALSE, n.rep = boots, xval.plot = TRUE)
```

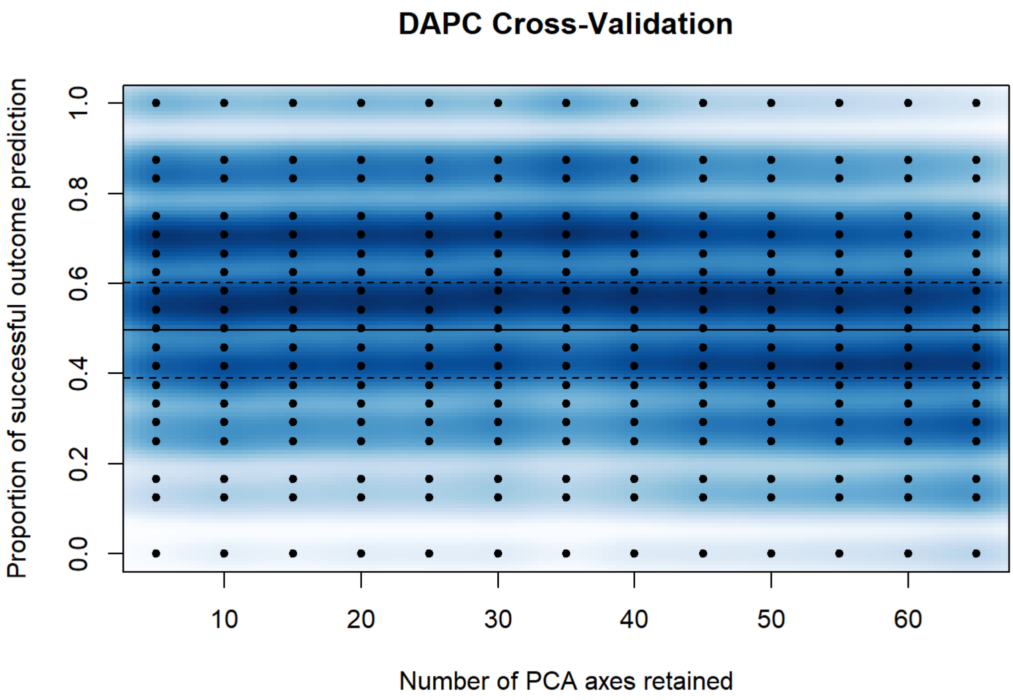


```
print(xval[2:6])
PC <- as.numeric(xval$`Number of PCs Achieving Lowest MSE`)

# refine the analysis
xval <- adegenet::xvalDapc(tab(x, NA.method = "mean"), pop(x),
    n.pca = (PC - 15):(PC + 15), n.rep = boots, parallel = "multicore",
    ncpus = 3L, xval.plot = TRUE)
```

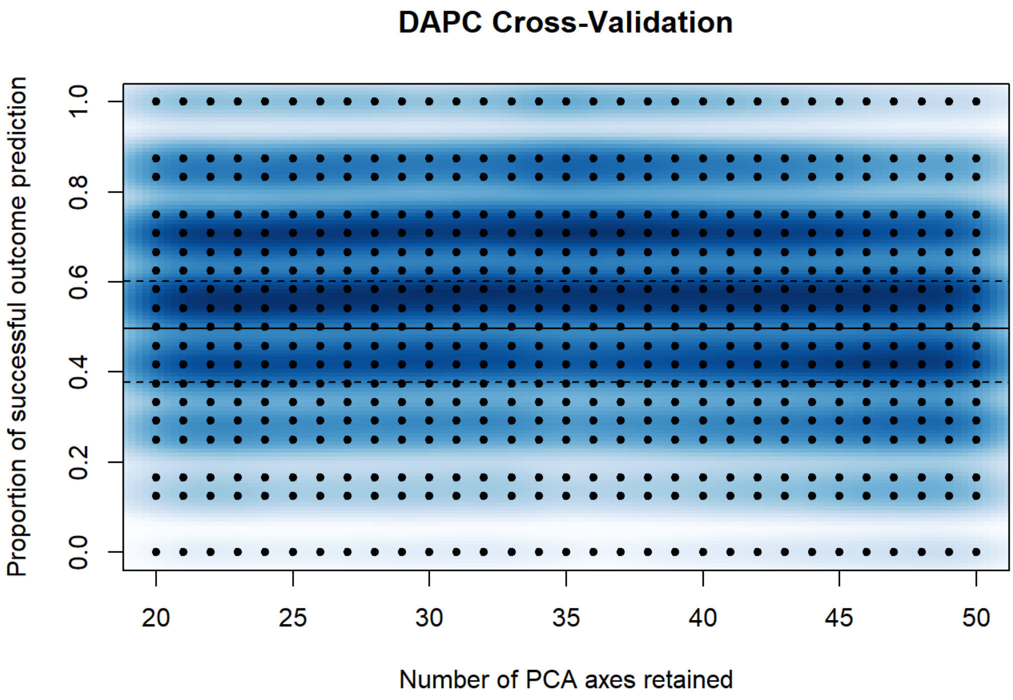

```
print(xval[2:6])
PC <- as.numeric(xval$`Number of PCs Achieving Lowest MSE`)
```

```
## $`Median and Confidence Interval for Random Chance`
##      2.5%       50%     97.5%
## 0.3909408 0.4968641 0.6027875
##
## $`Mean Successful Assignment by Number of PCs of PCA`
##         5        10        15        20        25        30
## 0.6066375 0.5712667 0.5875750 0.5876125 0.5886167 0.5724042
##        35        40        45        50        55        60
## 0.6254333 0.5805417 0.5378833 0.5309792 0.5124042 0.5018667
##        65
## 0.4697833
##
## $`Number of PCs Achieving Highest Mean Success`
## [1] "35"
##
## $`Root Mean Squared Error by Number of PCs of PCA`
##         5        10        15        20        25        30
## 0.4311594 0.4644015 0.4489683 0.4500623 0.4481304 0.4648665
##        35        40        45        50        55        60
## 0.4159569 0.4576886 0.4957241 0.5028386 0.5203736 0.5299541
##        65
## 0.5611892
##
## $`Number of PCs Achieving Lowest MSE`
## [1] "35"
##
## $`Median and Confidence Interval for Random Chance`
##      2.5%       50%     97.5%
## 0.3770383 0.4968641 0.6027875
##
## $`Mean Successful Assignment by Number of PCs of PCA`
##        20        21        22        23        24        25
## 0.5822583 0.5811958 0.5721250 0.5551000 0.5868667 0.5853542
##        26        27        28        29        30        31
## 0.5743792 0.5859250 0.5847333 0.5725000 0.5720958 0.5883458
##        32        33        34        35        36        37
## 0.5822042 0.5723500 0.6207208 0.6244875 0.6080125 0.6019042
##        38        39        40        41        42        43
## 0.5960417 0.5885042 0.5792958 0.5713958 0.5660500 0.5633250
##        44        45        46        47        48        49
## 0.5523625 0.5393083 0.5242625 0.5138542 0.5158375 0.5072458
##        50
## 0.5274708
##
## $`Number of PCs Achieving Highest Mean Success`
## [1] "35"
##
## $`Root Mean Squared Error by Number of PCs of PCA`
##        20        21        22        23        24        25
## 0.4547523 0.4566452 0.4652833 0.4795908 0.4494657 0.4515115
##        26        27        28        29        30        31
## 0.4626394 0.4509916 0.4526933 0.4634341 0.4637957 0.4482239
##        32        33        34        35        36        37
## 0.4560300 0.4646597 0.4203737 0.4166548 0.4303424 0.4374831
##        38        39        40        41        42        43
## 0.4430701 0.4501946 0.4587894 0.4667825 0.4709701 0.4733773
##        44        45        46        47        48        49
## 0.4834204 0.4948913 0.5093612 0.5173998 0.5161632 0.5239359
##        50
## 0.5071432
##
## $`Number of PCs Achieving Lowest MSE`
## [1] "35"
```

5.10.1.2 Barplot

Group individuals according to DAPC posterior membership.
carefull: the strong priors can sometimes assign random noise to the predefined structure

```
dapc <- xval$DAPC
colour <- funky(2)
post <- as.data.frame(dapc$posterior)
barplot(t(as.matrix(post)), col = colour, xlab = "Individual",
    ylab = "Assignment", main = paste0("Location prior: K=2 & PC=",
        PC, sep = ""), border = NA, ylim = c(0, 1.2), names.arg = row.names(post),
    las = 2, cex.names = 0.5, cex.lab = 1.5, cex.axis = 1.5)
```

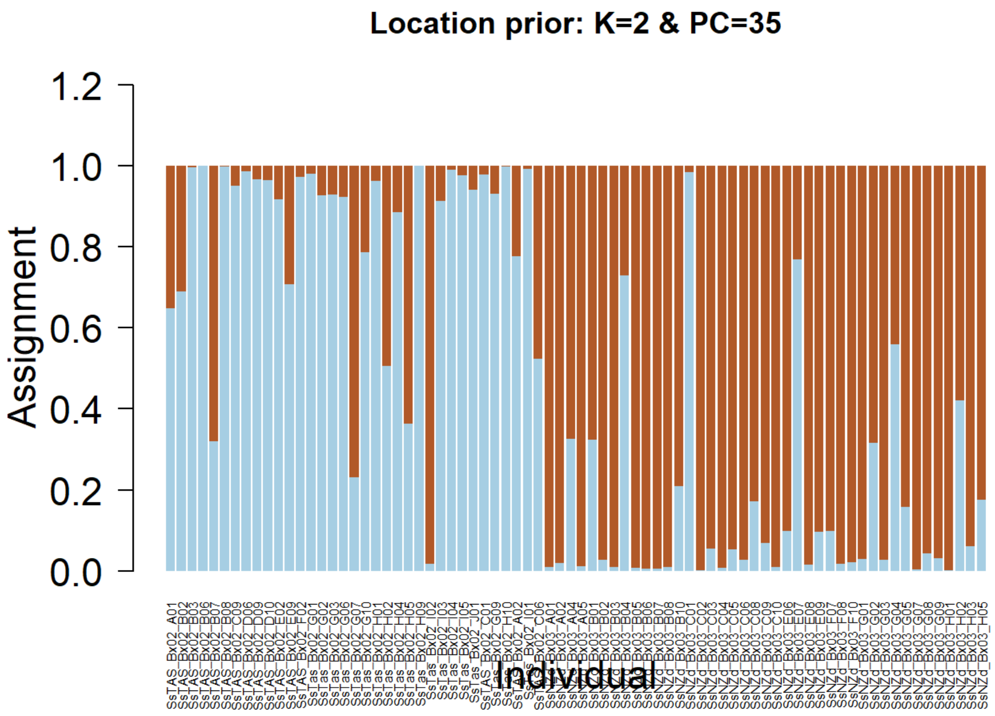

5.10.2 DAPC without location prior

The find.clusters function will give a PRIOR assignment according to the data to run DAPC.

5.10.2.1 Optimal number of clusters

```
# set.seed(124) grp <- adegenet::find.clusters(gi,
# max.n.clust = 20, n.pca = nInd(gl)/3, stat='BIC') save(grp,
# file = ''NeoFS_K2_DAPC2.rdata')
load("NeoFS_K2_DAPC2.rdata")

y <- as.numeric(grp$Kstat)
x <- 1:35
data <- data.frame(x, y, stringsAsFactors = F)
plot <- ggplot2::ggplot(data, aes(x, y)) + geom_point(size = 5,
    shape = 1, color = "blue") + geom_line(size = 1, color = "blue") +
    scale_x_continuous(name = waiver(), breaks = seq(from = 0,
        to = nrow(gl) - 1, by = 5)) + labs(subtitle = "", y = "Bayesian Information Criterion",
    x = "Number of clusters", title = "", caption = "") + theme_classic() +
    theme(axis.text = element_text(size = 15), axis.title.x = element_text(size = 20,
        vjust = 0, hjust = 0.5), axis.title.y = element_text(size = 20,
        vjust = 2, hjust = 0.5))
print(plot)
```

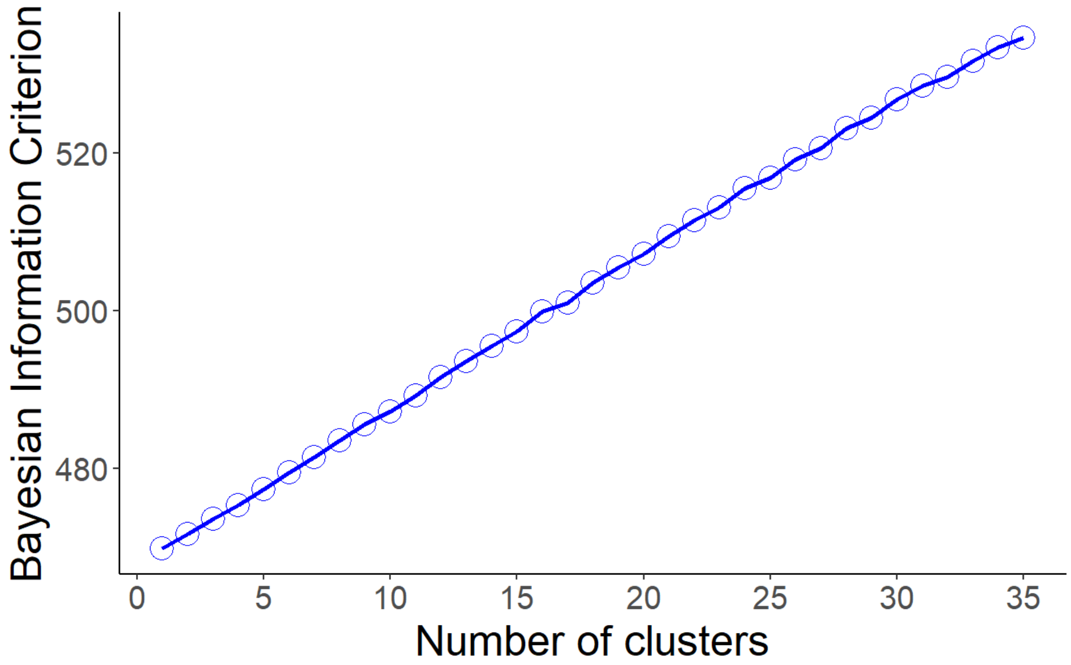

5.10.2.2 Barplot

Group individuals according to DAPC posterior membership.

```
for (K in 2:9) {
    set.seed(124)
    grp <- adegenet::find.clusters(gl, n.clust = K, n.pca = PC)
    set.seed(124)
    dapc <- adegenet::dapc(gl, grp$grp, n.da = K - 1, n.pca = 5)
    colour <- funky(K)
    post <- as.data.frame(dapc$posterior)
    colnames(post) <- paste("Group", 1:nlevels(dapc$grp))
    barplot(t(as.matrix(post)), col = colour, xlab = "", ylab = "Assignment",
        main = paste0("K=", K, " & PC=", PC, sep = ""), border = NA,
        ylim = c(0, 1.2), names.arg = row.names(post), las = 2,
        cex.names = 0.5, cex.lab = 1.5, cex.axis = 1.5)
}
```

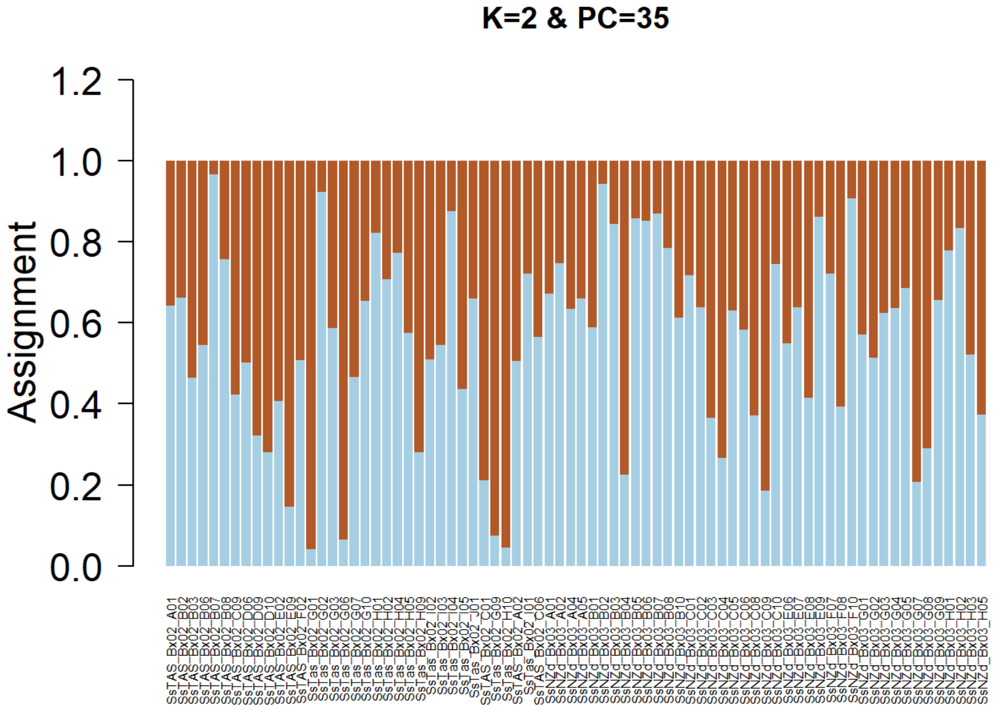
K=2 & PC=35
Assignment
1.2
1.0
0.8
0.6
0.4
0.2
0.0

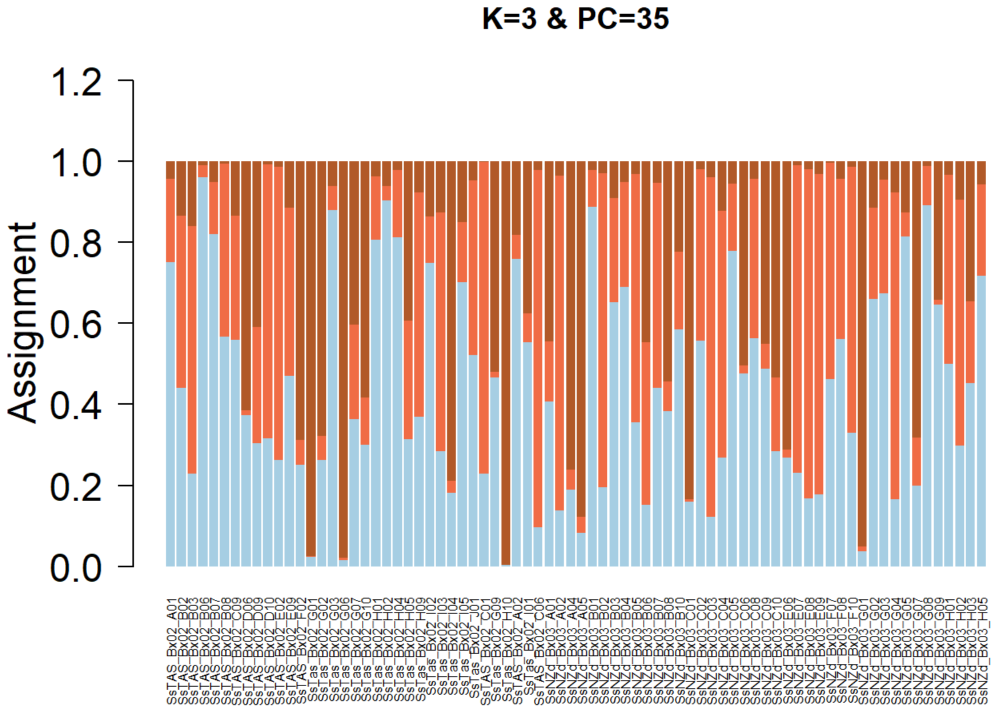
K=3 & PC=35
Assignment
1.2
1.0
0.8
0.6
0.4
0.2
0.0

K=4 & PC=35

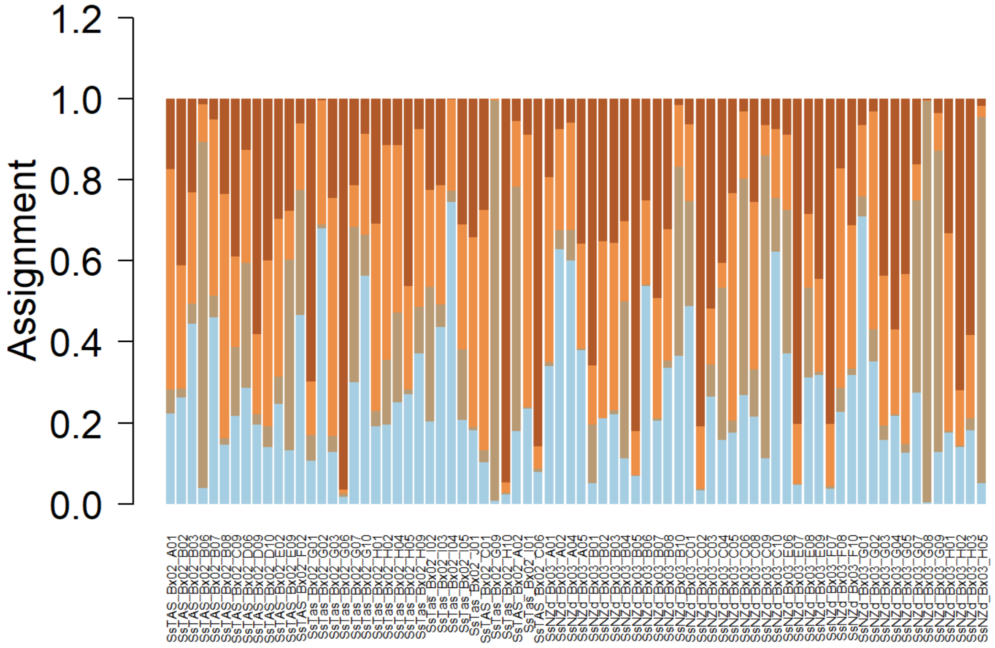

K=5 & PC=35

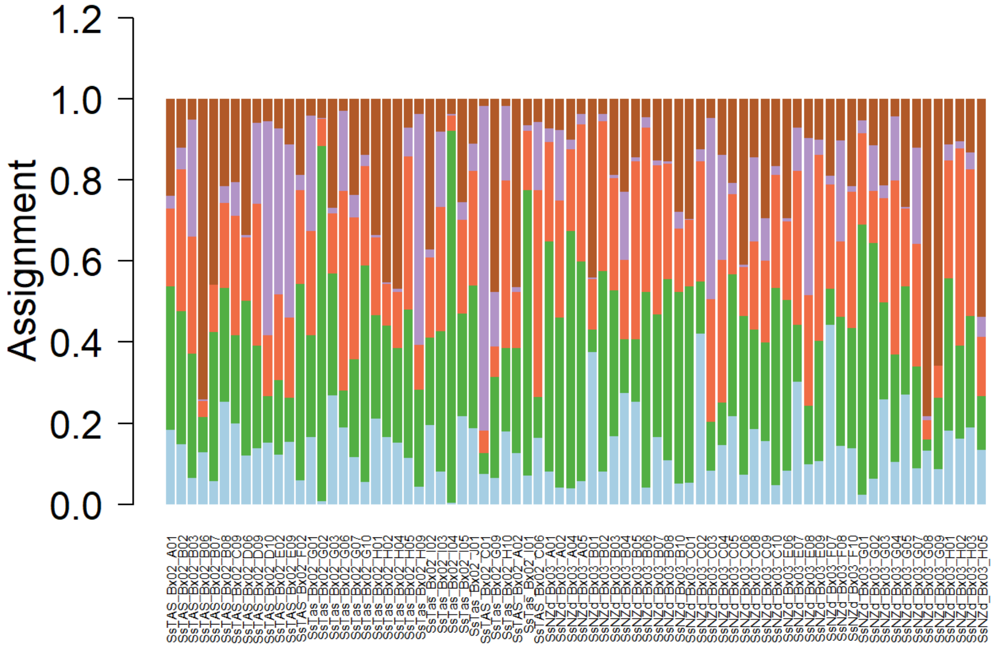

K=6 & PC=35

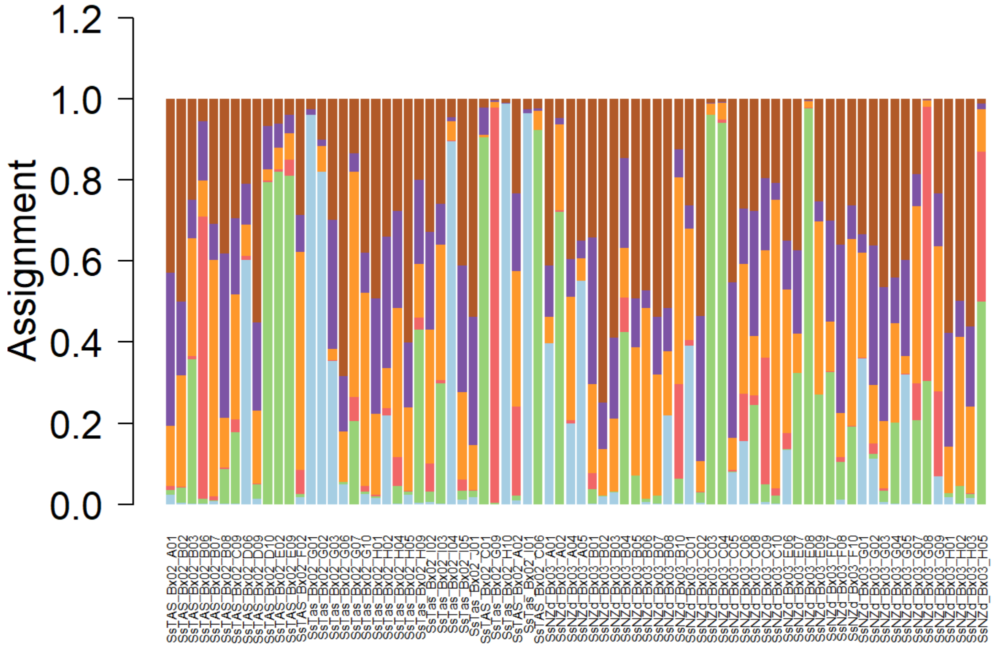

K=7 & PC=35

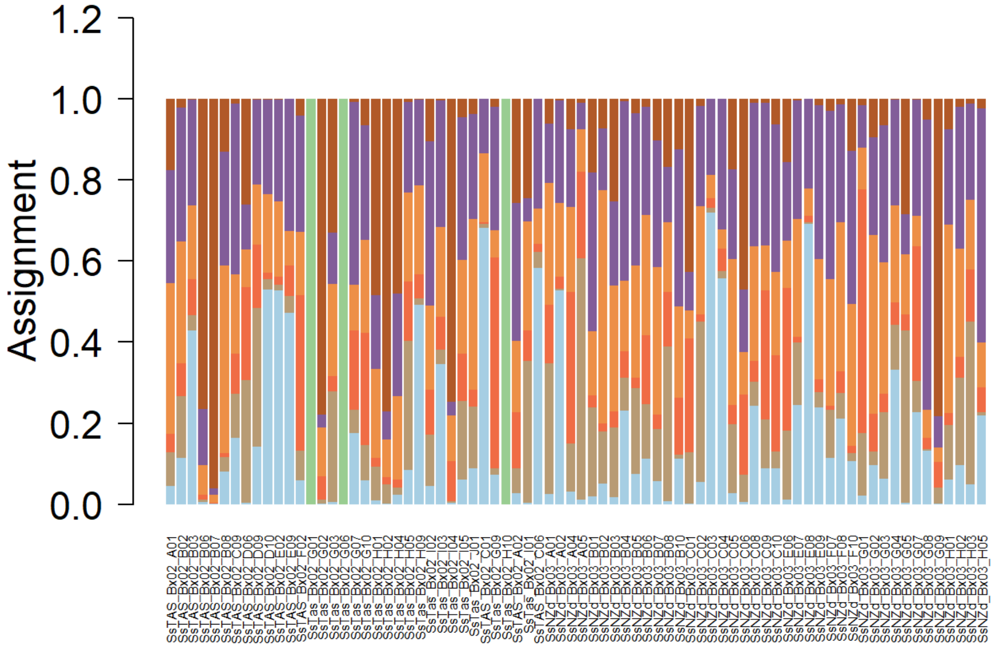

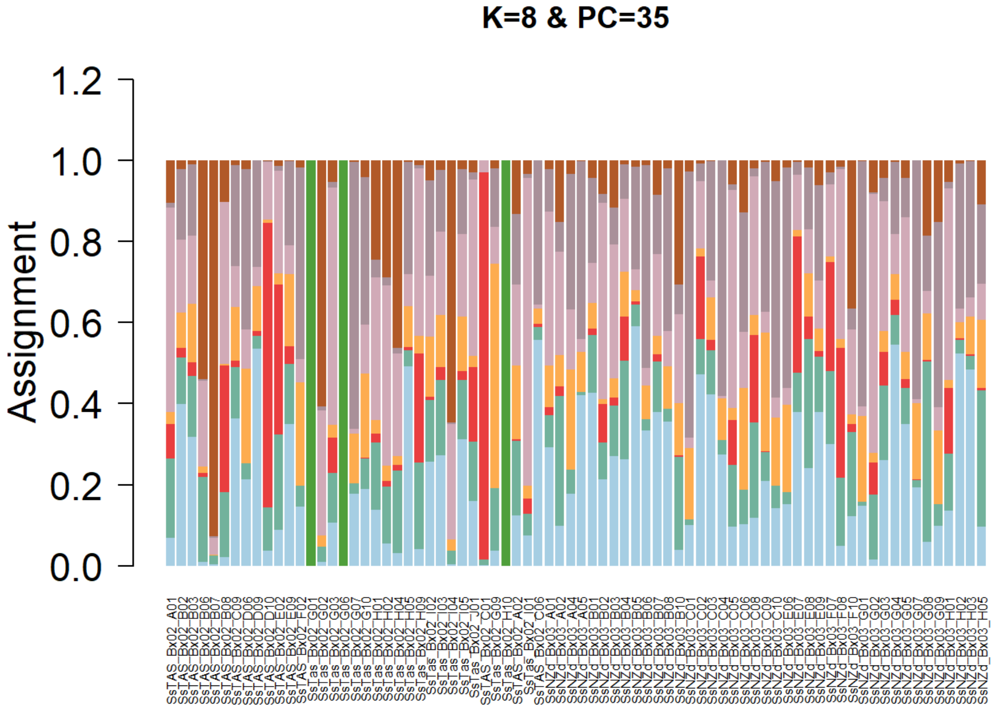

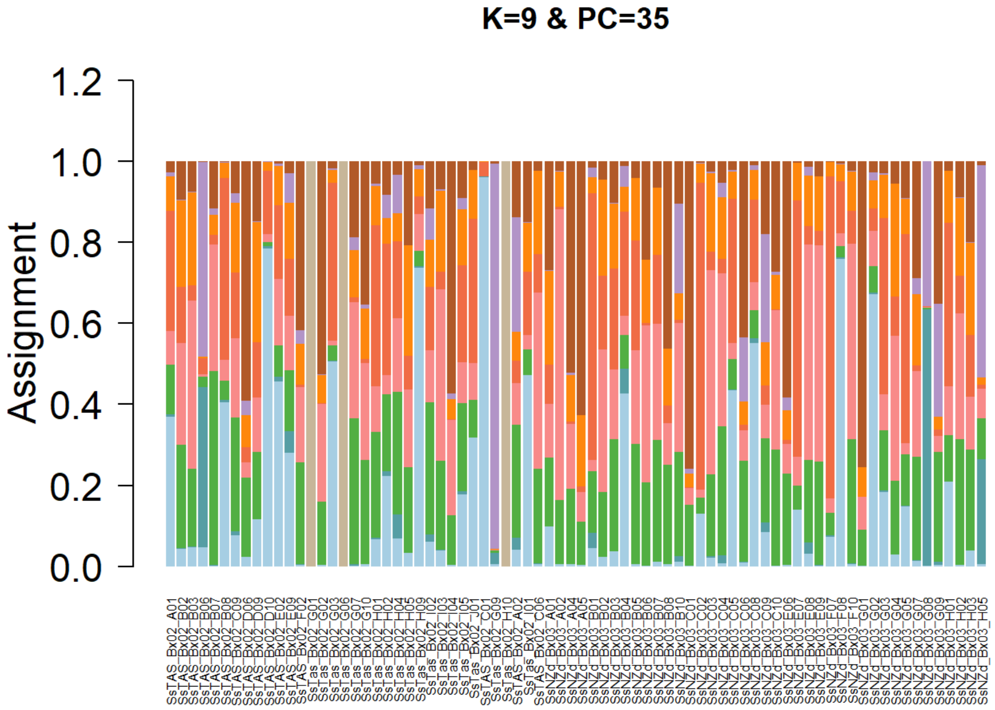

5.11 STRUCTURE output

```r
for (k in 2:9) {
    colour <- funky(k)
    tbl <- read.table(paste0("./STRUCTURE/Neo_noFS2/K", k, "/K",
        k, "outfile1_q", sep = ""))
    for (n in 2:20) {
        tmp <- read.table(paste0("./STRUCTURE/Neo_noFS2/K", k,
            "/K", k, "outfile", n, "_q", sep = ""))
        tmp2 <- tmp[, -c(1, 2)]
        tbl2 <- tbl[, -c(1, 2)]
        out <- cbind(tmp2, tbl2)
        Means <- sapply(unique(colnames(out)), function(i) rowMeans(out[,
            colnames(out) == i]))
        tbl <- data.frame(V1 = tbl$V1, V2 = tbl$V2, Means)
    }
    names <- tbl$V1
    tbl$V1 <- NULL
    tbl$V2 <- NULL

    suppressWarnings(barplot(t(as.matrix(tbl)), col = colour,
        xlab = "", ylab = "Ancestry", main = paste0("K=", k,
            sep = ""), border = NA, ylim = c(0, 1.2), names.arg = names,
        las = 2, cex.names = 0.5, cex.lab = 1.5, cex.axis = 1.5))
}
```

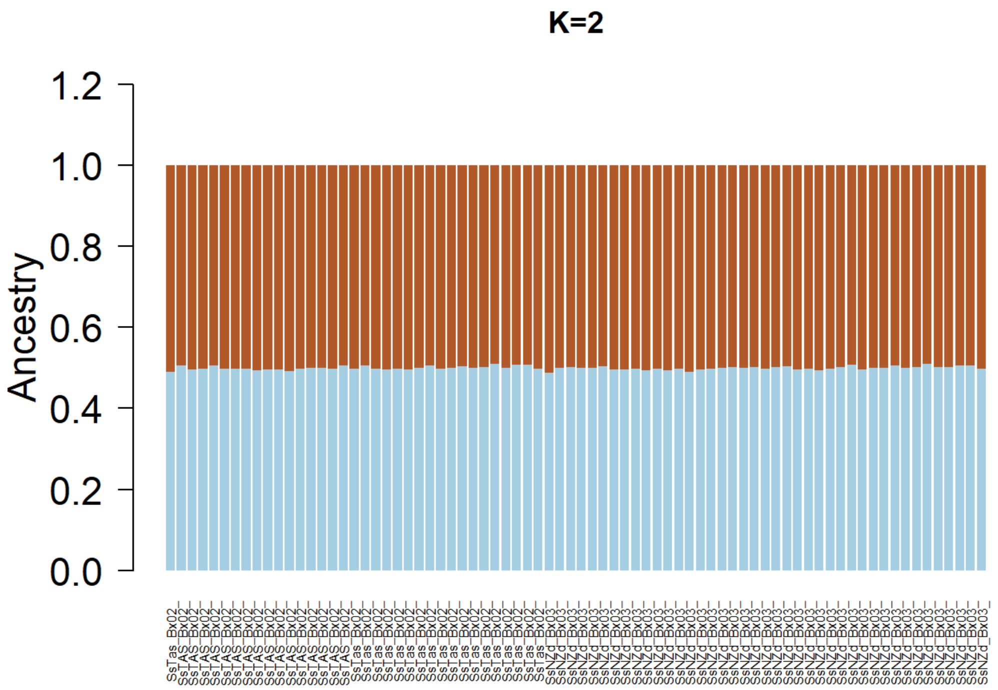

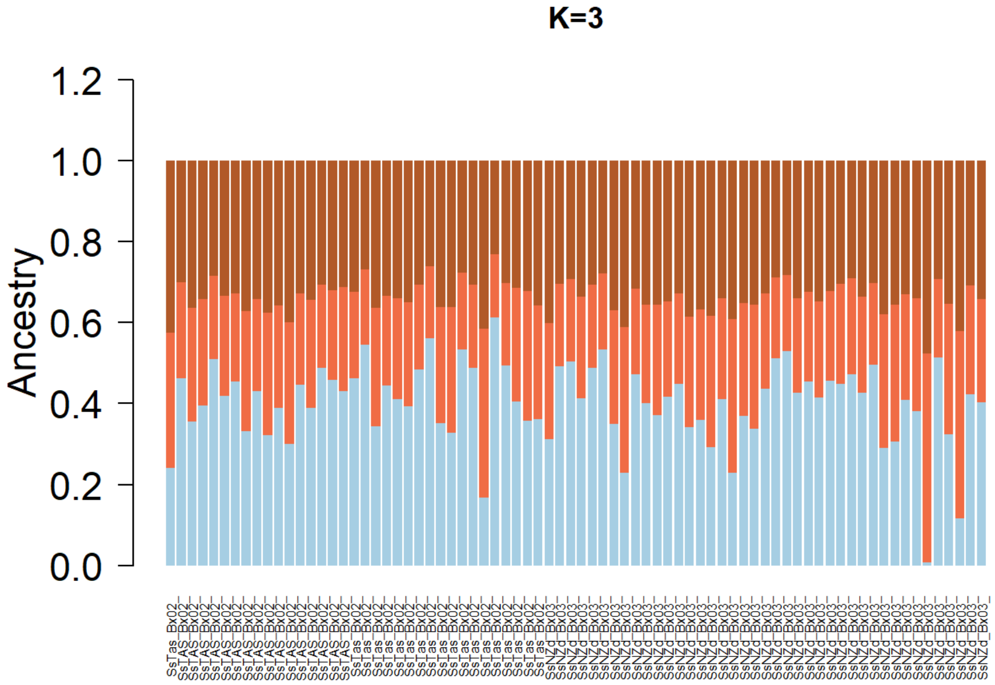
K=3
1.2
1.0
0.8
0.6
0.4
0.2
0.0
Ancestry

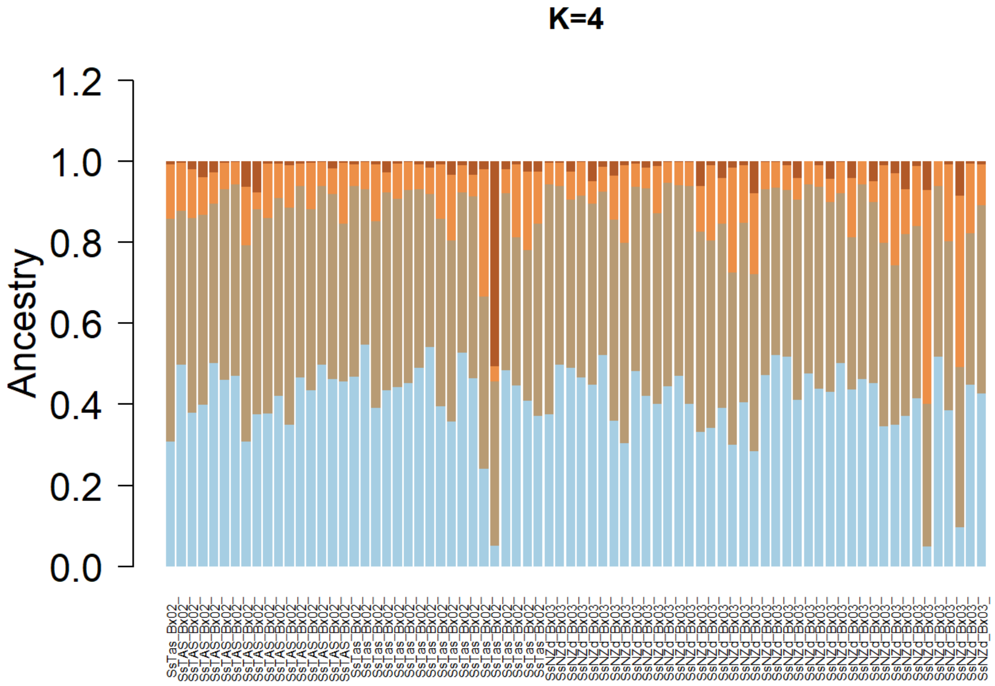
K=4
1.2
1.0
0.8
0.6
0.4
0.2
0.0
Ancestry

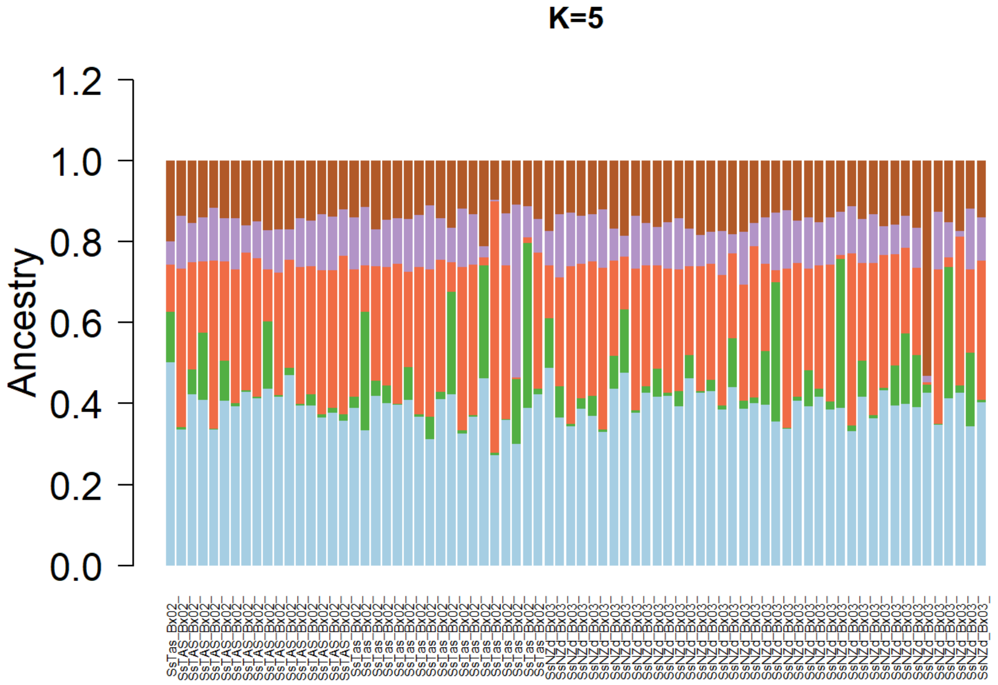
K=5
1.2
1.0
0.8
0.6
0.4
0.2
0.0
Ancestry

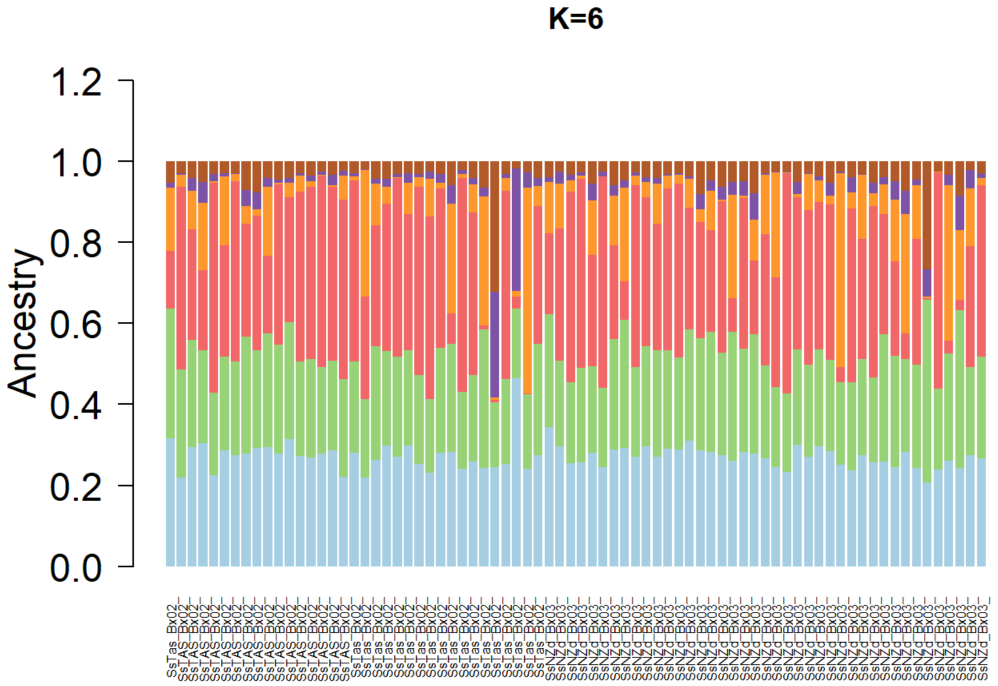
K=6
1.2
1.0
0.8
0.6
0.4
0.2
0.0
Ancestry

K=7

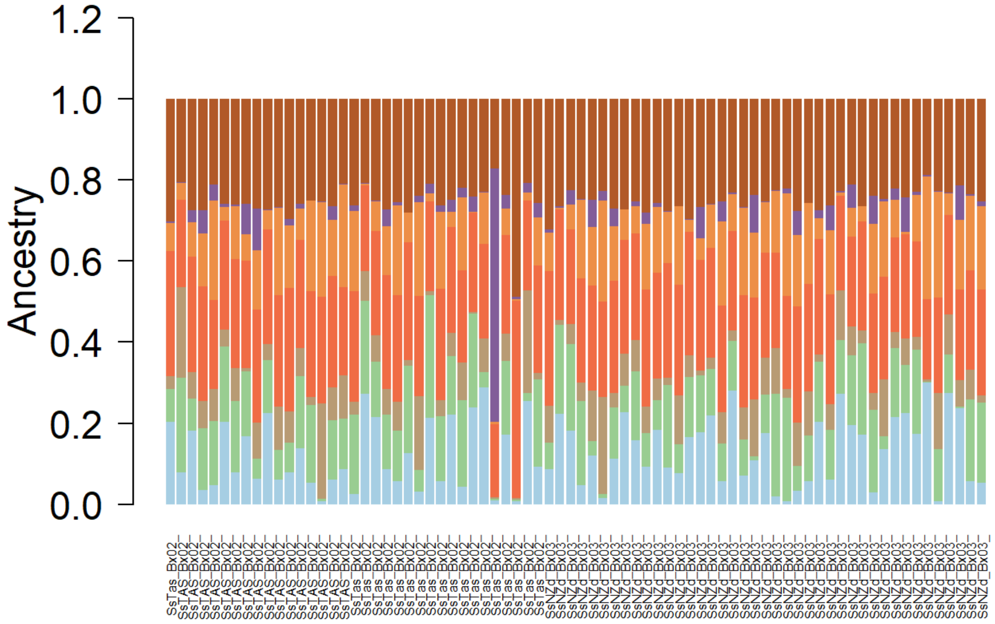

K=8

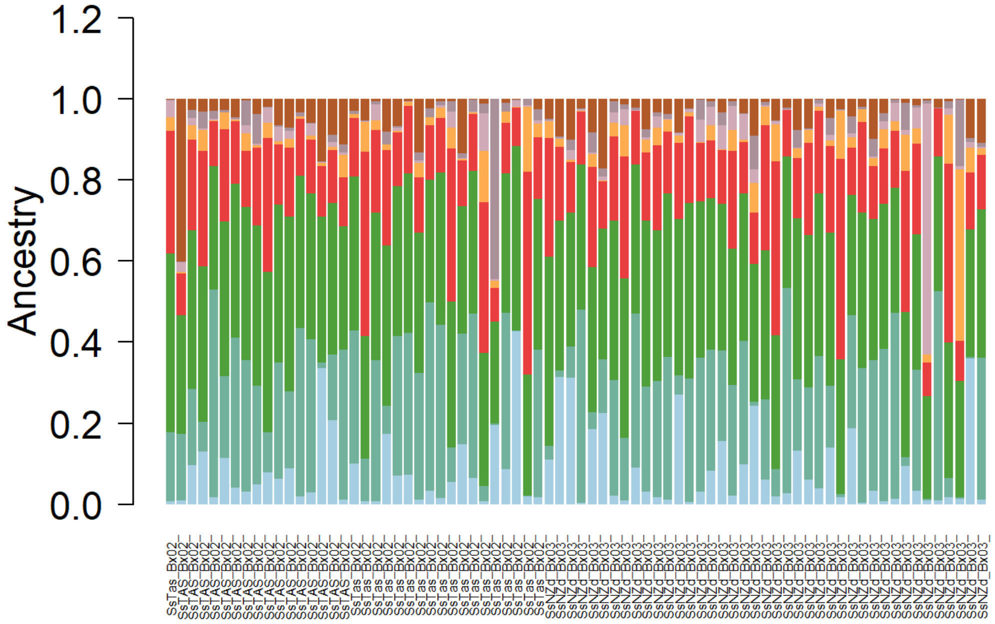

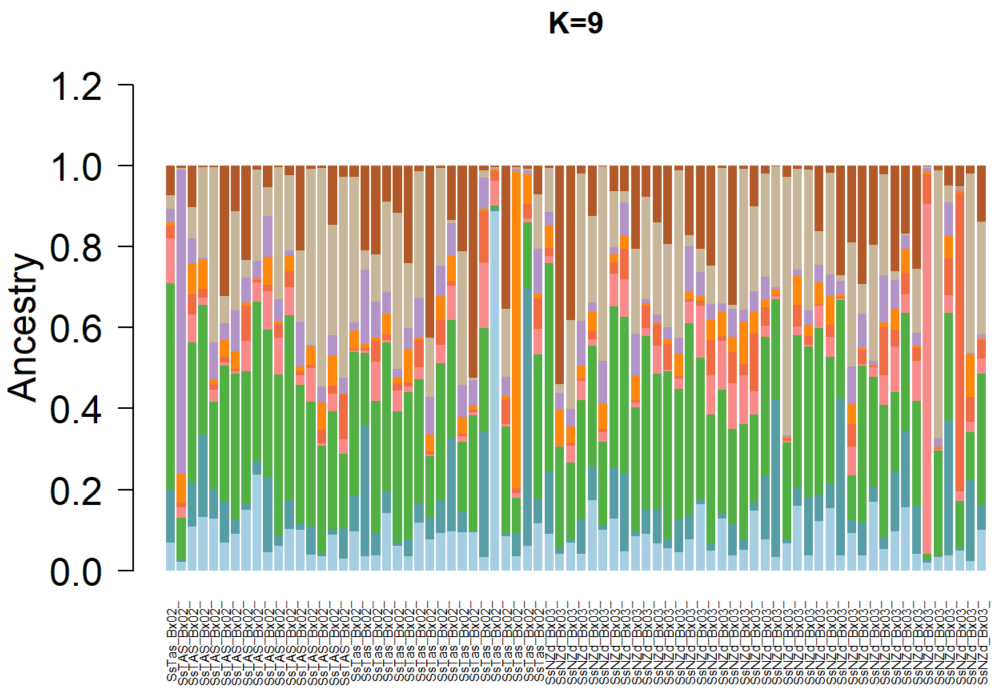

5.12 Assignment test

```
# gpop <- assignPOP::read.Genepop('SS_genepop.txt', pop.names
# = c('TAS', 'NZ'), haploid = FALSE, pos = 1)
# assignPOP::assign.MC(gpop, train.inds = c(0.1, 0.5, 0.9),
# train.loci = c(0.1, 0.5, 1), loci.sample = 'fst',
# iterations = 100, dir = 'AssignPOP_MC/', scaled = FALSE,
# pca.method = 'mixed', pca.PCs = 'kaiser-guttman',
# pca.loadings = F, model = 'svm', svm.kernel = 'linear',
# svm.cost = 1, ntree = 50, multiprocess = TRUE, skipQ =
# FALSE)
df1 <- assignPOP::accuracy.MC(dir = "AssignPOP_MC/")
```

```
##
##   Correct assignment rates were estimated!!
##   A total of 900 assignment tests for 2 pops.
##   Results were also saved in a 'Rate_of_900_tests_2_pops.txt' file in the directory.
```

```
MC.assign <- assignPOP::accuracy.plot(df1, pop = "all")
print(MC.assign)
```

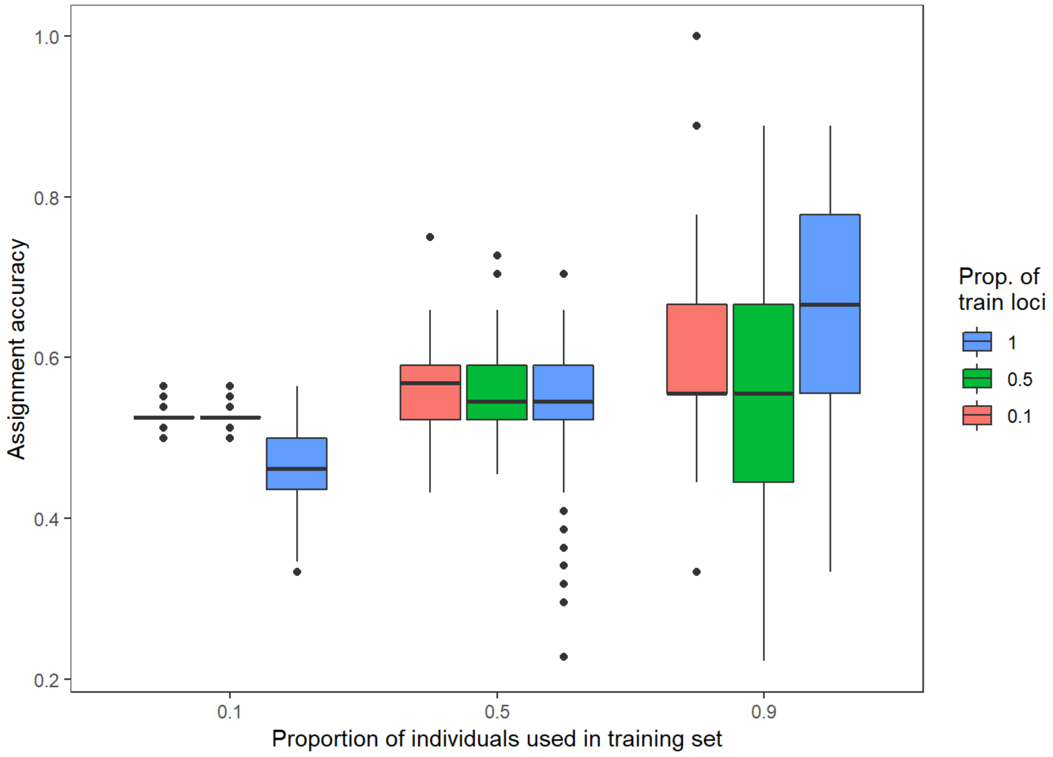


```
# assignPOP::assign.kfold(gpop, k.fold = 10, train.loci = 1,
# loci.sample = 'fst', dir = 'AssignPOP_Kfold/', scaled =
# FALSE, pca.method = 'mixed', pca.PCs = 'kaiser-guttman',
# pca.loadings = F, model = 'svm', svm.kernel = 'linear',
# svm.cost = 1, ntree = 50, multiprocess = TRUE, skipQ =
# FALSE)
df2 <- assignPOP::accuracy.kfold(dir = "AssignPOP_Kfold/")
```

```
##
##   Correct assignment rates were estimated!!
##   A total of 10 assignment tests for 2 pops.
##   Results were also saved in a 'Rate_of_10_tests_2_pops.txt' file in the directory.
```

```
kfold.assign <- assignPOP::accuracy.plot(df2, pop = c("all",
    "TAS", "NZ"))
print(kfold.assign)
```

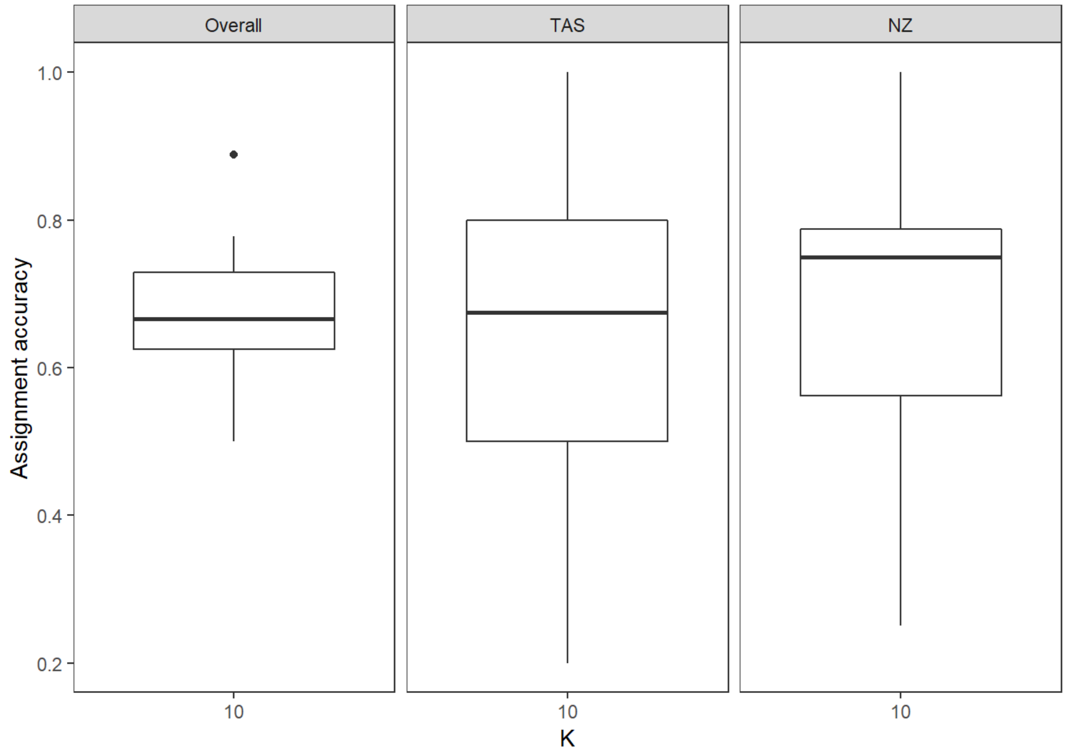


```r
assignPOP::membership.plot(dir = "AssignPOP_Kfold/", style = 1,
    non.genetic = TRUE)
```

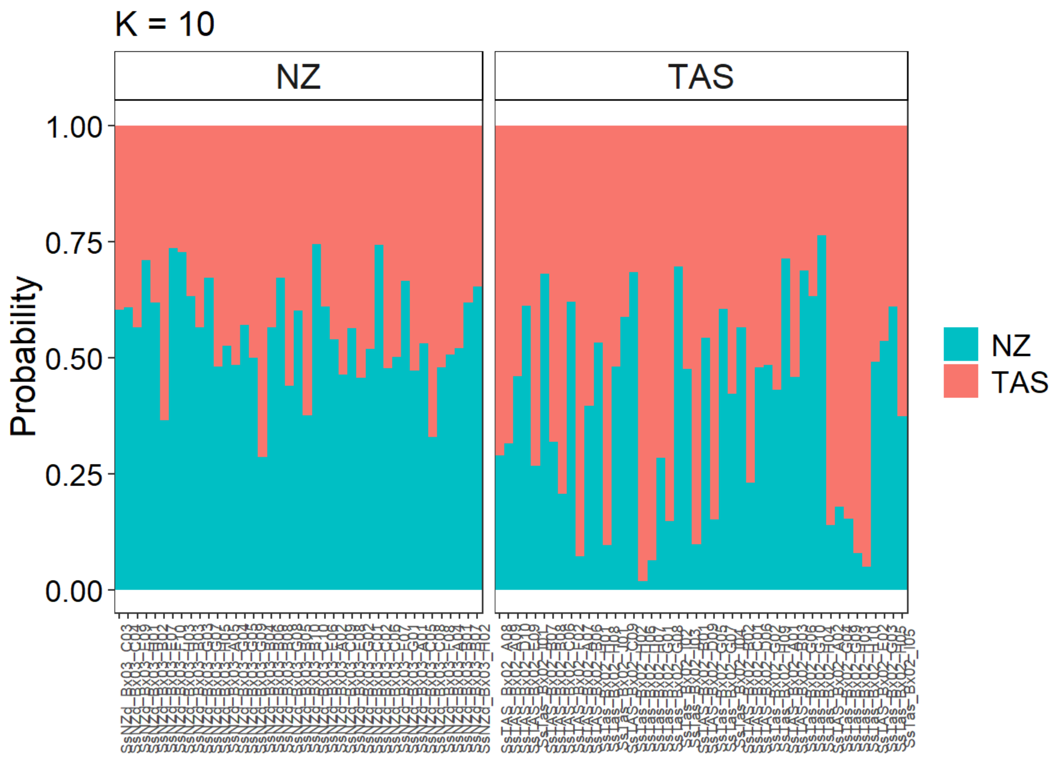

5.13 Sex-biased gene flow

```r
set.seed(124)
glmat <- as.matrix(gl)
glmat[glmat == 0] <- 303
glmat[glmat == 1] <- 304
glmat[glmat == 2] <- 404
dat <- data.frame(population = as.integer(gl$pop), glmat[])
row.names(dat) <- gl$ind.names
sex <- as.character(gl$other$ind.metrics$Sex2)
sexes <- names(table(sex))

tests <- c("mAIc", "vAIc", "FIS", "FST")
SBD <- list()
for (i in tests) {
    SBD[[i]] <- hierfstat::sexbias.test(dat, sex, nperm = 10,
        test = i, alternative = "two.sided")
}
SBD$mAIc
```

```
## $call
## hierfstat::sexbias.test(dat = dat, sex = sex, nperm = 10, test = i,
##     alternative = "two.sided")
##
## $statistic
## [1] -0.06705428
##
## $p.value
## [1] 1
```

```r
SBD$vAIc
```

```
## $call
## hierfstat::sexbias.test(dat = dat, sex = sex, nperm = 10, test = i,
##     alternative = "two.sided")
##
## $statistic
## [1] 0.9111
##
## $p.value
## [1] 0.8
```

```r
SBD$FIS
```

```
## $call
## hierfstat::sexbias.test(dat = dat, sex = sex, nperm = 10, test = i,
##     alternative = "two.sided")
##
## $statistic
## [1] -0.0007884171
##
## $p.value
## [1] 0.9
```

```r
SBD$FST
```

```
## $call
## hierfstat::sexbias.test(dat = dat, sex = sex, nperm = 10, test = i,
##     alternative = "two.sided")
##
## $statistic
## [1] -0.00139475
##
## $p.value
## [1] 0.3
```

```
# Check differentiation between sexes
gl$pop <- as.factor(gl$other$ind.metrics$Sex2)
pwfst <- StAMPP::stamppFst(gl, nboots = boots, percent = 95,
    nclusters = 3)
Fst <- c(pwfst$Bootstraps$Fst, pwfst$Bootstraps$`p-value`, pwfst$Bootstraps$`Lower bound CI limit`,
    pwfst$Bootstraps$`Upper bound CI limit`)
names(Fst) <- c("Fst", "P-value", "Lower CI", "Upper CI")
print(Fst)
```

```
##           Fst       P-value      Lower CI      Upper CI
##  8.427892e-05  3.910000e-01 -4.662390e-04  6.306888e-04
```

```
bs <- mmod::chao_bootstrap(gi, nreps = boots2)
bs.D <- mmod::summarise_bootstrap(bs, D_Jost)
```

```
## Warning in mmod::summarise_bootstrap(bs, D_Jost): Bootstrap
## distribution of D_Jost includes negative values, harmonic
## mean is undefined
```

```
print(bs.D$summary.global.het)
```

```
##         observed     lower.normal     upper.normal
##     0.0005934471     0.0002213704     0.0009655238
##          std.dev             mean lower.percentile
##     0.0001898351     0.0060211039     0.0056094649
## upper.precentile
##     0.0063907513
```

```
nc.diff_stats <- mmod::diff_stats(gi, phi_st = TRUE)
print(nc.diff_stats$global)
```

```
##           Hs           Ht      Gst_est    Gprime_st
## 0.2945574980 0.2947668194 0.0007101254 0.0020118479
##        D_het       D_mean       Phi_st
## 0.0005934471           NA 0.0010201417
```

```
with(nc.diff_stats, pairs(per.locus[, 3:6], upper.panel = panel.smooth))
```

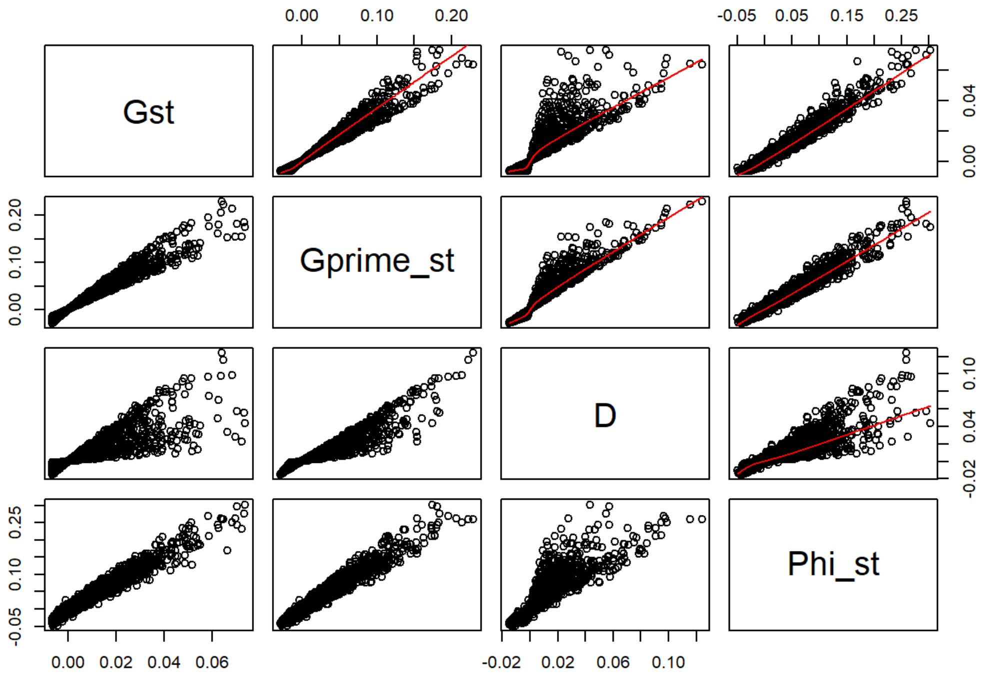

6 Citations for packages

```
packages <- c("knitr", "adegenet", "SNPRelate", "dartR", "pegas",
    "ape", "hierfstat", "plyr", "diveRsity", "StAMPP", "mmod",
    "ggplot2", "data.table", "vioplot", "assignPOP", "OutFLANK",
    "qvalue", "pcadapt", "base", "rmarkdown")
for (P in packages) {
    p <- citation(P)
    print(attr(unclass(p)[[1]], "textVersion"))
}
```

```
## [1] "Yihui Xie (2018). knitr: A General-Purpose Package for Dynamic Report Generation in R. R pac
kage version 1.21."
## [1] "Jombart, T. (2008) adegenet: a R package for the multivariate analysis of genetic markers. B
ioinformatics 24: 1403-1405. doi: 10.1093/bioinformatics/btn129"
## [1] "Xiuwen Zheng, David Levine, Jess Shen, Stephanie M. Gogarten, Cathy Laurie, Bruce S. Weir. A
High-performance Computing Toolset for Relatedness and Principal Component Analysis of SNP Data. Bio
informatics 2012; doi: 10.1093/bioinformatics/bts606"
## [1] "Bernd Gruber and Arthur Georges (2018). dartR: Importing and Analysing SNP and Silicodart Da
ta Generated by\nGenome-Wide Restriction Fragment Analysis. R package version 1.1.6. https://CRAN.R-
project.org/package=dartR"
## [1] "Paradis E. 2010. pegas: an R package for population genetics with an integrated-modular appr
oach. Bioinformatics 26: 419-420."
## [1] "Paradis E. & Schliep K. 2018. ape 5.0: an environment for modern phylogenetics and evolution
ary analyses in R. Bioinformatics xx: xxx-xxx."
## [1] "Jerome Goudet and Thibaut Jombart (2015). hierfstat: Estimation and Tests of Hierarchical F-
Statistics. R package version 0.04-22. https://CRAN.R-project.org/package=hierfstat"
## [1] "Hadley Wickham (2011). The Split-Apply-Combine Strategy for Data Analysis. Journal of Statis
tical Software, 40(1), 1-29. URL http://www.jstatsoft.org/v40/i01/."
## [1] "Keenan, K., McGinnity, P., Cross, T.F., Crozier, W.W., & Prodöhl, P.A., (2013), diveRsity: A
n R package for the estimation of population genetics parameters and their associated errors,
Methods in Ecology and Evolution, doi: 10.1111/2041-210X.12067"
## [1] "Pembleton LW, Cogan NOI, Forster JW (2013) StAMPP: an R package for calculation of genetic d
ifferentiation and structure of mixed-ploidy level populations. Molecular Ecology Resources 13(5), 9
46-952"
## [1] "Winter D.J. (In press). mmod: an R library for the calculation of population differentiation
statistics"
## [1] "H. Wickham. ggplot2: Elegant Graphics for Data Analysis. Springer-Verlag New York, 2016."
## [1] "Matt Dowle and Arun Srinivasan (2018). data.table: Extension of `data.frame`. R package vers
ion 1.11.8. https://CRAN.R-project.org/package=data.table"
## [1] "Daniel Adler (2005). vioplot: Violin plot. R package version 0.2.
http://wsopuppenkiste.wiso.uni-goettingen.de/~dadler"
## [1] "Kuan-Yu Chen, Elizabeth A. Marschall, Michael G. Sovic, Anthony C. Fries, H. Lisle Gibbs and
Stuart A. Ludsin (2018). assignPOP: Population Assignment using Genetic, Non-Genetic or
Integrated\nData in a Machine Learning Framework. R package version 1.1.4. https://CRAN.R-
project.org/package=assignPOP"
## [1] "Michael C. Whitlock and Katie Lotterhos (2014). OutFLANK: Fst outliers with trimming. R pack
age version 0.2. "
## [1] "John D. Storey with contributions from Andrew J. Bass, Alan Dabney and David Robinson (2015)
. qvalue: Q-value estimation for false discovery rate control. R package version 2.12.0.
http://github.com/jdstorey/qvalue"
## [1] "Keurcien Luu, Michael Blum and Florian Privé (2018). pcadapt: Fast Principal Component Analy
sis for Outlier Detection. R package version 4.0.3. https://CRAN.R-project.org/package=pcadapt"
## [1] "R Core Team (2018). R: A language and environment for statistical computing. R Foundation fo
r Statistical Computing, Vienna, Austria. URL https://www.R-project.org/."
## [1] "JJ Allaire and Yihui Xie and Jonathan McPherson and Javier Luraschi and Kevin Ushey and Aron
Atkins and Hadley Wickham and Joe Cheng and Winston Chang and Richard Iannone (2018). rmarkdown: Dyn
amic Documents for R. R package version 1.11. URL https://rmarkdown.rstudio.com."
```

7 Session info

```
sessionInfo()
```

```
## R version 3.5.1 (2018-07-02)
## Platform: x86_64-w64-mingw32/x64 (64-bit)
## Running under: Windows 10 x64 (build 16299)
##
## Matrix products: default
##
## locale:
## [1] LC_COLLATE=English_Australia.1252
## [2] LC_CTYPE=English_Australia.1252
## [3] LC_MONETARY=English_Australia.1252
## [4] LC_NUMERIC=C
```

```
## [5] LC_TIME=English_Australia.1252
##
## attached base packages:
## [1] stats     graphics  grDevices utils     datasets
## [6] methods   base
##
## other attached packages:
##  [1] pcadapt_4.0.3     OutFLANK_0.2      qvalue_2.12.0
##  [4] vioplot_0.2       sm_2.2-5.6        data.table_1.11.8
##  [7] ggplot2_3.1.0     mmod_1.3.3        StAMPP_1.5.1
## [10] diveRsity_1.9.90  plyr_1.8.4        hierfstat_0.04-22
## [13] assigner_0.5.2    pegas_0.11        ape_5.2
## [16] dartR_1.1.6       SNPRelate_1.14.0  gdsfmt_1.16.0
## [19] adegenet_2.1.1    ade4_1.7-13       formatR_1.5
## [22] knitr_1.21
##
## loaded via a namespace (and not attached):
##   [1] R.utils_2.7.0           tidyselect_0.2.5
##   [3] lme4_1.1-19             htmlwidgets_1.3
##   [5] grid_3.5.1              combinat_0.0-8
##   [7] munsell_0.5.0           codetools_0.2-15
##   [9] future_1.10.0           miniUI_0.1.1.1
##  [11] withr_2.1.2             colorspace_1.3-2
##  [13] fst_0.8.10              rstudioapi_0.8
##  [15] stats4_3.5.1            pbmcapply_1.3.0
##  [17] listenv_0.7.0           labeling_0.3
##  [19] huge_1.2.7              mi_1.0
##  [21] RgoogleMaps_1.4.3       mnormt_1.5-5
##  [23] generics_0.0.2          coda_0.19-2
##  [25] LearnBayes_2.15.1       ipred_0.9-8
##  [27] xfun_0.4                randomForest_4.6-14
##  [29] R6_2.3.0                doParallel_1.0.14
##  [31] arm_1.10-1              manipulateWidget_0.10.0
##  [33] reshape_0.8.8           assertthat_0.2.0
##  [35] promises_1.0.1          scales_1.0.0
##  [37] pinfsc50_1.1.0          nnet_7.3-12
##  [39] gtable_0.2.0            globals_0.12.4
##  [41] timeDate_3043.102       rlang_0.3.0.1
##  [43] calibrate_1.7.2         splines_3.5.1
##  [45] rgdal_1.3-6             lazyeval_0.2.1
##  [47] ModelMetrics_1.2.2      acepack_1.4.1
##  [49] checkmate_1.8.5         rgl_0.99.16
##  [51] yaml_2.2.0              reshape2_1.4.3
##  [53] abind_1.4-5             d3Network_0.5.2.1
##  [55] crosstalk_1.0.0         backports_1.1.3
##  [57] httpuv_1.4.5.1          Hmisc_4.1-1
##  [59] caret_6.0-81            tree_1.0-39
##  [61] lava_1.6.4              tools_3.5.1
##  [63] tcltk_3.5.1             psych_1.8.10
##  [65] lavaan_0.6-3            spData_0.2.9.6
##  [67] ggm_2.3                 statnet.common_4.1.4
##  [69] raster_2.8-4            RColorBrewer_1.1-2
##  [71] Rcpp_1.0.0              rrBLUP_4.6
##  [73] radiator_0.0.13         base64enc_0.1-3
##  [75] purrr_0.2.5             rpart_4.1-13
##  [77] deldir_0.1-15           pbapply_1.3-4
##  [79] qgraph_1.5              cluster_2.0.7-1
##  [81] magrittr_1.5            sna_2.4
##  [83] genetics_1.3.8.1        gmodels_2.18.1
##  [85] mvtnorm_1.0-8           matrixcalc_1.0-3
##  [87] whisker_0.3-2           amap_0.8-16
##  [89] hms_0.4.2               mime_0.6
##  [91] evaluate_0.12           xtable_1.8-3
##  [93] leaflet_2.0.2           jpeg_0.1-8
##  [95] gridExtra_2.3           vcfR_1.8.0
```

```
##  [97] compiler_3.5.1          ellipse_0.4.1
##  [99] tibble_1.4.2            crayon_1.3.4
## [101] minqa_1.2.4             gdistance_1.2-2
## [103] R.oo_1.22.0             htmltools_0.3.6
## [105] mgcv_1.8-24             corpcor_1.6.9
## [107] later_0.7.5             spdep_0.8-1
## [109] Formula_1.2-3           tidyr_0.8.2
## [111] expm_0.999-3            lubridate_1.7.4
## [113] PopGenReport_3.0.0      MASS_7.3-50
## [115] boot_1.3-20             Matrix_1.2-14
## [117] readr_1.3.1             permute_0.9-4
## [119] quadprog_1.5-5          R.methodsS3_1.7.1
## [121] gdata_2.18.0            gower_0.1.2
## [123] parallel_3.5.1          bindr_0.1.1
## [125] igraph_1.2.2            BDgraph_2.53
## [127] pkgconfig_2.0.2         sem_3.1-9
## [129] foreign_0.8-70          sp_1.3-1
## [131] recipes_0.1.4           plotly_4.8.0
## [133] foreach_1.4.4           pbivnorm_0.6.0
## [135] webshot_0.5.1           prodlim_2018.04.18
## [137] stringr_1.3.1           digest_0.6.18
## [139] vegan_2.5-3             assignPOP_1.1.4
## [141] rmarkdown_1.11          htmlTable_1.12
## [143] directlabels_2018.05.22 gap_1.1-22
## [145] shiny_1.2.0             gtools_3.8.1
## [147] rjson_0.2.20            nloptr_1.2.1
## [149] nlme_3.1-137            glasso_1.10
## [151] dismo_1.1-4             jsonlite_1.6
## [153] bindrcpp_0.2.2          network_1.13.0.1
## [155] seqinr_3.4-5            viridisLite_0.3.0
## [157] pillar_1.3.1            lattice_0.20-35
## [159] GGally_1.4.0            httr_1.4.0
## [161] survival_2.42-3         glue_1.3.0
## [163] fdrtool_1.2.15          png_0.1-7
## [165] iterators_1.0.10        class_7.3-14
## [167] stringi_1.2.4           pca3d_0.10
## [169] latticeExtra_0.6-28     dplyr_0.7.8
## [171] e1071_1.7-0
```
