# Supplementary material for: Accounting for kin sampling reveals genetic connectivity in Tasmanian and New Zealand school sharks, Galeorhinus galeus
Source: Ecol Evol. 2019 Apr 1;9(8):4465–72. doi: 10.1002/ece3.5012 (PMC6476751; doi:10.1002/ece3.5012)
Supplement: Supplementary file 2 [file ECE3-9-4465-s002.pdf]

Supporting Information

Accounting for kin sampling reveals genetic connectivity in Tasmanian and New Zealand school sharks, *Galeorhinus galeus*

Floriaan Devloo-Delva[1,2,], Gregory E. Maes[3,4,5], Sebastián I. Hernández[6,7], Jaime D. Mcallister[8], Rasanthi M. Gunasekera[1], Peter M. Grewe[1], Robin B. Thomson[1] and Pierre Feutry[1]*

[1] Oceans and Atmosphere, CSIRO, Hobart, 7000 TAS, Australia.
[2] School of Natural Sciences – Quantitative Marine Science, University of Tasmania, Hobart, 7001 TAS, Australia.
[3] Centre for Sustainable Tropical Fisheries and Aquaculture – Comparative Genomics Centre, College of Marine and Environmental Sciences, James Cook University, Townsville, 4811 QLD, Australia.
[4] Laboratory of Biodiversity and Evolutionary Genomics, KU Leuven, B-3000 Leuven, Belgium.
[5] Center for Human Genetics, UZ Leuven – Genomics Core, KU Leuven, B-3000 Leuven, Belgium.
[6] Biomolecular lab, Center for International Program, Universidad Veritas, Costa Rica.
[7] Sala de Colecciones, Facultad de Ciencias del Mar, Universidad Católica del Norte, Coquimbo, Chile.
[8] Fisheries and Aquaculture Centre, Institute for Marine and Antarctic Studies, University of Tasmania, Hobart, 7001 TAS, Australia.
*Email: Floriaan.Devloo-Delva@csiro.au

Content

- S1. Full analysis in Rmarkdown *(separate document)*
- S2. COLONY2 settings for school sharks analyzed in the present study and in Bester-van der Merwe et al. (2017)
- S3. STRUCTURE results for the dataset with full siblings included
- S4. STRUCTURE results for the dataset with full siblings excluded
- S5. Kinship results of COLONY2 for nine Tasmanian and 20 New Zealand school sharks from Bester-van der Merwe et al. (2017)

S2. COLONY2 settings for school sharks analyzed in the present study and in Bester-van der Merwe et al. *(2017)*

- Mating System – I: Female Polygamy/ Male Polygamy
- Mating System – II: With Inbreeding/ Without Clone
- Species: Dioecious/Diploid
- Length of Run: Medium
- Analysis Method: Full-Likelihood
- Likelihood Precision: Medium
- Run Specifications: Update Allele Frequency -> YES/Sibship Scaling -> YES/Number of Run -> 5
- Sibship Prior: Weak Prior/ Paternal Sibship Size -> 1/ Maternal Sibship Size -> 1

S3. STRUCTURE results for the dataset with full siblings included, n = 87 & 6,760 SNPs. A) Optimal number of clusters selection, based on the mean estimated ln probability for K ranging from one to nine. B) STRUCTURE ancestry plot between Tasmania and New Zealand, based on K = 8.

A

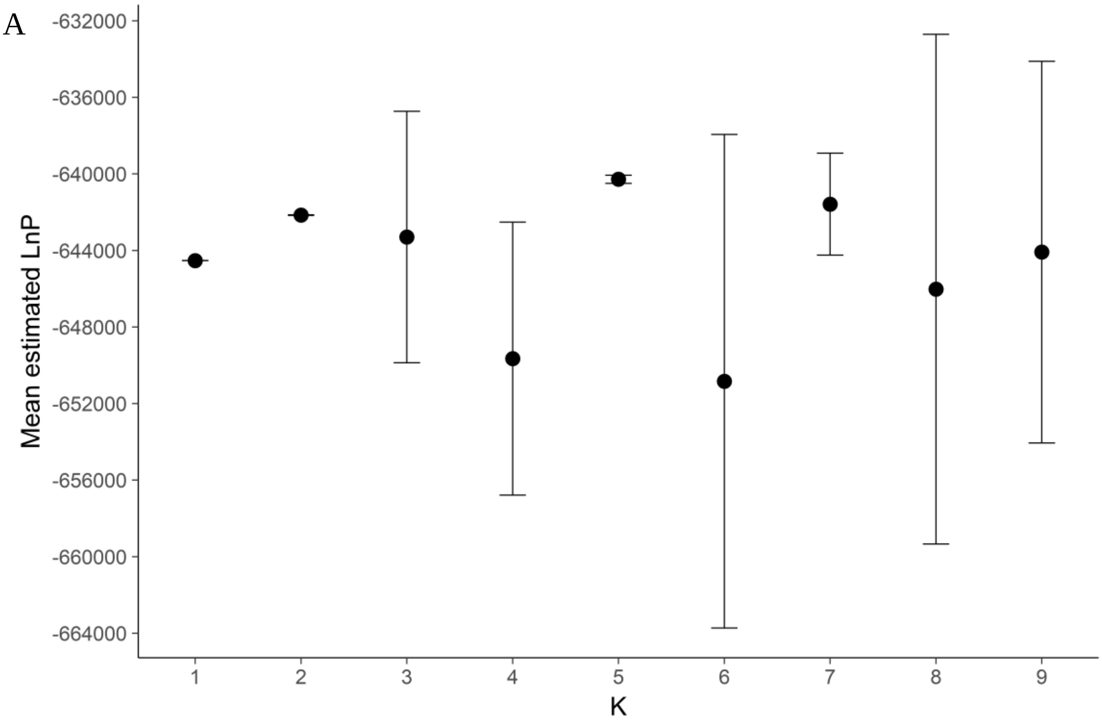

B

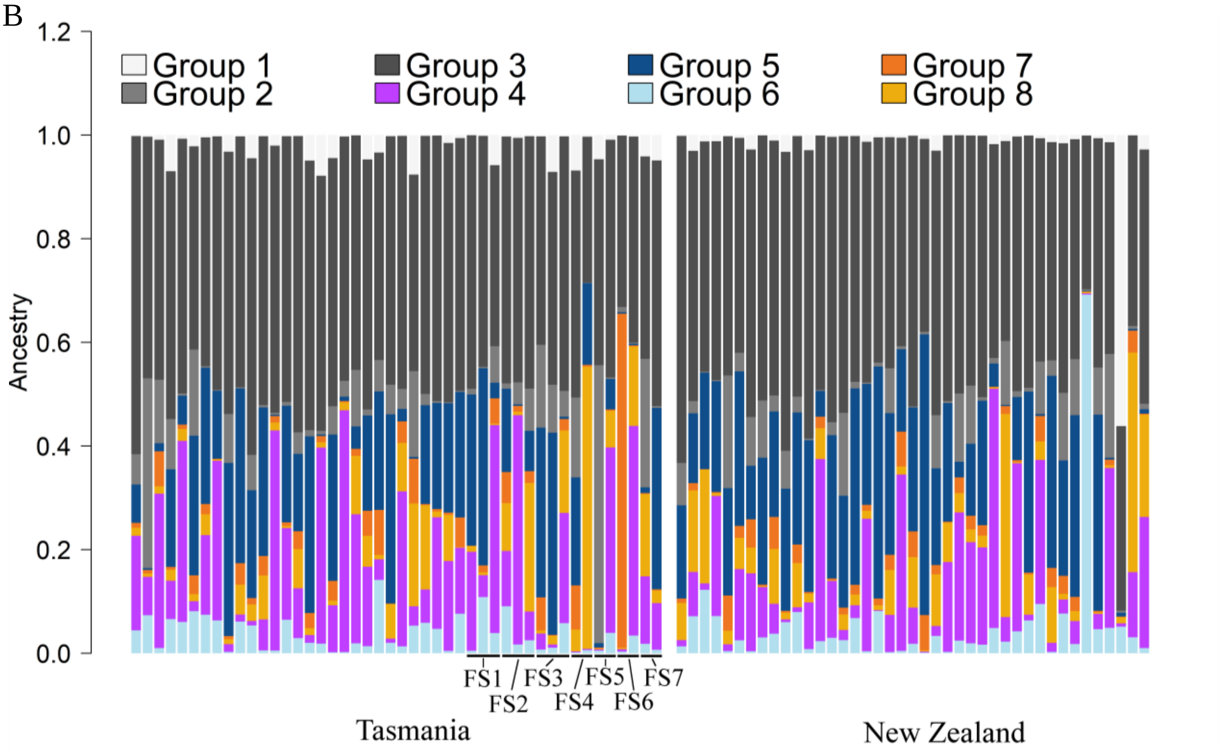

S4. STRUCTURE results for the dataset with full siblings excluded, n = 76 & 6,587 SNPs. A) Optimal number of clusters selection, based on the mean estimated ln probability for K ranging from one to nine. B) STRUCTURE ancestry plot between Tasmania and New Zealand, based on K = 2.

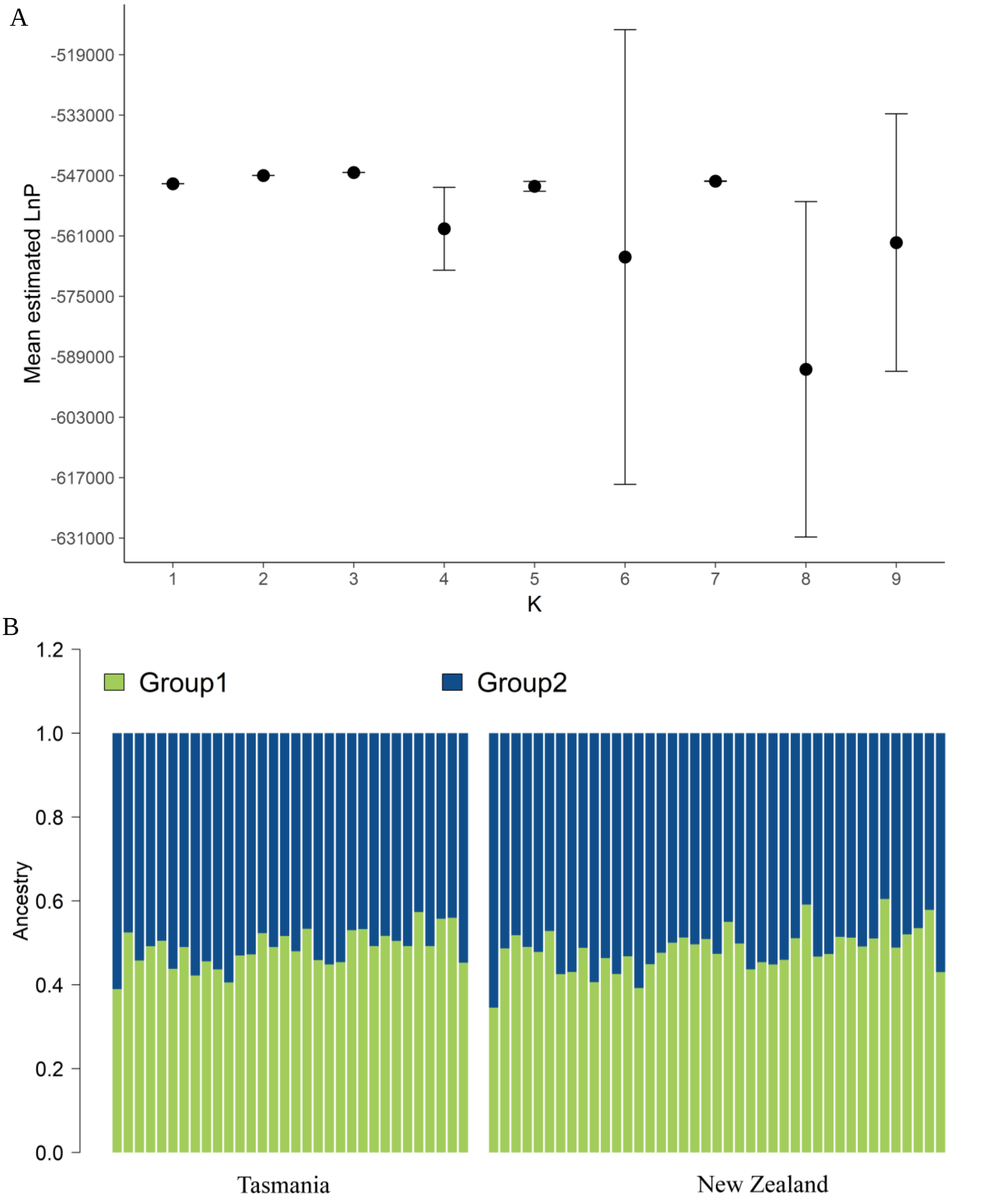

S5. Kinship results of COLONY2 for nine Tasmanian and 20 New Zealand school sharks from Bester-van der Merwe et al. *(2017), genotyped for 19 microsatellite markers. Probable kin are indicated in bold.*

Full sibling pairs		
OffspringID1	OffspringID2	Probability
**AUS3**	**AUS7**	**0.994**
**NZ34**	**NZ35**	**0.76**
AUS4	AUS8	0.401
AUS6	AUS7	0.383
AUS7	AUS8	0.268
AUS1	AUS3	0.202
AUS4	AUS6	0.199
AUS3	AUS8	0.179
AUS1	AUS7	0.117
AUS6	AUS9	0.082
AUS3	AUS6	0.065
NZ19	NZ20	0.045
NZ7	NZ12	0.004
AUS4	AUS7	0.003
AUS1	AUS5	0.002
AUS4	AUS9	0.001

Half sibling pairs		
OffspringID1	OffspringID2	Probability
**AUS6**	**AUS8**	**0.834**
**NZ6**	**NZ15**	**0.826**
**AUS1**	**AUS6**	**0.819**
**AUS1**	**AUS4**	**0.786**
**NZ42**	**NZ50**	**0.781**
**AUS8**	**AUS9**	**0.761**
AUS3	AUS4	0.74
AUS3	AUS9	0.722
NZ21	NZ30	0.71
AUS1	AUS2	0.703
NZ21	NZ42	0.646
AUS2	AUS5	0.626
AUS1	AUS9	0.563
AUS7	AUS9	0.542
NZ19	NZ20	0.46
AUS1	AUS8	0.445
NZ7	NZ13	0.411
AUS1	AUS7	0.403
AUS3	AUS6	0.388
AUS5	AUS8	0.372
AUS1	NZ2	0.367
AUS5	NZ2	0.303
AUS4	AUS9	0.286
AUS3	AUS8	0.248
AUS2	AUS3	0.24
AUS1	AUS3	0.215
AUS2	AUS7	0.203
AUS6	AUS9	0.198
NZ19	NZ35	0.146
NZ7	NZ12	0.123
AUS4	AUS5	0.122
AUS4	AUS8	0.121
AUS4	AUS6	0.087
NZ12	NZ20	0.084

NZ19	NZ34	0.081
AUS6	AUS7	0.073
NZ20	NZ38	0.072
AUS4	AUS7	0.068
NZ36	NZ38	0.063
NZ30	NZ38	0.061
AUS2	NZ2	0.056
NZ20	NZ45	0.051
NZ17	NZ19	0.05
AUS5	AUS6	0.049
AUS8	NZ2	0.044
AUS4	NZ2	0.043
AUS1	AUS5	0.036
AUS6	NZ2	0.035
NZ17	NZ20	0.017
NZ34	NZ35	0.016
NZ38	NZ45	0.016
NZ35	NZ36	0.012
NZ30	NZ42	0.01
NZ13	NZ14	0.009
NZ20	NZ21	0.008
NZ20	NZ42	0.008
AUS4	NZ38	0.006
AUS7	AUS8	0.006
NZ20	NZ30	0.006
NZ19	NZ38	0.005
AUS3	AUS5	0.002
AUS5	AUS7	0.002
AUS5	AUS9	0.002
AUS6	NZ38	0.002
NZ2	NZ13	0.002
